# Supplementary material for: Reaction of Picolinamides with Ketones Producing a New Type of Heterocyclic Salts with an Imidazolidin-4-One Ring
Source: Molecules. 2023 Dec 29;29(1):206. doi: 10.3390/molecules29010206 (PMC10780162; doi:10.3390/molecules29010206)

Supporting Information

Reaction of Picolinamides With Ketones Producing a New Type of Heterocyclic Salts With a Imidazolidin-4-one Ring

Eugene P. Kramarova[1], Dmitri N. Lyakhmun[1], Dmitry V. Tarasenko[1], Sophia S. Borisevich[2], Edward M. Khamitov[2] Alfia R. Yusupova[2], Alexander A. Korlyukov[1,3], Alexander R. Romanenko[3,4], Tatiana A. Shmigol[1], Yuri I. Baukov[1] and Vadim V. Negrebetsky[1]*

[1] Department of Medicinal Chemistry and Toxicology, Pirogov Russian National Research Medical University, 117997 Moscow, Russia

[2] Ufa Institute of Chemistry UFRS RAS, pr. Oktyabrya 71, 450054 Ufa, Russia

[3] A.N.Nesmeyanov Institute of Organoelement Compounds of Russian Academy of Sciences (INEOS RAS), Russia, 119334, Moscow, Vavilova St. 28, bld. 1

[4] Federal State Budgetary Educational Institution of Higher Education "Russian University of Chemical Technology named after D.I. Mendeleev", 125047, Moscow, Miusskaya square, 9

A Table of Contents

Figure S1. Molecular dynamics box with a system containing ortho-pyridinecarboxamide-1,2 (black), sulfonate (blue), acetone (red) and methanol (blue). 2

Table S1. The enthalpy ($\Delta_rH°$) and Gibbs free energy ($\Delta_rG°$) of the reaction of formation of oxysulfonic acids 3

Figure S2. Proton migration transition state 3

S1. XYZ coordinates of the stationary points of 2a-f products; calculation by the M052X-D3/TZVP + IEFPCM approximation. 5

Table S2. The enthalpy ($\Delta_rH°$) and Gibbs free energy ($\Delta_rG°$) of the formation reaction of salt 2a-2g 11

S2. XYZ coordinates of the stationary points of 3a-i products; calculation by the M052X-D3/TZVP + IEFPCM approximation. 12

Table S3. The enthalpy ($\Delta_rH°$) and Gibbs free energy ($\Delta_rG°$) of the formation reaction of salt 3a-3k 26

Table S4. ^{13}C NMR chemical shifts (theoretical calculations). 27

^{1}H, ^{13}C NMR experimental data 33

X-ray diffraction data 67

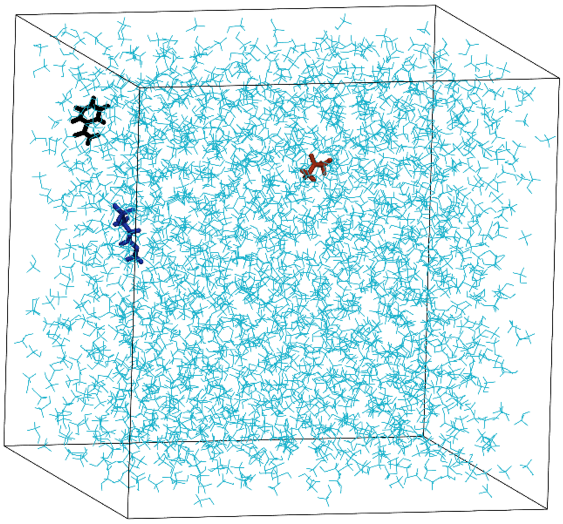

Figure S1. Molecular dynamics box with a system containing ortho-pyridinecarboxamide-1,2 (black), sulfonate (blue), acetone (red) and methanol (blue). Cube verge is equal to 5.285 nm

Table S1. The enthalpy ($\Delta_rH°$) and Gibbs free energy ($\Delta_rG°$) of the reaction of formation of oxysulfonic acids

Product ID	Reagent structure	Product structure	$\Delta_rH°$, kJ/mol	$\Delta_rG°$, kJ/mol
**1-IIa**	CH_3-OH	$CH_3O(CH_2)_3SO_3H$	-28.6	11.2
**1-IIb**	C_2H_5-OH	$C_2H_5O(CH_2)_3SO_3H$	-26.5	16.7
**1-IIc**	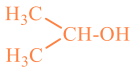	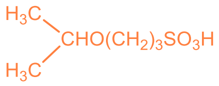	-25.6	14.8
**1-IId**	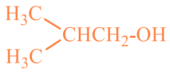	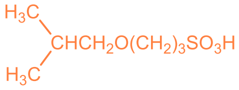	-31.9	11.3
**1-IIe**	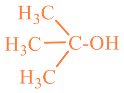	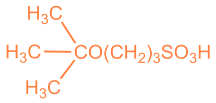	-22.2	17.4
**1-IIf**	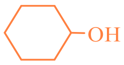	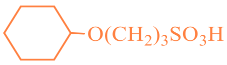	-21.6	17.9

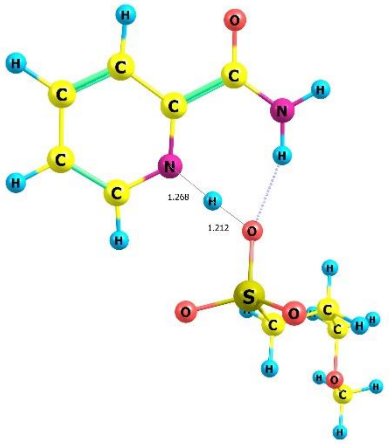

Figure S2. Proton migration transition state

Table S2. AIM analysis results

<table>
<tr><td rowspan="5">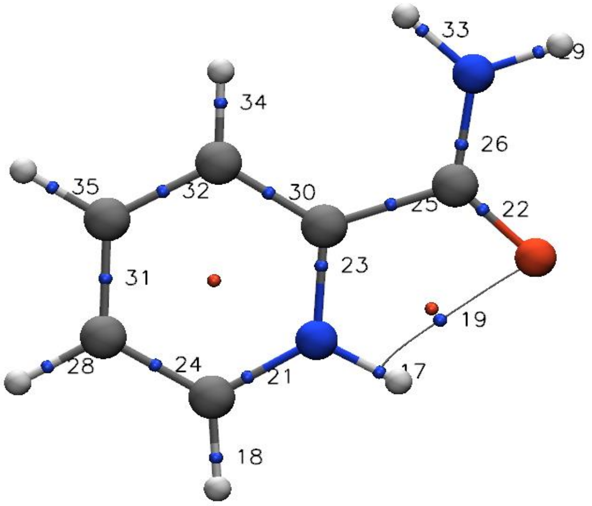
</td><td colspan="2">CP19, Type (3,-1)</td></tr>
<tr><td>Density of all electrons:</td><td>0.023548</td></tr>
<tr><td>Connected atoms:</td><td>8(O)—1(N)</td></tr>
<tr><td>Neutral Binding Energy, kJ/mol</td><td>-18.87</td></tr>
<tr><td>Charged Binding Energy, kJ/mol</td><td>-37.20</td></tr>
<tr><td rowspan="15">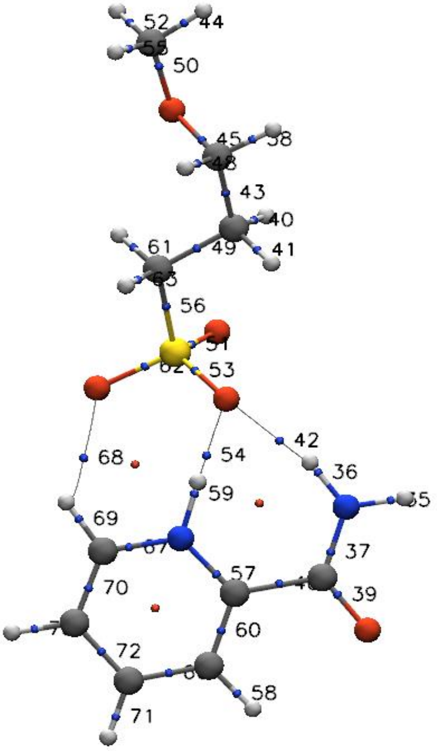
</td><td colspan="2">CP68, Type (3,-1)</td></tr>
<tr><td>Density of all electrons:</td><td>0.013222</td></tr>
<tr><td>Connected atoms:</td><td>25(O)—14(H)</td></tr>
<tr><td>Neutral Binding Energy, kJ/mol</td><td>-9.24</td></tr>
<tr><td>Charged Binding Energy, kJ/mol</td><td>-22.85</td></tr>
<tr><td colspan="2">CP54, Type (3,-1)</td></tr>
<tr><td>Density of all electrons:</td><td>0.062928</td></tr>
<tr><td>Connected atoms:</td><td>24(O)—7(H)</td></tr>
<tr><td>Neutral Binding Energy, kJ/mol</td><td>-55.63</td></tr>
<tr><td>Charged Binding Energy, kJ/mol</td><td>-91.96</td></tr>
<tr><td colspan="2">CP42, Type (3,-1)</td></tr>
<tr><td>Density of all electrons:</td><td>0.031711</td></tr>
<tr><td>Connected atoms:</td><td>16(H)—24(O)</td></tr>
<tr><td>Neutral Binding Energy, kJ/mol</td><td>-26.49</td></tr>
<tr><td>Charged Binding Energy, kJ/mol</td><td>-48.56</td></tr>
</table>

S1. XYZ coordinates of the stationary points of 2a-f products; calculation by the M052X-D3/TZVP + IEFPCM approximation.

2a
Solvent=Methanol

7	2.330931000	0.468397000	-0.263583000
6	3.384064000	-0.379191000	-0.238018000
6	4.666036000	0.142566000	-0.355718000
6	4.841050000	1.520738000	-0.488698000
6	3.729380000	2.363537000	-0.497987000
6	2.466753000	1.801023000	-0.381311000
1	1.330844000	0.106274000	-0.200641000
6	3.208935000	-1.880897000	-0.073139000
8	4.210017000	-2.591284000	-0.167467000
7	1.974363000	-2.347195000	0.186009000
1	5.500271000	-0.545943000	-0.337293000
1	5.839959000	1.932882000	-0.579166000
1	3.831394000	3.437247000	-0.592082000
1	1.540183000	2.361299000	-0.356549000
1	1.886609000	-3.348587000	0.286309000
1	1.127277000	-1.780343000	0.248187000
6	-6.715109000	0.251016000	-0.711788000
8	-5.438934000	0.057055000	-0.126796000
6	-4.587267000	-0.752328000	-0.925682000
6	-3.233053000	-0.852485000	-0.236229000
6	-2.570383000	0.516574000	-0.096252000
16	-0.991098000	0.441303000	0.774682000
8	-1.240645000	-0.141569000	2.114281000
8	-0.135672000	-0.479003000	-0.103957000
8	-0.431243000	1.820952000	0.774299000
1	-7.245898000	-0.702356000	-0.857396000
1	-7.295124000	0.877760000	-0.030125000
1	-6.643657000	0.756671000	-1.687017000
1	-5.020075000	-1.757727000	-1.054043000
1	-4.474525000	-0.313502000	-1.931749000
1	-3.365262000	-1.295717000	0.755954000
1	-2.584834000	-1.518904000	-0.813743000
1	-2.356918000	0.966094000	-1.070176000
1	-3.200348000	1.203073000	0.473706000

2b
Solvent=Ethanol

7	-2.735428000	-0.441999000	-0.285746000
6	-3.771034000	0.427138000	-0.273070000
6	-5.060012000	-0.065132000	-0.434011000
6	-5.259982000	-1.436696000	-0.596719000
6	-4.166155000	-2.302534000	-0.591917000
6	-2.895722000	-1.769110000	-0.431253000
1	-1.728868000	-0.102264000	-0.190412000
6	-3.569233000	1.921894000	-0.077343000

8	-4.552821000	2.654604000	-0.183719000
7	-2.332112000	2.357555000	0.220199000
1	-5.879888000	0.640611000	-0.425214000
1	-6.264473000	-1.825773000	-0.721467000
1	-4.287743000	-3.372024000	-0.708369000
1	-1.981848000	-2.349188000	-0.391812000
1	-2.226484000	3.354953000	0.340953000
1	-1.498597000	1.772002000	0.292686000
8	5.029077000	-0.187539000	0.044518000
6	4.219341000	0.656654000	-0.764573000
6	2.848400000	0.767501000	-0.108732000
6	2.157818000	-0.591283000	-0.014329000
16	0.562410000	-0.505705000	0.824784000
8	0.793716000	0.045188000	2.180943000
8	-0.257867000	0.448242000	-0.052454000
8	-0.022940000	-1.874108000	0.783205000
1	4.661269000	1.658711000	-0.863813000
1	4.125631000	0.235809000	-1.779917000
1	2.962555000	1.186869000	0.896049000
1	2.227998000	1.458904000	-0.687515000
1	1.957227000	-1.015068000	-1.002447000
1	2.764092000	-1.300904000	0.552924000
6	6.264276000	-0.575796000	-0.553203000
1	6.645708000	-1.387843000	0.074529000
1	6.081939000	-0.990702000	-1.557706000
6	7.289001000	0.554023000	-0.624913000
1	7.453018000	0.981580000	0.369098000
1	6.965255000	1.355131000	-1.295982000
1	8.243912000	0.170517000	-0.998858000

2c
Solvent=2-Propanol

7	-3.054421000	-0.475709000	-0.294585000
6	-4.128701000	0.343547000	-0.243845000
6	-5.396658000	-0.205056000	-0.388997000
6	-5.536242000	-1.581242000	-0.574027000
6	-4.403630000	-2.395157000	-0.607184000
6	-3.156125000	-1.806010000	-0.462064000
1	-2.062548000	-0.090833000	-0.212707000
6	-3.992614000	1.841852000	-0.020118000
8	-5.011076000	2.529572000	-0.092412000
7	-2.771400000	2.328309000	0.264243000
1	-6.247767000	0.461663000	-0.349927000
1	-6.524095000	-2.014354000	-0.686006000
1	-4.478131000	-3.466947000	-0.741110000
1	-2.215688000	-2.343275000	-0.451530000
1	-2.709964000	3.326776000	0.405005000
1	-1.910066000	1.781048000	0.307176000
8	4.690228000	0.128593000	0.001361000
6	3.850897000	0.946676000	-0.801986000
6	2.473422000	0.990028000	-0.150521000
6	1.845825000	-0.400033000	-0.075591000

16	0.251835000	-0.403422000	0.770043000
8	0.461385000	0.136736000	2.133829000
8	-0.617255000	0.523874000	-0.089468000
8	-0.268140000	-1.797236000	0.707988000
1	4.249653000	1.968058000	-0.887762000
1	3.778292000	0.534814000	-1.822527000
1	2.566297000	1.400672000	0.860050000
1	1.824296000	1.660496000	-0.722393000
1	1.661365000	-0.817229000	-1.069641000
1	2.487551000	-1.088839000	0.478165000
6	5.932007000	-0.257786000	-0.600449000
1	5.728370000	-0.637814000	-1.616022000
6	6.479899000	-1.391660000	0.257769000
6	6.906125000	0.918587000	-0.694661000
1	6.515041000	1.719014000	-1.329159000
1	7.094237000	1.328740000	0.303232000
1	7.859433000	0.590359000	-1.120687000
1	6.674201000	-1.034479000	1.274565000
1	7.415951000	-1.774167000	-0.159539000
1	5.758591000	-2.211918000	0.312165000

2d
Solvent=2-Methyl-1-Propanol

7	3.344435000	0.539725000	-0.238077000
6	4.465655000	-0.211655000	-0.161172000
6	5.701069000	0.419642000	-0.233372000
6	5.760170000	1.806960000	-0.372855000
6	4.580230000	2.548860000	-0.434163000
6	3.367964000	1.878331000	-0.362996000
1	2.375845000	0.091029000	-0.212554000
6	4.416376000	-1.721616000	0.016305000
8	5.479277000	-2.341061000	-0.026911000
7	3.217603000	-2.292008000	0.232136000
1	6.590447000	-0.193485000	-0.174277000
1	6.722293000	2.304345000	-0.427600000
1	4.591759000	3.626912000	-0.533710000
1	2.395516000	2.354949000	-0.380047000
1	3.214255000	-3.296065000	0.343516000
1	2.321601000	-1.801782000	0.246961000
8	-4.339424000	-0.516442000	-0.443445000
6	-3.392704000	-1.271747000	-1.181345000
6	-2.073602000	-1.258441000	-0.420101000
6	-1.537939000	0.162140000	-0.253006000
16	0.008682000	0.218661000	0.675686000
8	-0.235197000	-0.396791000	2.000970000
8	0.973160000	-0.616029000	-0.177767000
8	0.446597000	1.641327000	0.701542000
1	-3.742189000	-2.308623000	-1.313283000
1	-3.259184000	-0.840792000	-2.188342000
1	-2.221548000	-1.708297000	0.566671000
1	-1.341178000	-1.868916000	-0.957518000
1	-1.327790000	0.633580000	-1.217305000

1	-2.246090000	0.788536000	0.293870000
6	-5.604973000	-0.410293000	-1.083038000
1	-5.487009000	0.050803000	-2.078249000
1	-6.037071000	-1.413990000	-1.234231000
6	-6.532576000	0.440333000	-0.213395000
1	-7.490132000	0.489402000	-0.751099000
6	-5.989728000	1.867704000	-0.056675000
6	-6.773843000	-0.221448000	1.150487000
1	-7.200527000	-1.224035000	1.034996000
1	-5.831717000	-0.317860000	1.698609000
1	-7.464128000	0.372683000	1.758162000
1	-5.021710000	1.854125000	0.453505000
1	-6.676474000	2.485828000	0.530751000
1	-5.850045000	2.348426000	-1.031349000

2e
Solvent=2-Methyl-2-Propanol

7	-3.306653000	-0.504861000	-0.278788000
6	-4.394294000	0.297652000	-0.288439000
6	-5.652374000	-0.280186000	-0.404608000
6	-5.768157000	-1.667690000	-0.498976000
6	-4.622187000	-2.463045000	-0.471357000
6	-3.385544000	-1.844761000	-0.358091000
1	-2.319603000	-0.098830000	-0.214195000
6	-4.283851000	1.809596000	-0.162313000
8	-5.312051000	2.474163000	-0.289663000
7	-3.073205000	2.332740000	0.101684000
1	-6.514189000	0.373804000	-0.414633000
1	-6.748078000	-2.123742000	-0.587768000
1	-4.678335000	-3.542510000	-0.533940000
1	-2.436526000	-2.364263000	-0.304940000
1	-3.029121000	3.338991000	0.179089000
1	-2.204193000	1.802843000	0.190263000
8	4.410300000	0.308716000	0.202267000
6	3.584773000	1.038288000	-0.696275000
6	2.184982000	1.088772000	-0.093057000
6	1.590178000	-0.310337000	0.048903000
16	-0.032072000	-0.304208000	0.838116000
8	0.112828000	0.331919000	2.167941000
8	-0.893493000	0.539841000	-0.112589000
8	-0.515082000	-1.712436000	0.850303000
1	3.963628000	2.060708000	-0.837799000
1	3.544565000	0.562501000	-1.686743000
1	2.234254000	1.562234000	0.892684000
1	1.539054000	1.705174000	-0.726278000
1	1.452337000	-0.794953000	-0.921907000
1	2.230075000	-0.943543000	0.667259000
6	5.683362000	-0.175477000	-0.282973000
6	5.468975000	-1.318705000	-1.286402000
6	6.369324000	-0.702179000	0.980202000
1	6.506142000	0.108273000	1.702093000
1	5.754570000	-1.478056000	1.446268000

1	7.347860000	-1.128554000	0.741133000
1	4.847762000	-2.099536000	-0.837568000
1	6.429378000	-1.758796000	-1.571891000
1	4.980719000	-0.971258000	-2.201518000
6	6.506839000	0.960236000	-0.905732000
1	6.590520000	1.796183000	-0.204340000
1	7.514300000	0.606994000	-1.145033000
1	6.054880000	1.327905000	-1.831734000

2f
Solvent=1-Hexanol

7	3.911805000	0.574994000	-0.281122000
6	5.002461000	-0.185481000	-0.525130000
6	6.231864000	0.439988000	-0.691000000
6	6.317707000	1.829504000	-0.593620000
6	5.171301000	2.578357000	-0.326356000
6	3.963159000	1.914164000	-0.172004000
1	2.943083000	0.134718000	-0.172760000
6	4.927169000	-1.702373000	-0.611204000
8	5.943834000	-2.310826000	-0.944727000
7	3.760523000	-2.294326000	-0.298417000
1	7.095937000	-0.180017000	-0.889717000
1	7.275404000	2.322289000	-0.720584000
1	5.205689000	3.656810000	-0.236553000
1	3.017960000	2.392090000	0.056122000
1	3.739234000	-3.302050000	-0.365539000
1	2.899756000	-1.809540000	-0.039076000
8	-3.714622000	-0.608279000	0.772346000
6	-2.938308000	-1.192351000	-0.269096000
6	-1.490502000	-1.239527000	0.205942000
6	-0.938087000	0.163334000	0.447304000
16	0.749321000	0.149614000	1.085304000
8	0.750344000	-0.623321000	2.348838000
8	1.547121000	-0.554285000	-0.021723000
8	1.180984000	1.569632000	1.202493000
1	-3.288466000	-2.209929000	-0.494127000
1	-3.005717000	-0.608597000	-1.198235000
1	-1.433903000	-1.815563000	1.135142000
1	-0.880436000	-1.755399000	-0.542309000
1	-0.908358000	0.753178000	-0.473308000
1	-1.540849000	0.697724000	1.184822000
6	-5.010507000	-0.104325000	0.420547000
6	-5.900415000	-1.135777000	-0.284788000
6	-7.307965000	-0.564347000	-0.523600000
6	-7.247480000	0.749960000	-1.316921000
6	-6.340448000	1.776774000	-0.621789000
6	-4.933997000	1.204752000	-0.378211000
1	-5.464844000	0.123842000	1.394362000
1	-5.943219000	-2.051510000	0.315914000
1	-5.455481000	-1.402827000	-1.252186000
1	-7.926770000	-1.301315000	-1.047954000
1	-7.792551000	-0.379149000	0.445215000

1	-8.255238000	1.160752000	-1.448671000
1	-6.854975000	0.545161000	-2.323015000
1	-6.274518000	2.695584000	-1.215471000
1	-6.787610000	2.057188000	0.342276000
1	-4.306245000	1.919649000	0.165234000
1	-4.450319000	1.019142000	-1.345930000

2g

Solvent=Methanol

7	-2.230219000	-0.730851000	-0.270231000
6	-3.297795000	0.081465000	-0.299315000
6	-4.552884000	-0.464107000	-0.453860000
6	-4.685797000	-1.841501000	-0.570416000
6	-3.562607000	-2.650691000	-0.527559000
6	-2.326912000	-2.058340000	-0.373796000
1	-1.253571000	-0.347940000	-0.159032000
6	-3.158030000	1.583228000	-0.164779000
8	-4.148480000	2.274558000	-0.368025000
7	-1.972342000	2.073318000	0.181489000
1	-5.401350000	0.198769000	-0.479657000
1	-5.665105000	-2.278386000	-0.689767000
1	-3.633731000	-3.722365000	-0.610569000
1	-1.393119000	-2.594921000	-0.311930000
1	-1.164368000	1.484147000	0.343315000
6	6.726894000	-0.079511000	-0.986095000
8	5.500148000	-0.048119000	-0.279093000
6	4.556650000	0.802011000	-0.907569000
6	3.268299000	0.770622000	-0.111997000
6	2.690598000	-0.634381000	-0.077275000
16	1.158730000	-0.701969000	0.846993000
8	1.436147000	-0.233352000	2.226795000
8	0.215608000	0.245836000	0.111526000
8	0.642857000	-2.097551000	0.757070000
1	7.171113000	0.917135000	-1.034767000
1	7.395667000	-0.746376000	-0.451809000
1	6.578302000	-0.452100000	-2.001985000
1	4.946427000	1.822654000	-0.948217000
1	4.381394000	0.462068000	-1.933629000
1	3.462826000	1.116353000	0.901772000
1	2.554660000	1.451736000	-0.572836000
1	2.448584000	-1.001396000	-1.072614000
1	3.360076000	-1.334072000	0.415707000
6	-1.797494000	3.510854000	0.313689000
1	-2.435117000	3.899526000	1.104012000
1	-0.758860000	3.701660000	0.557639000
1	-2.051376000	4.008868000	-0.618670000

Table S3. The enthalpy ($\Delta_r H°$) and Gibbs free energy ($\Delta_r G°$) of the formation reaction of salt 2a-2g

Reagent id	Reagent structure	Product id	Product structure	Yield, %	ΔH, kJ/mol	ΔG, kJ/mol
**1**	CH_3-OH	**2a**	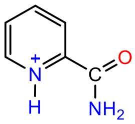 • $CH_3O(CH_2)_3SO_3^-$	65	-88.8	-2.0
**1**	C_2H_5-OH	**2b**	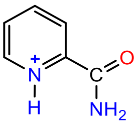 • $C_2H_5O(CH_2)_3SO_3^-$	85	-87.1	2.4
**1**	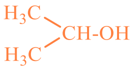	**2c**	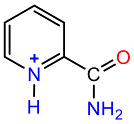 • $(CH_3)_2CHO(CH_2)_3SO_3^-$	59	-85.1	4.5
**1**	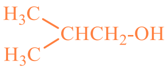	**2d**	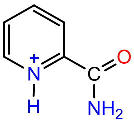 • $(CH_3)_2CHCH_2O(CH_2)_3SO_3^-$	57	-89.8	-0.9
**1**	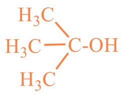	**2e**	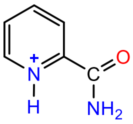 • $(CH_3)_3CO(CH_2)_3SO_3^-$	25	-79.8	10.8
**1**	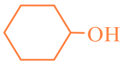	**2f**	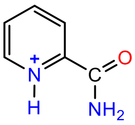 • 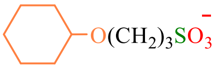	89	-82.2	9.3
**1**	CH_3-OH	**2g**	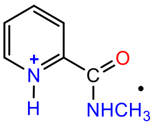 • $CH_3O(CH_2)_3SO_3^-$	81	1.2	20.1

S2. XYZ coordinates of the stationary points of 3a-i products; calculation by the M052X-D3/TZVP + IEFPCM approximation.

3a

Solvent=Methanol

7	3.616002000	-0.395441000	-0.148365000
6	3.836291000	0.926440000	-0.001083000
6	5.115032000	1.447092000	-0.052307000
6	6.170913000	0.558343000	-0.260530000
6	5.914665000	-0.810219000	-0.409862000
6	4.610868000	-1.278158000	-0.350233000
6	2.516624000	1.607998000	0.208659000
8	2.356549000	2.809911000	0.372442000
7	1.604061000	0.610790000	0.172324000
1	5.258556000	2.513389000	0.069969000
1	7.190034000	0.924967000	-0.306428000
1	6.718908000	-1.516466000	-0.571973000
1	4.342085000	-2.320686000	-0.456177000
1	0.574655000	0.747851000	0.263419000
6	-7.720032000	0.071401000	-0.242203000
8	-6.328813000	-0.112254000	-0.437088000
6	-5.602524000	1.108469000	-0.385488000
6	-4.121324000	0.797054000	-0.551594000
6	-3.594242000	-0.087193000	0.576039000
16	-1.849015000	-0.519164000	0.370211000
8	-1.738863000	-1.236251000	-0.934699000
8	-1.124347000	0.807399000	0.361719000
8	-1.491105000	-1.358100000	1.551218000
1	-8.157893000	0.722687000	-1.014417000
1	-8.191505000	-0.912629000	-0.301583000
1	-7.939254000	0.512269000	0.742541000
1	-5.938179000	1.788911000	-1.185160000
1	-5.782281000	1.619928000	0.575697000
1	-3.963630000	0.292731000	-1.510489000
1	-3.558837000	1.735723000	-0.576358000
1	-3.673980000	0.408538000	1.548069000
1	-4.141381000	-1.031382000	0.623405000
6	2.142232000	-0.721332000	-0.052236000
6	1.892943000	-1.643182000	1.143556000
1	0.813039000	-1.752308000	1.275581000
1	2.330244000	-2.629278000	0.968587000
1	2.319635000	-1.210349000	2.051125000
6	1.652930000	-1.312689000	-1.376129000
1	0.565891000	-1.420502000	-1.322666000
1	1.907210000	-0.647608000	-2.204546000
1	2.096865000	-2.296392000	-1.547961000

3b

Solvent=Methanol

7	-3.528487000	-0.308452000	-0.375229000
6	-3.817339000	0.960762000	-0.024132000

6	-5.124723000	1.401084000	0.051851000
6	-6.136537000	0.487153000	-0.245917000
6	-5.809530000	-0.825665000	-0.607415000
6	-4.479679000	-1.213409000	-0.668329000
6	-2.530566000	1.684573000	0.239107000
8	-2.431183000	2.854947000	0.581361000
7	-1.565315000	0.764710000	0.011848000
6	-2.037063000	-0.556080000	-0.369457000
6	-1.565195000	-0.937836000	-1.773823000
6	-1.687258000	-1.630473000	0.679189000
6	-2.127140000	-1.297363000	2.104314000
1	-5.323058000	2.426423000	0.338047000
1	-7.176130000	0.790446000	-0.197179000
1	-6.579033000	-1.550331000	-0.840931000
1	-4.156598000	-2.210141000	-0.937267000
1	-0.545663000	0.940242000	0.140826000
1	-1.892303000	-0.190448000	-2.500055000
1	-0.472320000	-0.977471000	-1.765350000
1	-1.952331000	-1.918774000	-2.060582000
1	-2.122454000	-2.579523000	0.346989000
1	-0.599397000	-1.746060000	0.645679000
1	-1.836050000	-2.107215000	2.777965000
1	-1.650291000	-0.379065000	2.457163000
1	-3.212189000	-1.174509000	2.184906000
6	7.693916000	1.046697000	-0.555470000
8	6.375381000	0.539003000	-0.660712000
6	6.072288000	-0.411851000	0.351260000
6	4.624807000	-0.852568000	0.181754000
6	3.650510000	0.311073000	0.351145000
16	1.928911000	-0.192882000	0.109818000
8	1.821600000	-0.687258000	-1.294948000
8	1.645669000	-1.238694000	1.137030000
8	1.137559000	1.073458000	0.339115000
1	8.447015000	0.248935000	-0.648342000
1	7.835106000	1.761928000	-1.369472000
1	7.857128000	1.562127000	0.403585000
1	6.744482000	-1.282097000	0.274109000
1	6.224480000	0.031284000	1.350367000
1	4.495703000	-1.291528000	-0.812978000
1	4.399154000	-1.631199000	0.917533000
1	3.706328000	0.738861000	1.356574000
1	3.851282000	1.102189000	-0.374231000

3c
Solvent=Methanol

7	-3.174671000	0.282010000	-0.257657000
6	-3.267649000	-0.262191000	-1.489078000
6	-4.487593000	-0.412898000	-2.119025000
6	-5.629044000	0.012341000	-1.438110000
6	-5.510956000	0.553877000	-0.152994000
6	-4.260105000	0.679742000	0.432675000
6	-1.897625000	-0.678555000	-1.929774000

8	-1.635207000	-1.259401000	-2.974818000
7	-1.077232000	-0.291815000	-0.927140000
1	-4.521221000	-0.858318000	-3.105461000
1	-6.606652000	-0.086917000	-1.895857000
1	-6.383141000	0.872979000	0.403244000
1	-4.106362000	1.069145000	1.428410000
1	-0.098077000	-0.640215000	-0.829100000
6	7.501401000	1.073874000	-0.727543000
8	6.126663000	0.919281000	-0.422003000
6	5.385904000	0.359260000	-1.498243000
6	3.939995000	0.189679000	-1.052753000
6	3.814103000	-0.768432000	0.129417000
16	2.105484000	-0.938540000	0.703460000
8	1.658587000	0.432202000	1.100489000
8	1.343792000	-1.451889000	-0.498264000
8	2.137637000	-1.914610000	1.826344000
1	7.655498000	1.743567000	-1.587783000
1	7.984675000	1.509961000	0.150186000
1	7.979877000	0.108681000	-0.954636000
1	5.434892000	1.017433000	-2.381204000
1	5.814780000	-0.614112000	-1.791875000
1	3.532260000	1.166213000	-0.772283000
1	3.347110000	-0.185622000	-1.892943000
1	4.152323000	-1.775971000	-0.130709000
1	4.395916000	-0.418147000	0.984832000
6	-1.730797000	0.360886000	0.198324000
6	-1.251929000	1.830008000	0.261618000
1	-0.163984000	1.746396000	0.352852000
1	-1.464235000	2.286264000	-0.712007000
6	-1.786351000	2.720877000	1.388418000
1	-1.626526000	2.241618000	2.360262000
1	-2.865593000	2.878639000	1.280967000
6	-1.087913000	4.085896000	1.379238000
1	-1.243197000	4.601260000	0.425153000
1	-0.008610000	3.972739000	1.526856000
1	-1.473050000	4.728669000	2.176291000
6	-1.536515000	-0.407828000	1.516312000
1	-0.508170000	-0.201212000	1.826383000
1	-2.188895000	0.030727000	2.278925000
6	-1.766264000	-1.919828000	1.431833000
1	-1.127867000	-2.335503000	0.646208000
1	-2.805037000	-2.130518000	1.146898000
6	-1.435432000	-2.602798000	2.762135000
1	-2.038887000	-2.196199000	3.581504000
1	-0.378629000	-2.455187000	3.006827000
1	-1.624777000	-3.679230000	2.707590000

3d

Solvent=Methanol

**7**	2.943115000	0.483539000	0.077214000
6	2.354153000	1.640310000	-0.291284000

6	3.027137000	2.844082000	-0.208728000
6	4.337957000	2.825187000	0.268874000
6	4.926808000	1.611553000	0.645788000
6	4.205585000	0.432550000	0.542864000
6	0.960437000	1.334688000	-0.747356000
8	0.142990000	2.160970000	-1.136995000
7	0.840347000	-0.003743000	-0.615891000
6	2.017820000	-0.681483000	-0.103745000
6	2.561283000	-1.733706000	-1.092989000
6	1.744206000	-1.465117000	1.215243000
6	1.723787000	-2.968037000	-0.733244000
6	1.722073000	-2.961185000	0.809364000
1	2.523659000	3.753551000	-0.511947000
1	4.901987000	3.747273000	0.350531000
1	5.941752000	1.574226000	1.020377000
1	4.599984000	-0.536271000	0.819464000
1	-0.034209000	-0.535699000	-0.836009000
1	2.471200000	-1.391539000	-2.125981000
1	3.619422000	-1.921533000	-0.878355000
1	2.519550000	-1.248204000	1.954412000
1	0.783073000	-1.145610000	1.617194000
1	0.703467000	-2.842272000	-1.109533000
1	2.136401000	-3.885611000	-1.159853000
1	2.616006000	-3.467291000	1.187900000
1	0.841352000	-3.458173000	1.218028000
6	-5.165108000	3.305757000	-0.050209000
8	-4.240499000	2.250053000	-0.242204000
6	-3.473012000	1.975366000	0.922518000
6	-2.544712000	0.806654000	0.623908000
6	-3.316230000	-0.466794000	0.285376000
16	-2.226463000	-1.876058000	-0.043770000
8	-1.374839000	-1.442182000	-1.220449000
8	-1.414354000	-2.069211000	1.193058000
8	-3.113783000	-3.018800000	-0.384843000
1	-4.660028000	4.247978000	0.213595000
1	-5.703068000	3.445356000	-0.991126000
1	-5.891580000	3.072898000	0.743741000
1	-2.882381000	2.861113000	1.209395000
1	-4.138170000	1.736669000	1.770252000
1	-1.891836000	1.081133000	-0.209911000
1	-1.907779000	0.627635000	1.496589000
1	-3.966072000	-0.773694000	1.110958000
1	-3.932798000	-0.329154000	-0.605464000

3e

Solvent=Methanol

7	3.736287000	0.294939000	0.038820000
6	3.447949000	1.610724000	-0.028083000
6	4.446321000	2.565130000	-0.000012000
6	5.766025000	2.123647000	0.102006000
6	6.039936000	0.752178000	0.171380000
6	4.998810000	-0.162467000	0.139213000

6	1.962826000	1.764085000	-0.143115000
8	1.366041000	2.826318000	-0.252646000
7	1.476794000	0.500389000	-0.105324000
6	2.483948000	-0.544134000	0.001945000
6	2.363321000	-1.347048000	1.311709000
6	2.494961000	-1.471601000	-1.227041000
6	1.110688000	-2.234949000	1.291348000
6	1.237423000	-2.355774000	-1.244038000
6	1.085837000	-3.152528000	0.059720000
1	4.178446000	3.612757000	-0.057306000
1	6.579897000	2.839266000	0.127415000
1	7.056065000	0.387149000	0.249474000
1	5.143724000	-1.233058000	0.188384000
1	0.470097000	0.302923000	-0.296404000
1	3.256592000	-1.974333000	1.413127000
1	2.345844000	-0.648040000	2.153123000
1	2.568832000	-0.859082000	-2.130534000
1	3.388610000	-2.104240000	-1.174077000
1	1.094889000	-2.830745000	2.210140000
1	0.214885000	-1.606288000	1.314206000
1	0.349368000	-1.734521000	-1.392605000
1	1.301649000	-3.033437000	-2.101686000
1	1.899350000	-3.887146000	0.137118000
1	0.143670000	-3.707976000	0.036326000
6	-7.313033000	2.273047000	0.098798000
8	-6.146175000	1.523208000	0.387231000
6	-6.213216000	0.192840000	-0.108780000
6	-4.897829000	-0.506701000	0.205317000
6	-3.712573000	0.170243000	-0.478897000
16	-2.134831000	-0.620061000	-0.067196000
8	-1.949192000	-0.461511000	1.404608000
8	-2.239905000	-2.036572000	-0.517706000
8	-1.117534000	0.170423000	-0.860717000
1	-8.210121000	1.822234000	0.550741000
1	-7.171396000	3.272606000	0.517102000
1	-7.483296000	2.361819000	-0.985331000
1	-7.051888000	-0.349023000	0.358413000
1	-6.394172000	0.199457000	-1.197370000
1	-4.739304000	-0.506541000	1.288813000
1	-4.961756000	-1.550745000	-0.117787000
1	-3.800854000	0.129383000	-1.568731000
1	-3.627645000	1.216171000	-0.176851000

3f
Solvent=Ethanol

7	3.980371000	-0.451073000	-0.155228000
6	4.231769000	0.869948000	-0.060050000
6	5.520751000	1.359736000	-0.148046000
6	6.553662000	0.440757000	-0.338947000
6	6.265197000	-0.926241000	-0.434886000
6	4.952538000	-1.362490000	-0.339824000
6	2.930937000	1.588435000	0.144286000

8	2.801833000	2.797963000	0.275586000
7	1.994900000	0.612578000	0.146602000
1	5.688982000	2.426362000	-0.066987000
1	7.579849000	0.782252000	-0.413128000
1	7.051402000	-1.655529000	-0.582857000
1	4.659582000	-2.401765000	-0.405576000
1	0.974142000	0.772788000	0.287949000
6	-7.313292000	0.357798000	-0.221531000
8	-5.915424000	0.175717000	-0.410866000
6	-5.165352000	1.370280000	-0.241704000
6	-3.688218000	1.038346000	-0.407303000
6	-3.207630000	0.044344000	0.647371000
16	-1.475706000	-0.430099000	0.425391000
8	-1.371685000	-1.034748000	-0.935870000
8	-0.705099000	0.865178000	0.540926000
8	-1.163685000	-1.380636000	1.532373000
1	-7.699759000	1.103271000	-0.936305000
1	-7.511734000	0.747817000	0.790598000
1	-5.469344000	2.124681000	-0.985636000
1	-5.352185000	1.801969000	0.756459000
1	-3.523334000	0.615328000	-1.403594000
1	-3.102146000	1.960279000	-0.339223000
1	-3.285311000	0.458094000	1.657194000
1	-3.787646000	-0.880297000	0.606586000
6	2.500380000	-0.739235000	-0.031629000
6	2.240655000	-1.608518000	1.200707000
1	1.159330000	-1.698784000	1.337576000
1	2.663203000	-2.607164000	1.065016000
1	2.675620000	-1.145793000	2.089359000
6	1.986698000	-1.369917000	-1.327875000
1	0.896836000	-1.437085000	-1.268625000
1	2.259622000	-0.749271000	-2.184337000
1	2.397518000	-2.374176000	-1.458528000
6	-7.998906000	-0.982823000	-0.422337000
1	-9.079498000	-0.884281000	-0.282266000
1	-7.812536000	-1.361204000	-1.431933000
1	-7.620852000	-1.716749000	0.295805000

3g
Solvent=2-Propanol

7	4.327810000	-0.362827000	-0.159213000
6	4.532566000	0.949078000	0.073006000
6	5.806300000	1.484676000	0.069300000
6	6.873681000	0.621678000	-0.182643000
6	6.633674000	-0.737372000	-0.420618000
6	5.334387000	-1.221012000	-0.404862000
6	3.203662000	1.604170000	0.308933000
8	3.031585000	2.791590000	0.547890000
7	2.301516000	0.603356000	0.195921000
1	5.936430000	2.542700000	0.259671000
1	7.889250000	1.000718000	-0.194172000

1	7.447246000	-1.423800000	-0.617719000
1	5.077718000	-2.257520000	-0.578201000
1	1.269549000	0.721441000	0.297307000
6	-6.946170000	-0.262936000	-0.246785000
8	-5.567389000	-0.185739000	-0.644873000
6	-4.896695000	1.049300000	-0.413174000
6	-3.400523000	0.796408000	-0.558691000
6	-2.886169000	-0.178985000	0.496931000
16	-1.133666000	-0.577535000	0.292140000
8	-0.988775000	-1.169055000	-1.071159000
8	-0.420687000	0.749131000	0.423507000
8	-0.792490000	-1.520382000	1.397262000
1	-7.264378000	-1.230670000	-0.653042000
1	-5.217195000	1.810498000	-1.139185000
1	-5.111902000	1.442051000	0.591163000
1	-3.200307000	0.388091000	-1.554736000
1	-2.863685000	1.746933000	-0.477432000
1	-2.992397000	0.226437000	1.507597000
1	-3.426199000	-1.126988000	0.445643000
6	2.855754000	-0.707961000	-0.099880000
6	2.603074000	-1.700266000	1.037552000
1	1.523002000	-1.832488000	1.146798000
1	3.057613000	-2.668695000	0.813831000
1	3.010404000	-1.313587000	1.974496000
6	2.387145000	-1.225836000	-1.461337000
1	1.300486000	-1.345040000	-1.426872000
1	2.643448000	-0.511448000	-2.246931000
1	2.841616000	-2.194197000	-1.685348000
6	-7.804466000	0.831470000	-0.884677000
1	-8.864554000	0.628701000	-0.704071000
1	-7.577539000	1.816915000	-0.465095000
1	-7.638107000	0.867841000	-1.965517000
6	-7.094873000	-0.313415000	1.276451000
1	-6.442957000	-1.085816000	1.694608000
1	-6.840684000	0.644236000	1.741795000
1	-8.129528000	-0.547783000	1.546168000

3i
Solvent=2-Methyl-2-Propanol

7	4.621282000	-0.405661000	-0.130383000
6	4.845629000	0.917864000	-0.011871000
6	6.127244000	1.432336000	-0.065007000
6	7.180828000	0.535299000	-0.245349000
6	6.920075000	-0.835642000	-0.365413000
6	5.614020000	-1.296685000	-0.305140000
6	3.527228000	1.609753000	0.176897000
8	3.376391000	2.814359000	0.325989000
7	2.609657000	0.617148000	0.143703000
1	6.273137000	2.500749000	0.034378000
1	8.201874000	0.896653000	-0.292215000
1	7.722867000	-1.548433000	-0.505017000

1	5.341112000	-2.340183000	-0.389176000
1	1.582301000	0.751245000	0.278899000
6	-6.635379000	-0.019490000	-0.124510000
8	-5.259975000	0.064306000	-0.560011000
6	-4.544271000	1.264257000	-0.292075000
6	-3.059724000	0.963397000	-0.466866000
6	-2.575952000	-0.082827000	0.533648000
16	-0.834240000	-0.517576000	0.313231000
8	-0.692472000	-1.022651000	-1.084470000
8	-0.086085000	0.777548000	0.536269000
8	-0.530157000	-1.539243000	1.357510000
1	-4.846146000	2.063597000	-0.984419000
1	-4.730978000	1.626831000	0.729061000
1	-2.882696000	0.598082000	-1.483685000
1	-2.486359000	1.887759000	-0.343261000
1	-2.674162000	0.268768000	1.565149000
1	-3.142943000	-1.010322000	0.428727000
6	3.143563000	-0.721775000	-0.044956000
6	2.872785000	-1.616768000	1.166037000
1	1.790525000	-1.730524000	1.275627000
1	3.317343000	-2.604765000	1.022531000
1	3.278081000	-1.161395000	2.072413000
6	2.672619000	-1.339340000	-1.363782000
1	1.583009000	-1.426883000	-1.333165000
1	2.953266000	-0.698039000	-2.202512000
1	3.105556000	-2.333388000	-1.502337000
6	-7.132375000	-1.320469000	-0.760399000
1	-8.171305000	-1.517531000	-0.480442000
1	-7.068567000	-1.256876000	-1.850679000
1	-6.515535000	-2.160898000	-0.428114000
6	-7.453264000	1.173901000	-0.637526000
1	-7.340097000	1.273078000	-1.721608000
1	-8.514000000	1.028459000	-0.412046000
1	-7.139063000	2.111140000	-0.168774000
6	-6.702620000	-0.118556000	1.407133000
1	-7.733040000	-0.292215000	1.732391000
1	-6.082252000	-0.949411000	1.756146000
1	-6.354715000	0.799221000	1.889959000

3j
Solvent=1-Hexanol

7	5.316663000	-0.324251000	-0.079718000
6	5.488251000	0.980216000	0.212342000
6	6.751745000	1.534871000	0.288482000
6	7.843972000	0.698889000	0.053126000
6	7.638216000	-0.653787000	-0.246341000
6	6.347944000	-1.157190000	-0.309123000
6	4.138930000	1.605376000	0.413123000
8	3.938743000	2.778950000	0.693788000
7	3.259763000	0.597068000	0.216623000
1	6.854322000	2.586558000	0.525357000

1	8.852523000	1.093466000	0.102192000
1	8.471732000	-1.319636000	-0.430584000
1	6.116979000	-2.190279000	-0.531645000
1	2.221502000	0.690481000	0.289060000
8	-4.569287000	-0.159471000	-0.938417000
6	-3.886311000	1.045830000	-0.604593000
6	-2.390615000	0.783639000	-0.735180000
6	-1.914511000	-0.280103000	0.250457000
16	-0.150344000	-0.646153000	0.090041000
8	0.067414000	-1.097112000	-1.316371000
8	0.538301000	0.667027000	0.386697000
8	0.148840000	-1.691450000	1.112002000
1	-4.181960000	1.861104000	-1.280733000
1	-4.118827000	1.368742000	0.420258000
1	-2.168524000	0.454582000	-1.755521000
1	-1.844878000	1.717043000	-0.563345000
1	-2.067419000	0.033316000	1.287588000
1	-2.443417000	-1.222382000	0.092408000
6	3.848545000	-0.692276000	-0.106895000
6	3.556975000	-1.736513000	0.973228000
1	2.474846000	-1.886071000	1.025482000
1	4.033777000	-2.689282000	0.729022000
1	3.914985000	-1.387264000	1.944378000
6	3.455771000	-1.157308000	-1.511379000
1	2.370445000	-1.291010000	-1.537022000
1	3.739164000	-0.406604000	-2.252737000
1	3.935519000	-2.109070000	-1.753811000
6	-5.936826000	-0.273248000	-0.523822000
6	-6.821170000	0.889132000	-0.993759000
6	-8.290800000	0.639201000	-0.616508000
6	-8.444609000	0.402195000	0.893713000
6	-7.544688000	-0.748448000	1.369599000
6	-6.075527000	-0.502145000	0.988330000
1	-6.279863000	-1.184593000	-1.032626000
1	-6.708337000	1.018396000	-2.076323000
1	-6.485017000	1.820161000	-0.519139000
1	-8.908687000	1.485635000	-0.937228000
1	-8.659074000	-0.242536000	-1.159426000
1	-9.491973000	0.192860000	1.140686000
1	-8.169884000	1.320549000	1.431673000
1	-7.634021000	-0.883172000	2.453548000
1	-7.885061000	-1.685799000	0.907413000
1	-5.444435000	-1.348509000	1.281171000
1	-5.707725000	0.378500000	1.530514000

3h

Solvent=2-Methyl-1-Propanol

7	-3.866202000	-0.651519000	-0.584823000
6	-4.061834000	-0.841522000	0.723745000
6	-5.118208000	-1.583650000	1.187840000
6	-5.975819000	-2.132331000	0.244397000
6	-5.750523000	-1.922202000	-1.111582000
6	-4.670844000	-1.166744000	-1.516373000

6	-2.979144000	-0.116872000	1.466260000
8	-2.872909000	-0.065472000	2.674299000
7	-2.211577000	0.429831000	0.514787000
1	-5.252014000	-1.718450000	2.249017000
1	-6.820558000	-2.723566000	0.561519000
1	-6.407005000	-2.340030000	-1.856544000
1	-4.436619000	-0.963973000	-2.548572000
1	-1.441239000	1.090918000	0.720359000
6	4.993858000	-1.750135000	0.439200000
8	3.869763000	-0.964811000	0.077901000
6	2.838257000	-1.027445000	1.046674000
6	1.711591000	-0.113503000	0.611734000
6	2.181302000	1.328891000	0.528466000
16	0.854327000	2.434637000	0.038795000
8	0.380657000	1.974140000	-1.303204000
8	-0.227347000	2.260591000	1.078397000
8	1.419965000	3.810236000	0.036794000
1	4.692208000	-2.798130000	0.538299000
1	2.476276000	-2.055181000	1.138827000
1	3.228614000	-0.714291000	2.020473000
1	1.342562000	-0.438664000	-0.359842000
1	0.896176000	-0.196191000	1.329802000
1	2.529670000	1.696709000	1.491734000
1	2.965642000	1.454754000	-0.212885000
6	-2.642867000	0.180482000	-0.847651000
6	-3.022390000	1.468099000	-1.556192000
1	-2.119408000	2.069917000	-1.633041000
1	-3.390480000	1.260727000	-2.558398000
1	-3.776743000	2.006320000	-0.987772000
6	-1.628421000	-0.641364000	-1.624215000
1	-0.728873000	-0.037873000	-1.718273000
1	-1.402408000	-1.562770000	-1.092470000
1	-2.003840000	-0.870692000	-2.619192000
1	5.380337000	-1.413325000	1.408891000
6	6.073523000	-1.609417000	-0.616238000
1	5.645977000	-1.926999000	-1.569471000
6	6.533801000	-0.160540000	-0.733497000
1	7.302848000	-0.062253000	-1.498719000
1	5.704853000	0.495783000	-0.988226000
1	6.956138000	0.173894000	0.216178000
6	7.242174000	-2.526154000	-0.266931000
1	6.926176000	-3.566622000	-0.197937000
1	8.022470000	-2.456331000	-1.022982000
1	7.676676000	-2.237106000	0.691426000

Product id	Structure
**2a**	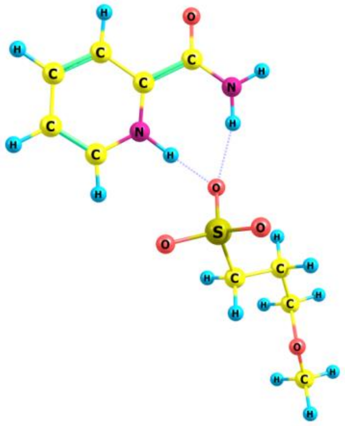
**2b**	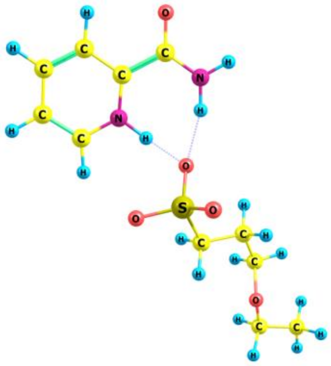
**2c**	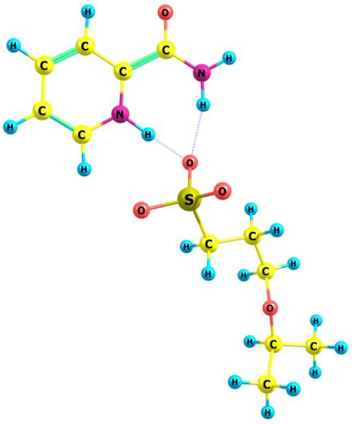
**2d**	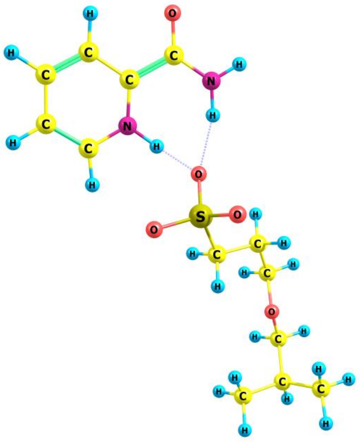

**2e**	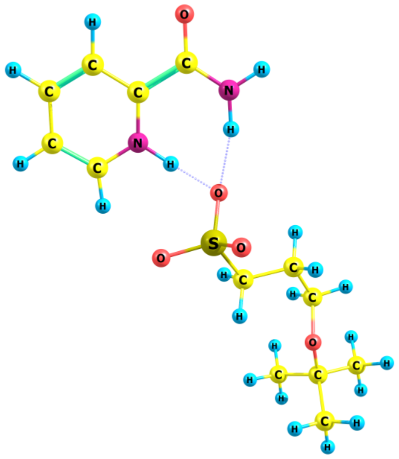
**2f**	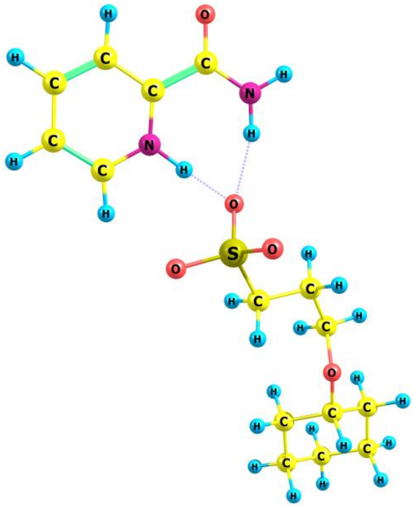
**2g**	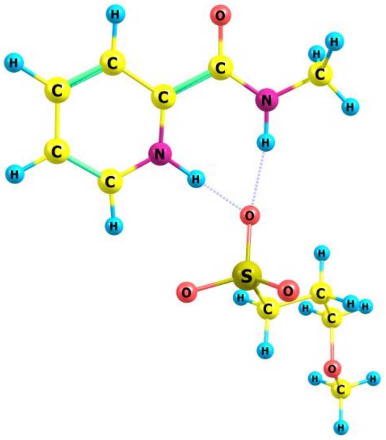
**3a**	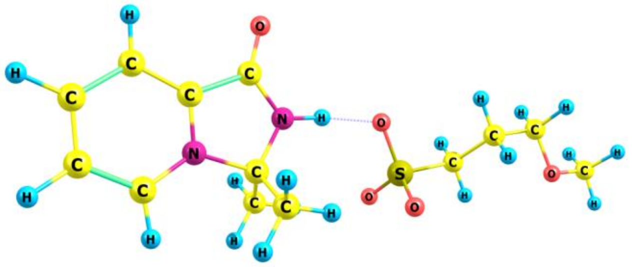

**3b**	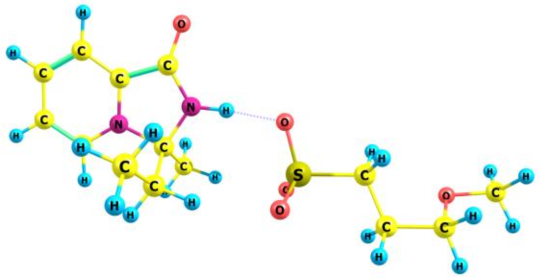
**3c**	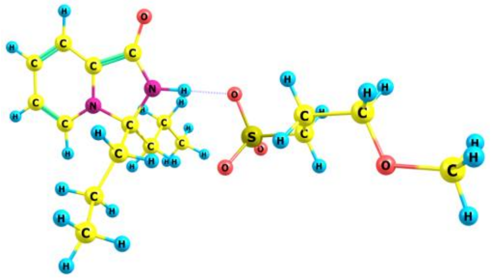
**3d**	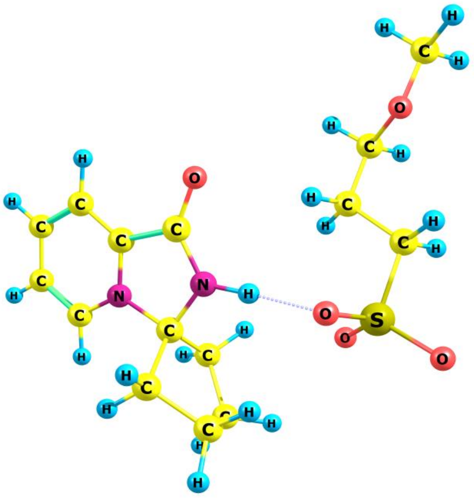
**3e**	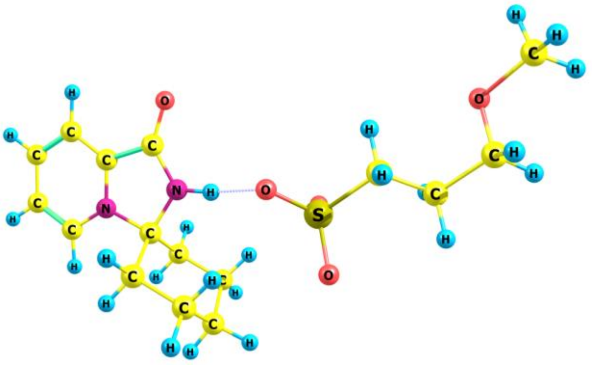
**3f**	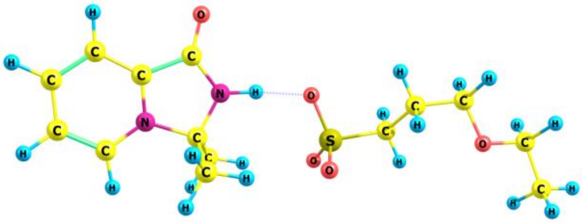
**3g**	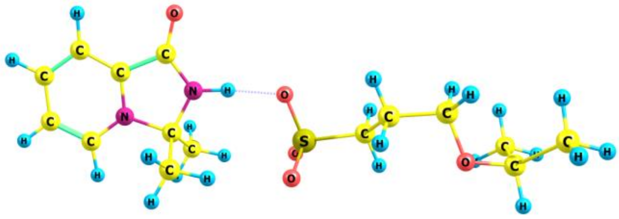

**3h**	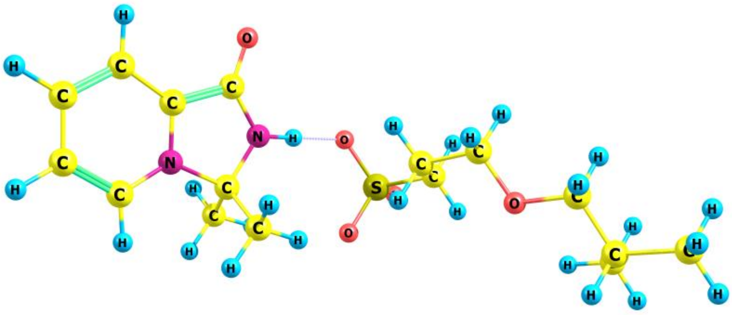
**3i**	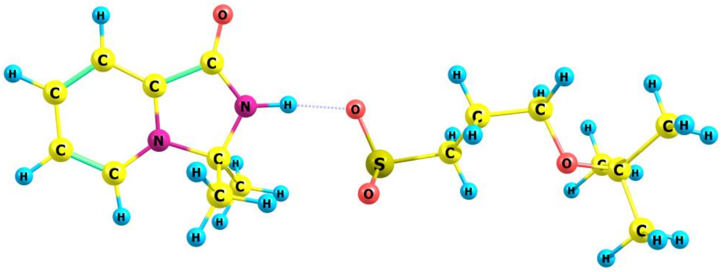
**3j**	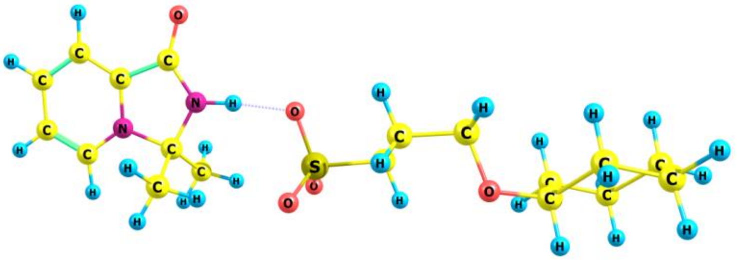

Table S4. The enthalpy ($\Delta_rH°$) and Gibbs free energy ($\Delta_rG°$) of the formation reaction of salt 3a-3k

Reagent id	Reagent structure	Product id	Product structure	Yield, %	ΔH, kJ/mol	ΔG, kJ/mol
**2a**	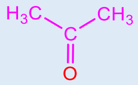	**3a**	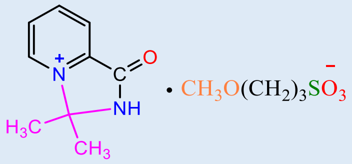	80	-23.5	-5.1
**2a**	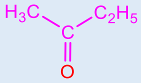	**3b**	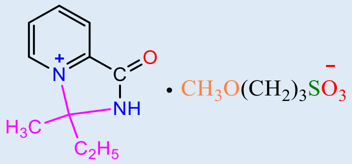	91	-21.1	-4.7
**2a**	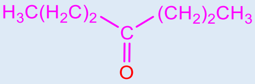	**3c**	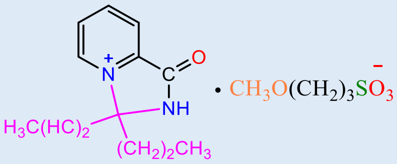	75	-14.9	5.5
**2a**	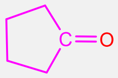	**3d**	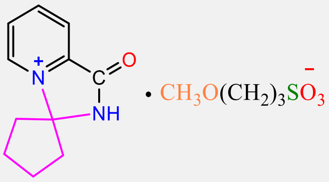	85	-5.2	11.9
**2a**	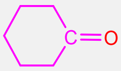	**3e**	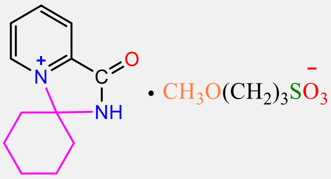	94	-18.8	-0.9
**2b**	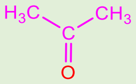	**3f**	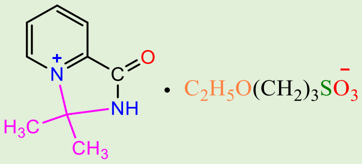	82	-18.6	-8.1
**2c**	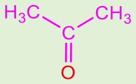	**3g**	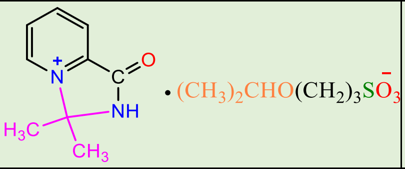	43	-5.8	11.0
**2d**	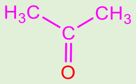	**3h**	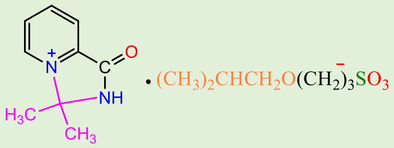	73	-12.8	4.5
**2e**	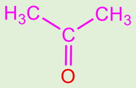	**3i**	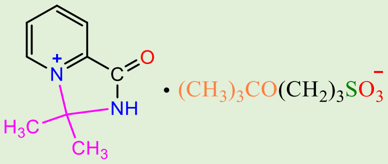	23	-9.1	10.1

**2f**	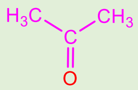	**3j**	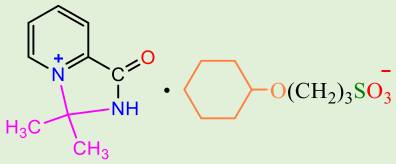	80	-8.9	8.9

Table S5. ^{13}C NMR chemical shifts (theoretical calculations).

TMS = 189.594

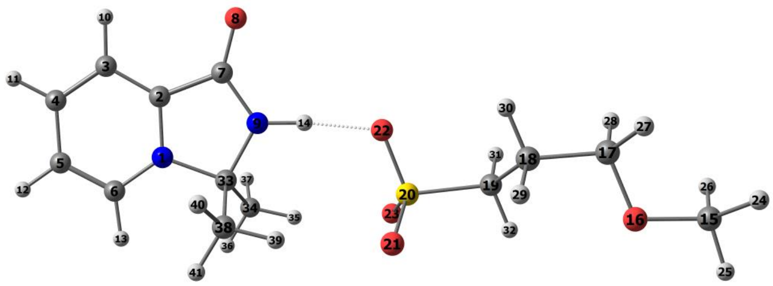

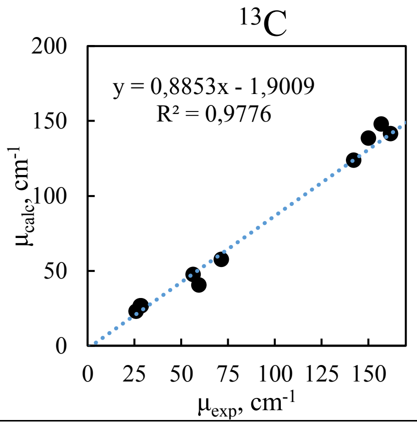

		**Product 3a**			
№	atom	μ_{abs}, cm^{-1}	μ_{rev}, cm^{-1}	μ_{exp}, cm^{-1}	$\mu_{exp-rev}$, cm^{-1}
1	N	14.2629	208.56		
2	C	48.8184	161.7246	141.63	-2.1726
3	C	72.946	142.1563	123.80	4.125
4	C	52.8466	172.3476	130.93	-8.8444
5	C	71.1732	150.0593	138.48	17.0322
6	C	60.1224	156.8397	148.03	15.5314
7	C	45.742	174.2835	158.99	12.111
8	O	-21.1556	218.2502		
9	N	107.3542	100.4219		
10	H	23.4229	166.9827		
11	H	23.3894	167.5866		
12	H	23.9459	166.9999		
13	H	23.0897	167.4057		
14	H	17.8586	169.03		
15	C	134.1743	59.4917	40.57	-17.8767
16	O	301.7729	-111.035		
17	C	120.2766	71.432	57.69	-14.6544
18	C	163.5314	25.9159	22.91	-6.1796
19	C	138.1012	56.3756	47.65	-6.8698
20	S	209.4348	50.3134		
21	O	117.0187	84.0678		
22	O	117.5693	95.4941		
23	O	106.8506	103.0875		
24	H	28.6959	162.0775		
25	H	28.2007	162.2289		
26	H	28.7393	162.0157		
27	H	28.3787	162.3116		
28	H	28.7361	162.0179		
29	H	29.5535	160.9436		
30	H	30.0421	160.8129		

31	H	29.5867	161.4196		
32	H	28.887	161.8352		
33	C	102.2389	90.0779		
34	C	163.5571	28.6651	26.53	
35	H	26.9261	161.5717		
36	H	30.2939	160.5588		
37	H	30.8508	159.8867		
38	C	163.5755	28.027	26.53	
39	H	26.9128	161.6211		
40	H	30.8616	159.8739		
41	H	30.3037	160.5459		

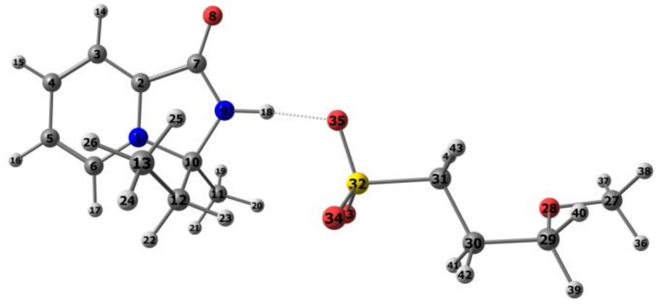

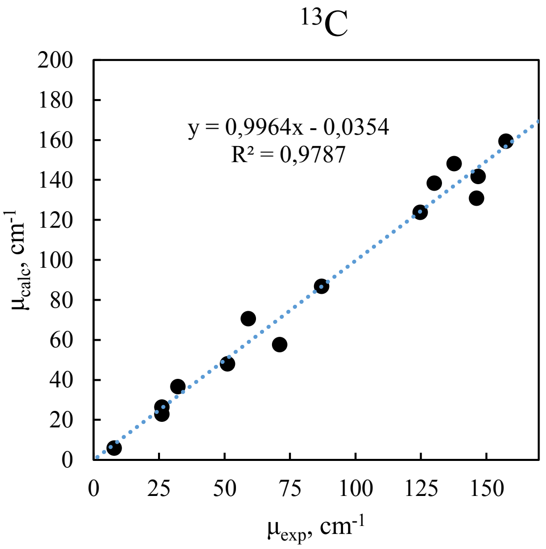

Product 3b

№	atom	μ_{abs}, cm^{-1}	μ_{rev}, cm^{-1}	μ_{exp}, cm^{-1}	$\mu_{exp-rev}$, cm^{-1}
1	N	28.5962	164.0248		
2	C	45.8148	146.8062	141.79	-5.0162
3	C	67.8816	124.7394	123.94	-0.7994
4	C	46.4289	146.1921	131.01	-15.1821
5	C	62.5648	130.0562	138.54	8.4838
6	C	54.9268	137.6942	148.27	10.5758
7	C	35.1659	157.4551	159.44	1.9849
8	O	12.0356	180.5854		
9	N	115.5449	77.0761		
10	C	105.5781	87.0429	86.95	-0.0929
11	C	166.5885	26.0325	26.43	0.3975
12	C	160.4506	32.1704	36.69	4.5196
13	C	184.7698	7.85120	5.99	-1.8612
14	H	23.1893	169.4317		
15	H	22.626	169.995		
16	H	23.1083	169.5127		
17	H	22.7997	169.8213		
18	H	20.7303	171.8907		
19	H	28.9863	163.6347		
20	H	27.4139	165.2071		
21	H	28.5737	164.0473		
22	H	28.2192	164.4018		

23	H	26.9801	165.6409		
24	H	29.0209	163.6001		
25	H	28.8496	163.7714		
26	H	29.9525	162.6685		
27	C	133.5973	59.0237	70.64	11.6163
28	O	289.2235	-96.6025		
29	C	121.5668	71.0542	57.75	-13.3042
30	C	166.566	26.055	22.97	-3.085
31	C	141.5375	51.0835	48.03	-3.0535
32	S	131.0959	61.5251		
33	O	136.9628	55.6582		
34	O	135.4536	57.1674		
35	O	128.0845	64.5365		
36	H	27.1062	165.5148		
37	H	26.6789	165.9421		
38	H	27.1709	165.4501		
39	H	26.9389	165.6821		
40	H	27.1688	165.4522		
41	H	28.1127	164.5083		
42	H	28.5692	164.0518		
43	H	28.0343	164.5867		
44	H	27.5334	165.0876		

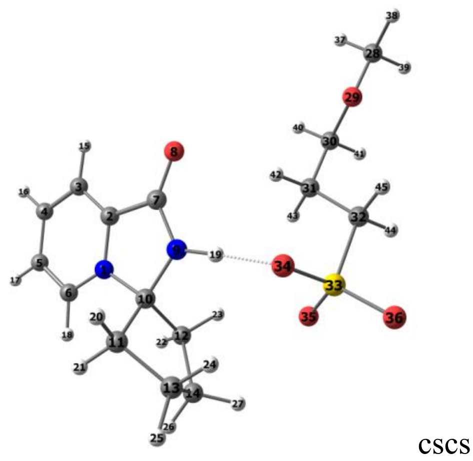

cscs

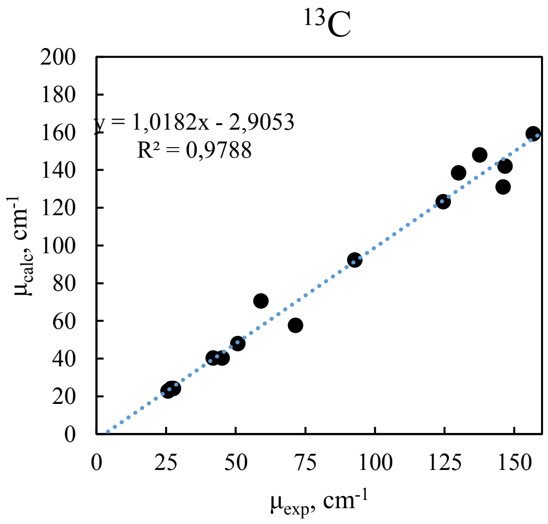

Product 3d

№	atom	μ_{abs}, cm^{-1}	μ_{rev}, cm^{-1}	μ_{exp}, cm^{-1}	$\mu_{exp-rev}$, cm^{-1}
1	N	27.4131	165.2079		
2	C	45.8821	146.7389	142.18	-4.5589
3	C	68.1328	124.4882	123.29	-1.1982
4	C	46.5597	146.0613	131.00	-15.0613
5	C	62.5971	130.0239	138.51	8.4861
6	C	54.9552	137.6658	148.11	10.4442
7	C	35.7486	156.8724	159.20	2.3276

8	O	15.0109	177.6101		
9	N	100.5096	92.1114		
10	C	99.9006	92.7204	92.40	-0.3204
11	C	147.5018	45.1192	40.33	-4.7892
12	C	150.7368	41.8842	40.33	-1.5542
13	C	164.9266	27.6944	24.16	-3.5344
14	C	165.7254	26.8956	24.16	-2.7356
15	H	23.2304	169.3906		
16	H	22.6868	169.9342		
17	H	23.1699	169.4511		
18	H	22.7776	169.8434		
19	H	19.7796	172.8414		
20	H	28.2237	164.3973		
21	H	27.933	164.688		
22	H	28.4473	164.1737		
23	H	27.5697	165.0513		
24	H	27.5616	165.0594		
25	H	28.2529	164.3681		
26	H	28.2238	164.3972		
27	H	28.0138	164.6072		
28	C	133.5814	59.0396	70.64	11.6004
29	O	288.8086	-96.1876		
30	C	121.1887	71.4323	57.75	-13.6823
31	C	166.9234	25.6976	22.80	-2.8976
32	C	141.8817	50.7393	47.93	-2.8093
33	S	130.0256	62.5954		
34	O	127.2674	65.3536		
35	O	141.7586	50.8624		
36	O	139.541	53.08		
37	H	27.1032	165.5178		
38	H	26.6691	165.9519		
39	H	27.1881	165.4329		
40	H	27.0292	165.5918		
41	H	27.224	165.397		
42	H	27.7468	164.8742		
43	H	28.9057	163.7153		
44	H	28.2323	164.3887		
45	H	27.6256	164.9954		

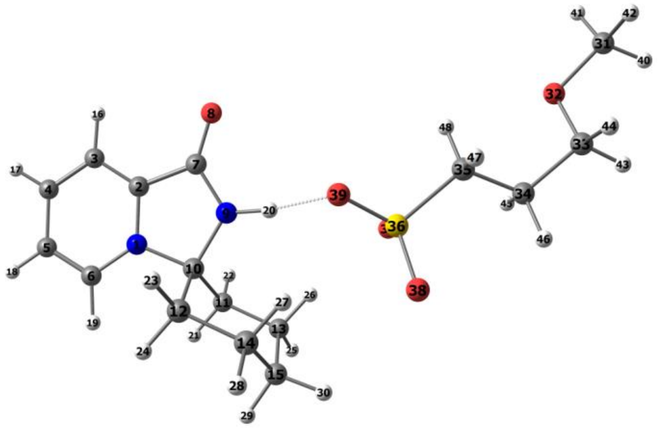

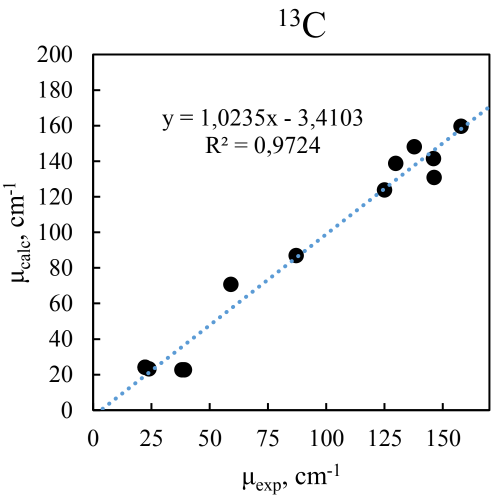

Product 3e

№	atom	μ_{abs}, cm^{-1}	μ_{rev}, cm^{-1}	μ_{exp}, cm^{-1}	$\mu_{exp-rev}$, cm^{-1}
1	N	24.5032	168.1178		
2	C	46.6028	146.0182	141.48	-4.5382
3	C	67.6819	124.9391	123.87	-1.0691
4	C	46.344	146.277	130.84	-15.437
5	C	62.8485	129.7725	138.67	8.8975
6	C	54.8047	137.8163	148.11	10.2937
7	C	34.778	157.843	159.64	1.797
8	O	7.7969	184.8241		
9	N	110.8246	81.7964		
10	C	105.5261	87.0949	86.88	-0.2149
11	C	153.5085	39.1125	22.57	-16.5425
12	C	154.4956	38.1254	22.57	-15.5554
13	C	170.3595	22.2615	24.17	1.9085
14	C	170.3464	22.2746	24.17	1.8954
15	C	168.7324	23.8886	23.17	-0.7186
16	H	23.1938	169.4272		
17	H	22.6525	169.9685		
18	H	23.1644	169.4566		
19	H	22.8084	169.8126		
20	H	19.9459	172.6751		
21	H	28.1228	164.4982		
22	H	28.5236	164.0974		
23	H	28.5355	164.0855		
24	H	28.1012	164.5198		
25	H	28.5067	164.1143		
26	H	27.6723	164.9487		
27	H	27.5891	165.0319		
28	H	28.5275	164.0935		
29	H	28.474	164.147		
30	H	28.2932	164.3278		
31	C	133.5856	59.0354	70.65	11.6146
32	O	289.1872	-96.5662		
33	C	121.5032	71.1178	57.75	-13.3678

34	C	166.469	26.152	22.98	-3.172
35	C	141.2344	51.3866	47.94	-3.4466
36	S	133.8927	58.7283		
37	O	134.6961	57.9249		
38	O	137.0339	55.5871		
39	O	123.4072	69.2138		
40	H	27.0945	165.5265		
41	H	26.6589	165.9621		
42	H	27.1601	165.4609		
43	H	26.9322	165.6888		
44	H	27.1636	165.4574		
45	H	28.1051	164.5159		
46	H	28.5806	164.0404		
47	H	28.0226	164.5984		
48	H	27.4992	165.1218		

Table S6. The characteristics of hydrogen bonds in 2a [Å and °]

D—H···A	D—H (Å)	H···A (Å)	D···A (Å)	D—H···A°
N2A—H2A···O4	0.88	1.95	2.735(5)	147.4
N1B—H1BB[1]···O2A	0.88	2.02	2.881(5)	164.5
N2B—H2B···O4A	0.88	1.92	2.697(5)	146.4
N2—H2···O7B	0.88	1.95	2.730(5)	146.7
N2C—H2C···O7C	0.88	1.92	2.698(5)	146.4
N1C—H1CB[2]···O2C	0.88	2.05	2.883(5)	164.3

The atoms are obtained by symmetry transformations (1) — -1 + x, y, z; (2) — 1 + x, y, z.

Table S7. The characteristics of hydrogen bonds in 3a [Å and °]

D—H···A	D—H (Å)	H···A (Å)	D···A (Å)	D—H···A°
N2—H2[1]···O1	0.88	1.94	2.730(4)	148.8

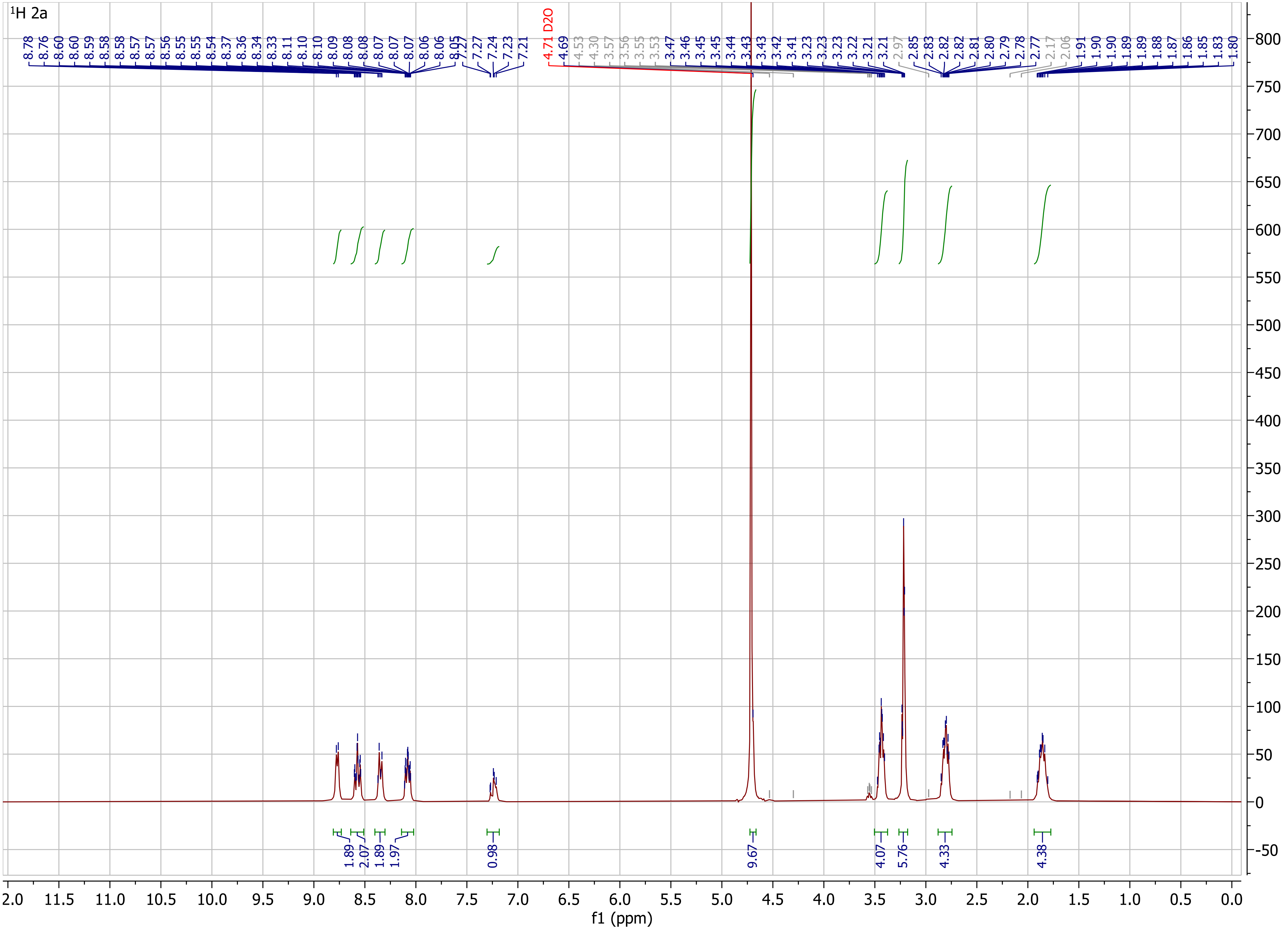
1H 2a
8.78 8.76 8.60 8.60 8.59 8.58 8.58 8.57 8.57 8.56 8.55 8.55 8.54 8.37 8.36 8.34 8.33 8.11 8.10 8.10 8.09 8.08 8.08 8.07 8.07 8.07 8.06 8.06 8.05 7.27 7.27 7.24 7.23 7.21
4.71 D2O
4.69 4.53 4.30 3.57 3.56 3.55 3.53 3.47 3.46 3.45 3.45 3.44 3.43 3.43 3.42 3.41 3.23 3.23 3.23 3.22 3.21 3.21 2.97 2.85 2.83 2.82 2.82 2.81 2.80 2.79 2.78 2.77 2.17 2.06 1.91 1.90 1.90 1.89 1.89 1.88 1.87 1.86 1.85 1.83 1.80
1.89 2.07 1.89 1.97 0.98 9.67 4.07 5.76 4.33 4.38
f1 (ppm)

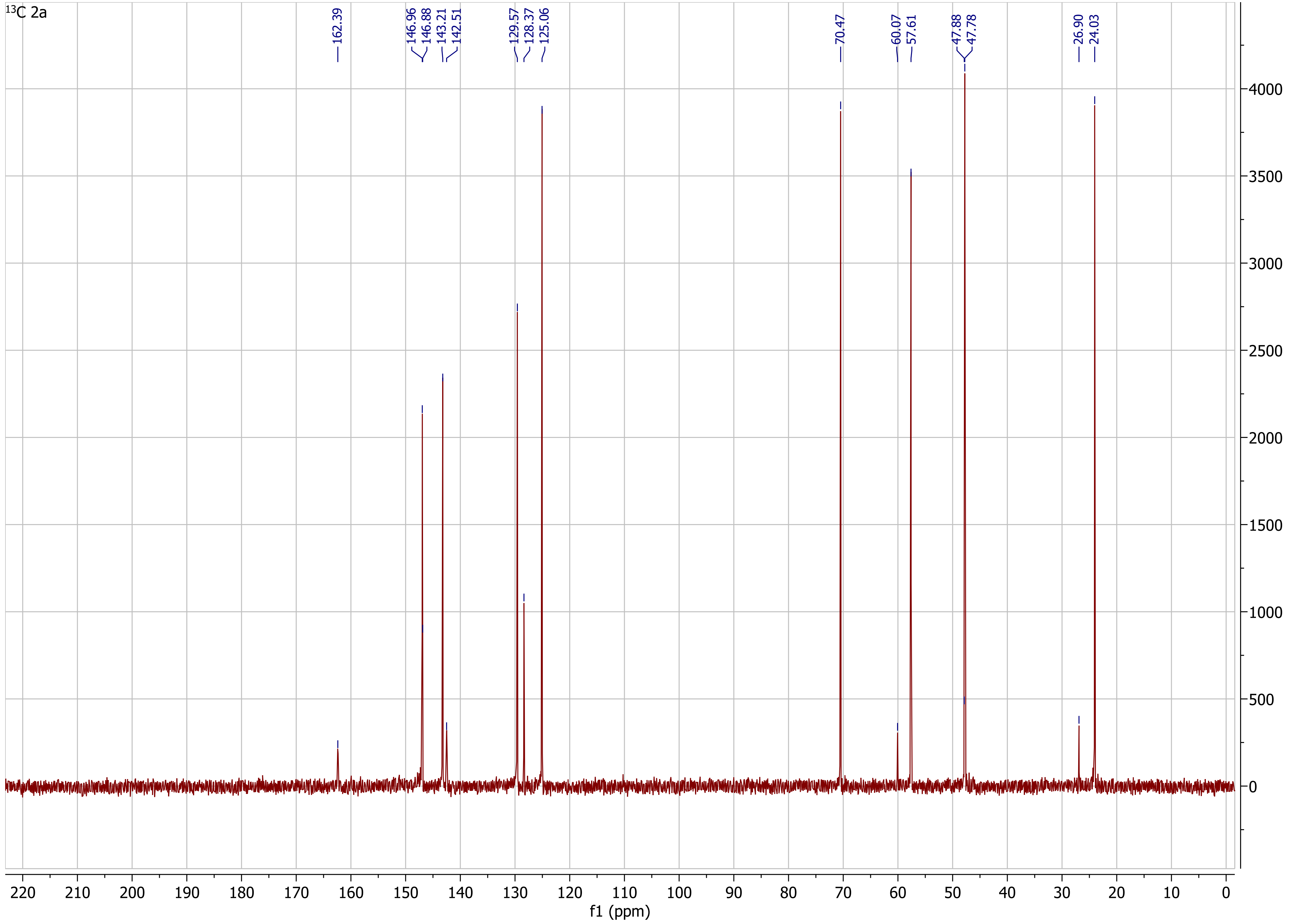

13C 2a
162.39
146.96
146.88
143.21
142.51
129.57
128.37
125.06
70.47
60.07
57.61
47.88
47.78
26.90
24.03
f1 (ppm)

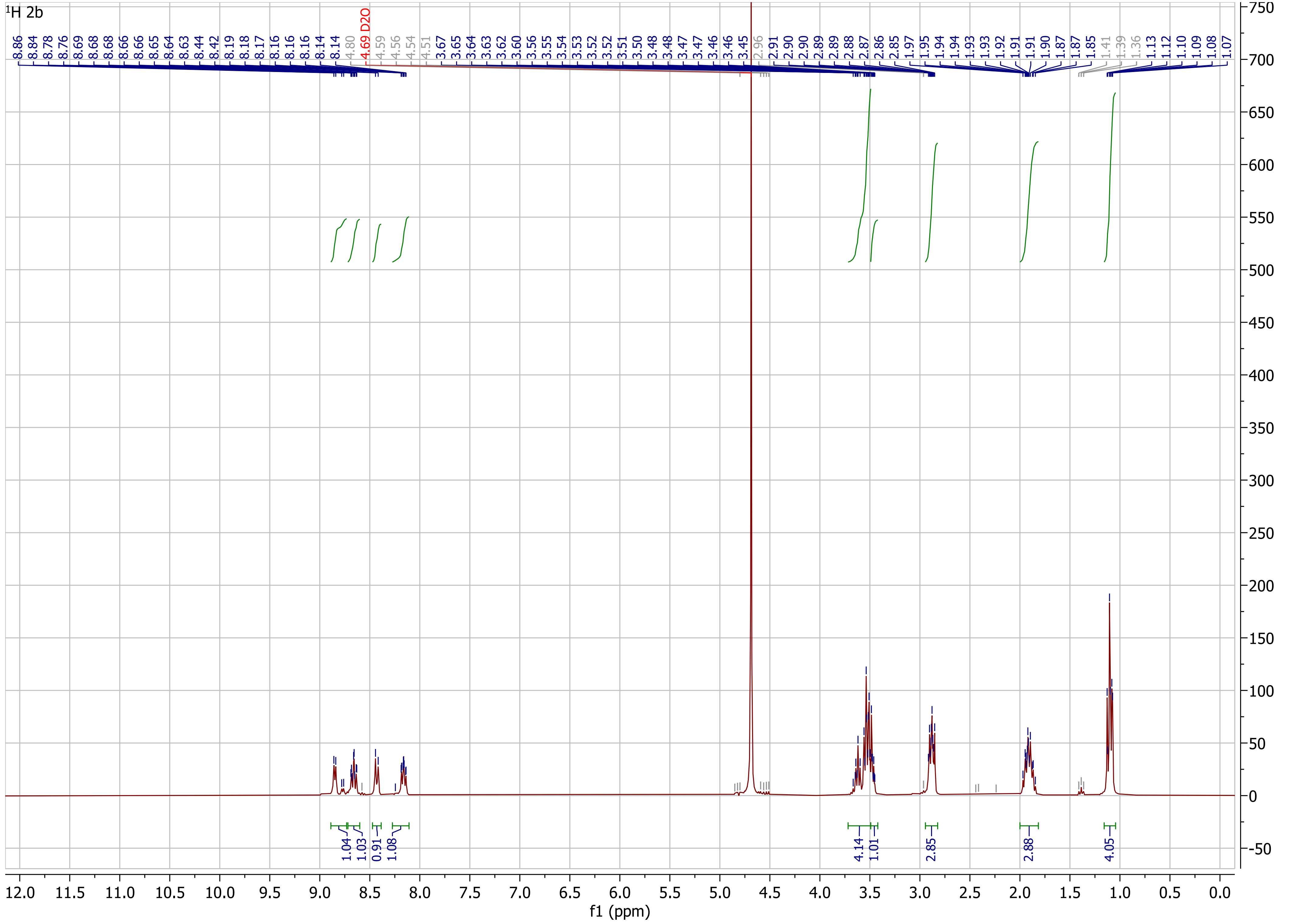

1H 2b
8.86 8.84 8.78 8.76 8.69 8.68 8.68 8.66 8.66 8.65 8.64 8.63 8.44 8.42 8.19 8.18 8.17 8.16 8.16 8.16 8.14 8.14 4.80 4.69 D2O 4.59 4.56 4.54 4.51 3.67 3.65 3.64 3.63 3.62 3.60 3.56 3.55 3.54 3.53 3.52 3.52 3.51 3.50 3.48 3.48 3.47 3.47 3.46 3.46 3.45 2.96 2.91 2.90 2.90 2.89 2.89 2.88 2.87 2.86 2.85 1.97 1.95 1.94 1.94 1.93 1.93 1.92 1.91 1.91 1.91 1.90 1.87 1.87 1.85 1.41 1.39 1.36 1.13 1.12 1.10 1.09 1.08 1.07
1.04 1.03 0.91 1.08 4.14 1.01 2.85 2.88 4.05
f1 (ppm)

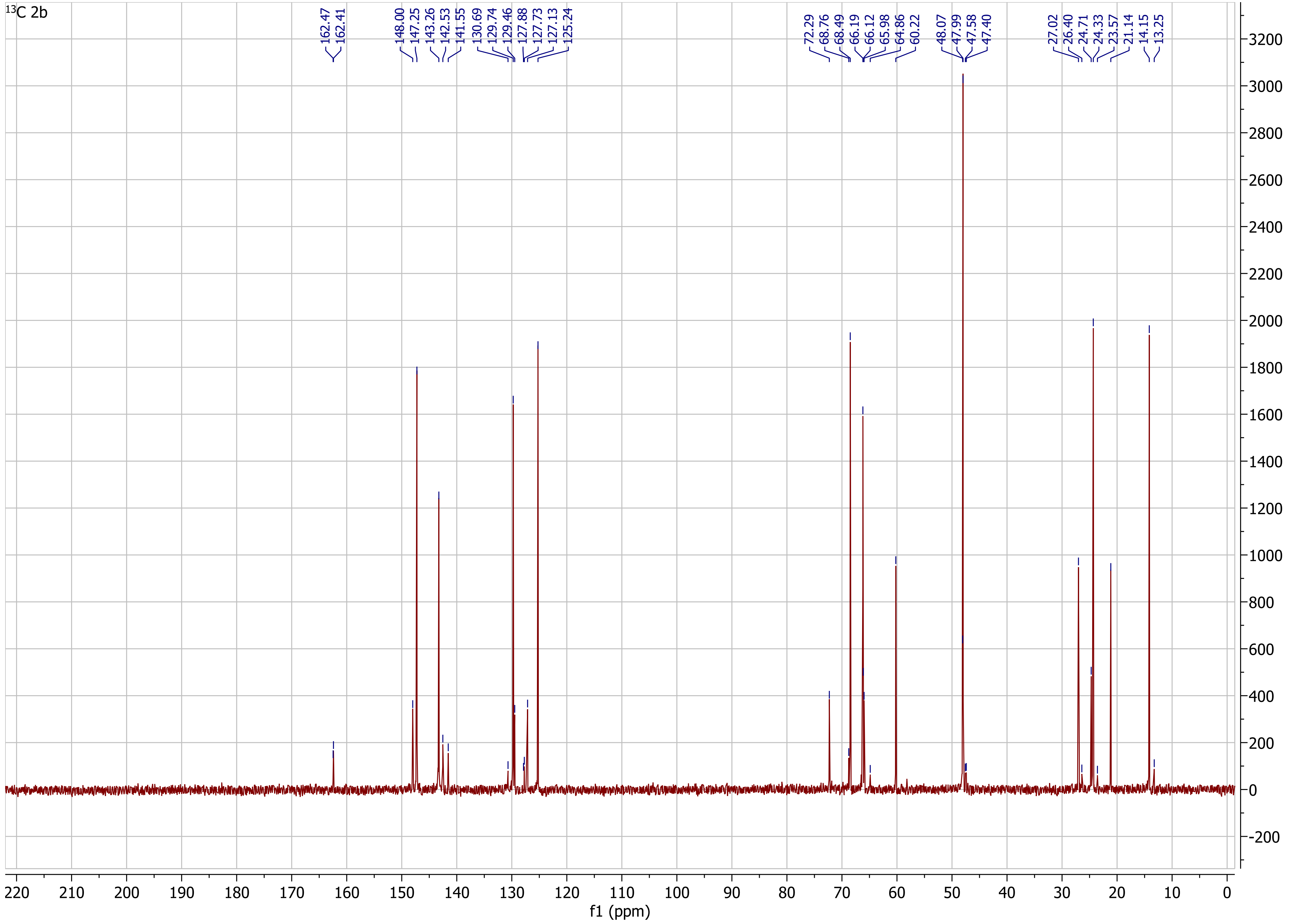
13C 2b
162.47
162.41
148.00
147.25
143.26
142.53
141.55
130.69
129.74
129.46
127.88
127.73
127.13
125.24
72.29
68.76
68.49
66.19
66.12
65.98
64.86
60.22
48.07
47.99
47.58
47.40
27.02
26.40
24.71
24.33
23.57
21.14
14.15
13.25
f1 (ppm)

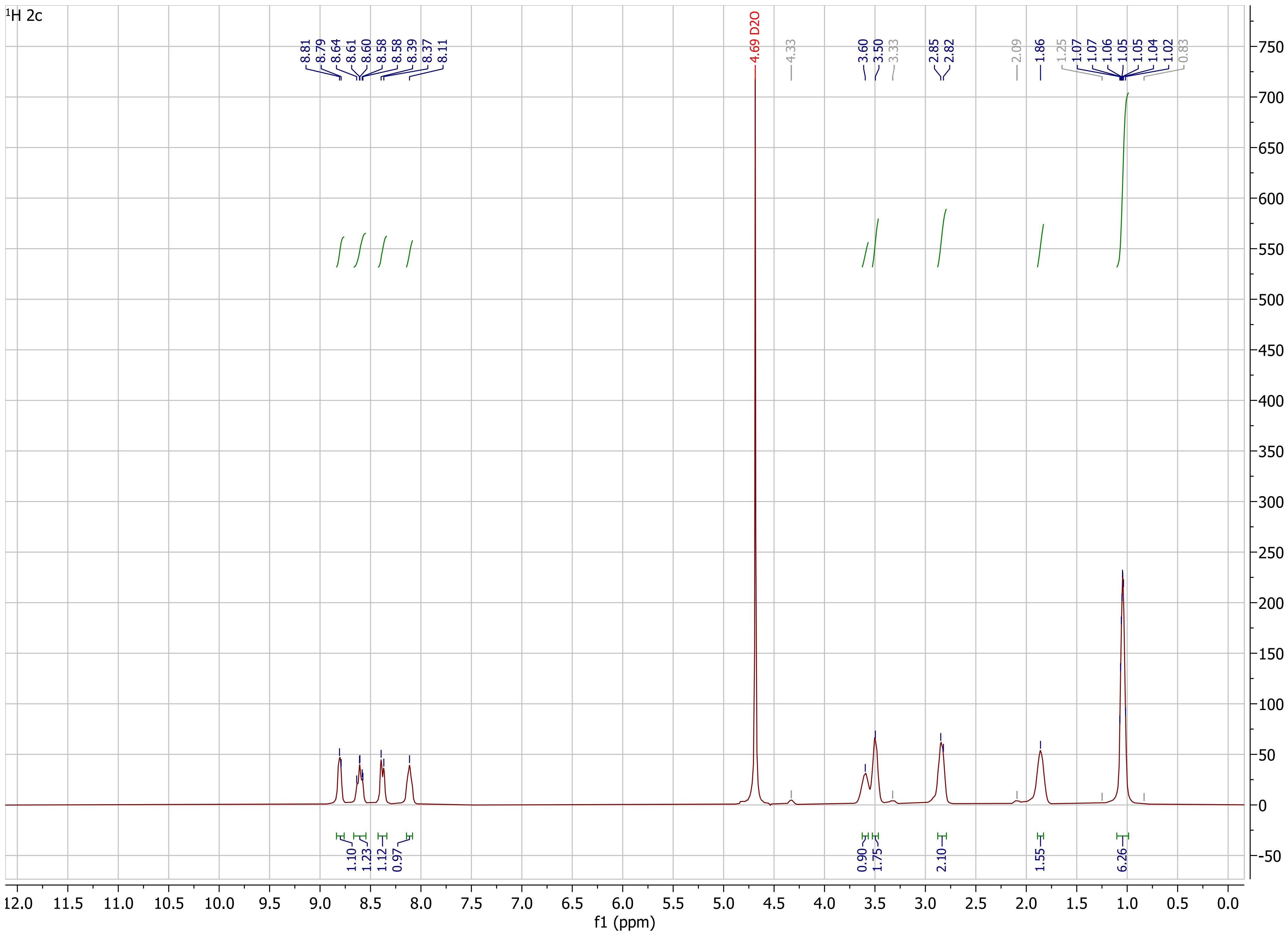

¹H 2c
8.81
8.79
8.64
8.61
8.60
8.58
8.58
8.39
8.37
8.11
4.69 D2O
4.33
3.60
3.50
3.33
2.85
2.82
2.09
1.86
1.25
1.07
1.07
1.06
1.05
1.05
1.04
1.02
0.83
1.10
1.23
1.12
0.97
0.90
1.75
2.10
1.55
6.26
f1 (ppm)

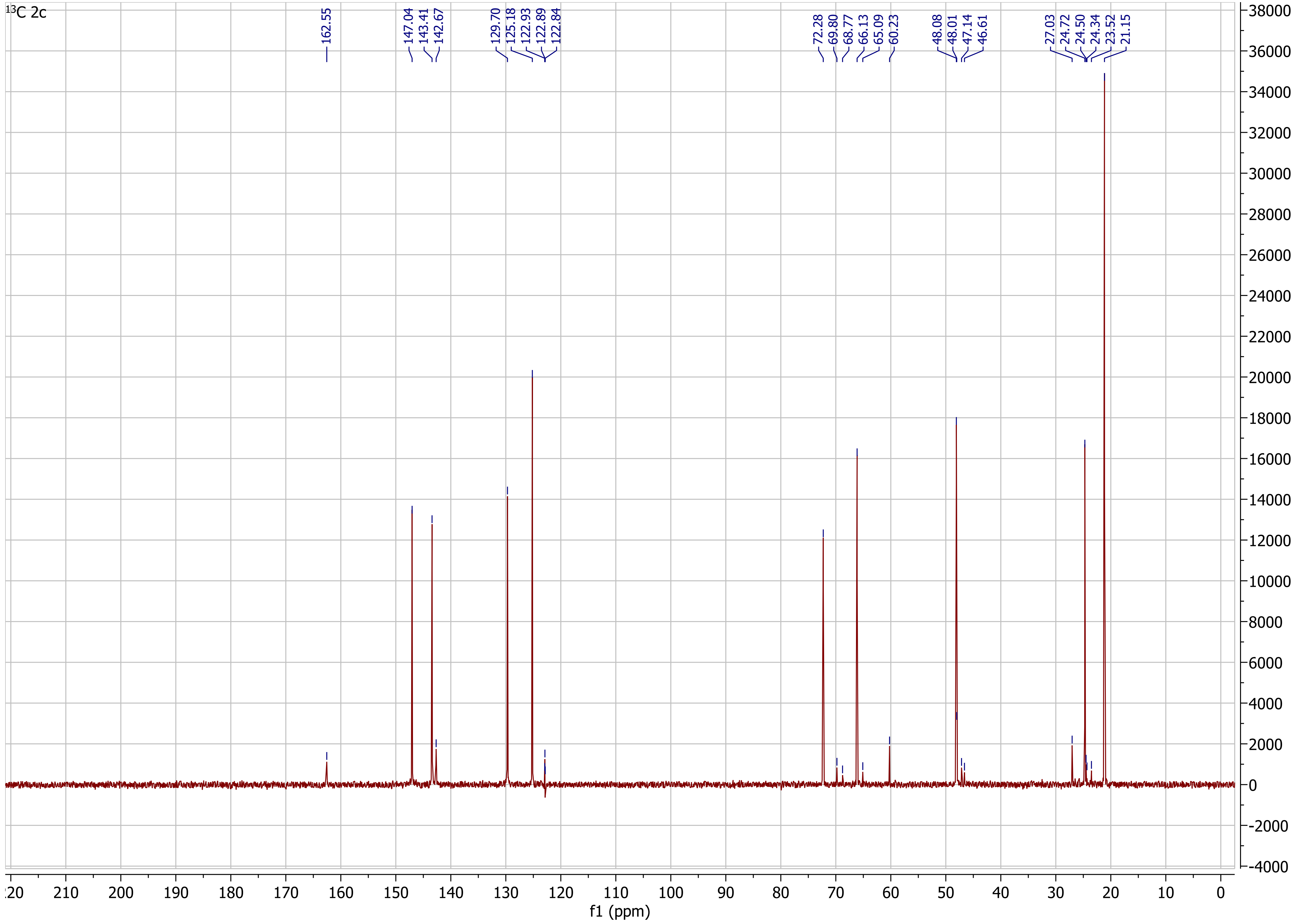
13C 2c
162.55
147.04
143.41
142.67
129.70
125.18
122.93
122.89
122.84
72.28
69.80
68.77
66.13
65.09
60.23
48.08
48.01
47.14
46.61
27.03
24.72
24.50
24.34
23.52
21.15
f1 (ppm)

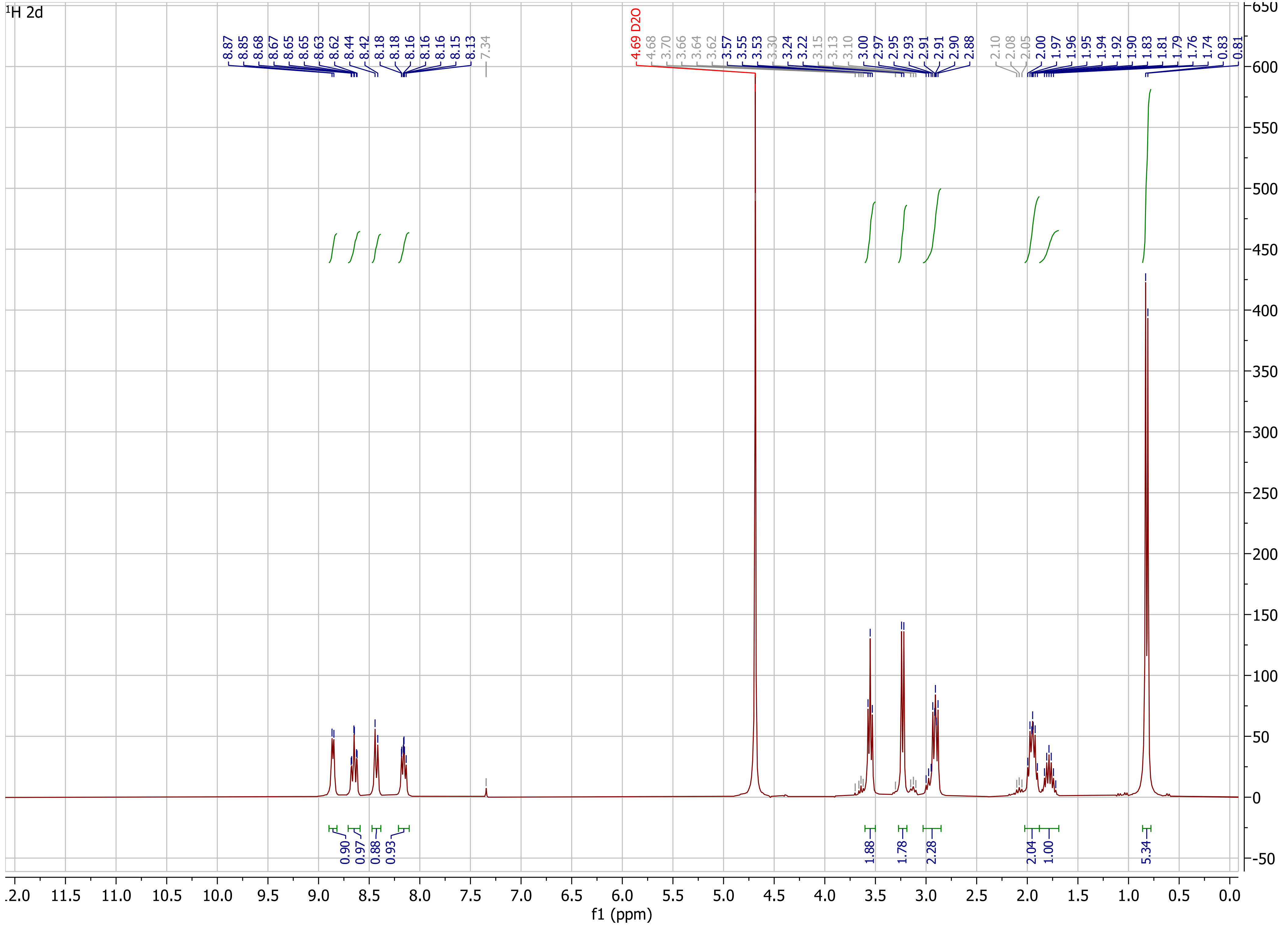

¹H 2d
8.87
8.85
8.68
8.67
8.65
8.65
8.63
8.62
8.44
8.42
8.18
8.18
8.16
8.16
8.16
8.15
8.13
7.34
4.69 D2O
4.68
3.70
3.66
3.64
3.62
3.57
3.55
3.53
3.30
3.24
3.22
3.15
3.13
3.10
3.00
2.97
2.95
2.93
2.91
2.91
2.90
2.88
2.10
2.08
2.05
2.00
1.97
1.96
1.95
1.94
1.92
1.90
1.83
1.81
1.79
1.76
1.74
0.83
0.81
0.90
0.97
0.88
0.93
1.88
1.78
2.28
2.04
1.00
5.34
f1 (ppm)

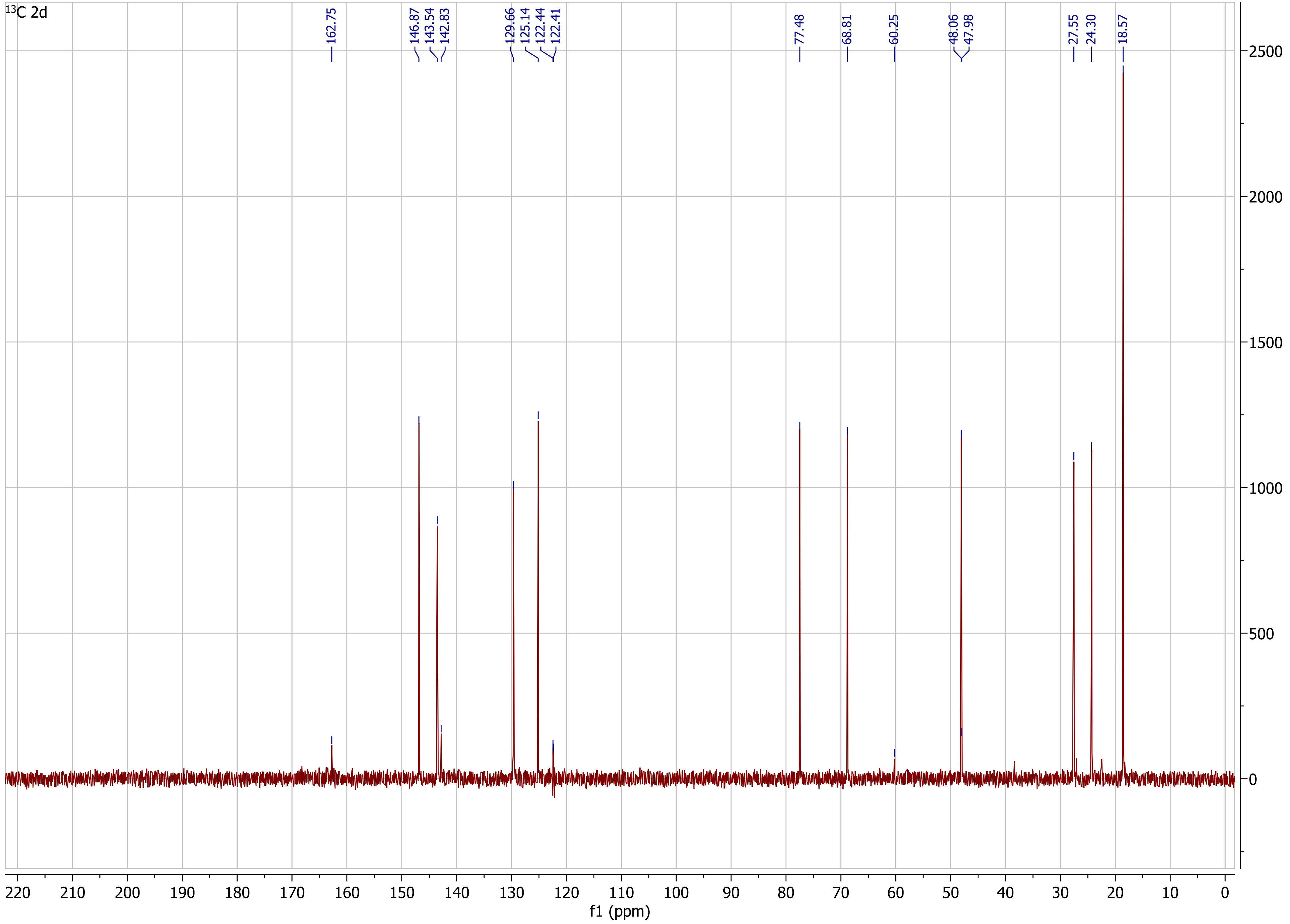

13C 2d
162.75
146.87
143.54
142.83
129.66
125.14
122.44
122.41
77.48
68.81
60.25
48.06
47.98
27.55
24.30
18.57
f1 (ppm)

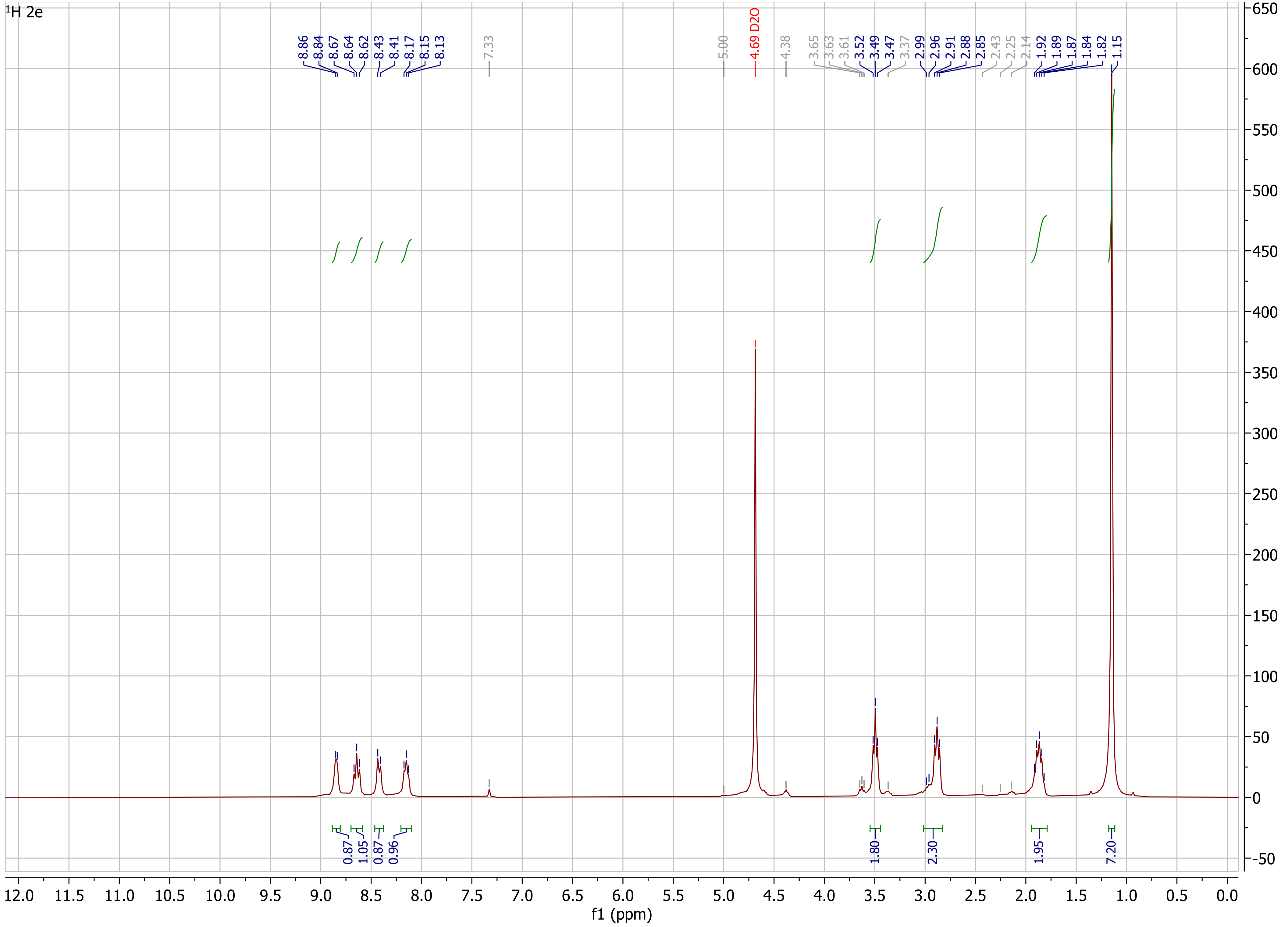

1H 2e
8.86
8.84
8.67
8.64
8.62
8.43
8.41
8.17
8.15
8.13
7.33
5.00
4.69 D2O
4.38
3.65
3.63
3.61
3.52
3.49
3.47
3.37
2.99
2.96
2.91
2.88
2.85
2.43
2.25
2.14
1.92
1.89
1.87
1.84
1.82
1.15
0.87
1.05
0.87
0.96
1.80
2.30
1.95
7.20
f1 (ppm)

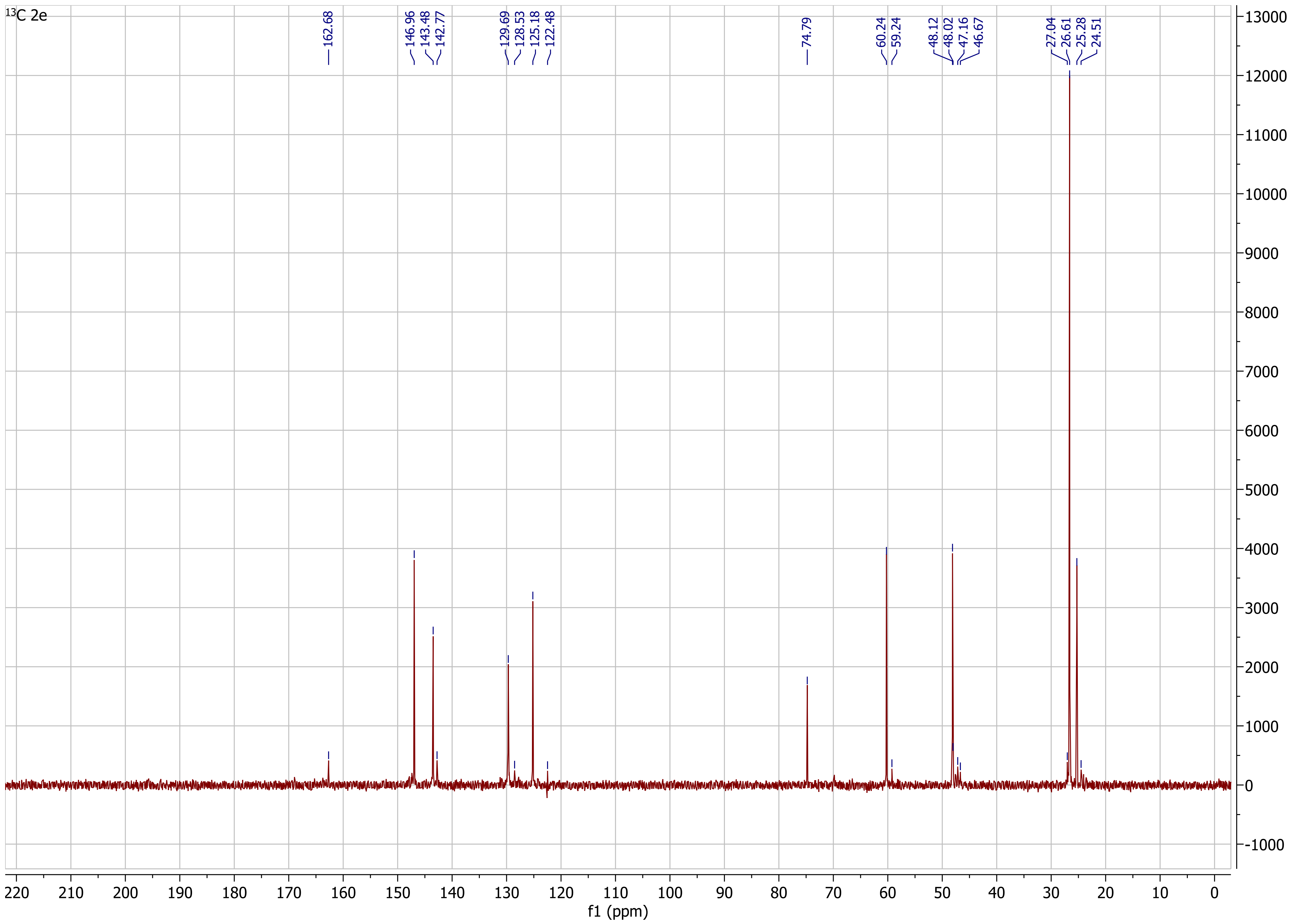

13C 2e
162.68
146.96
143.48
142.77
129.69
128.53
125.18
122.48
74.79
60.24
59.24
48.12
48.02
47.16
46.67
27.04
26.61
25.28
24.51
f1 (ppm)

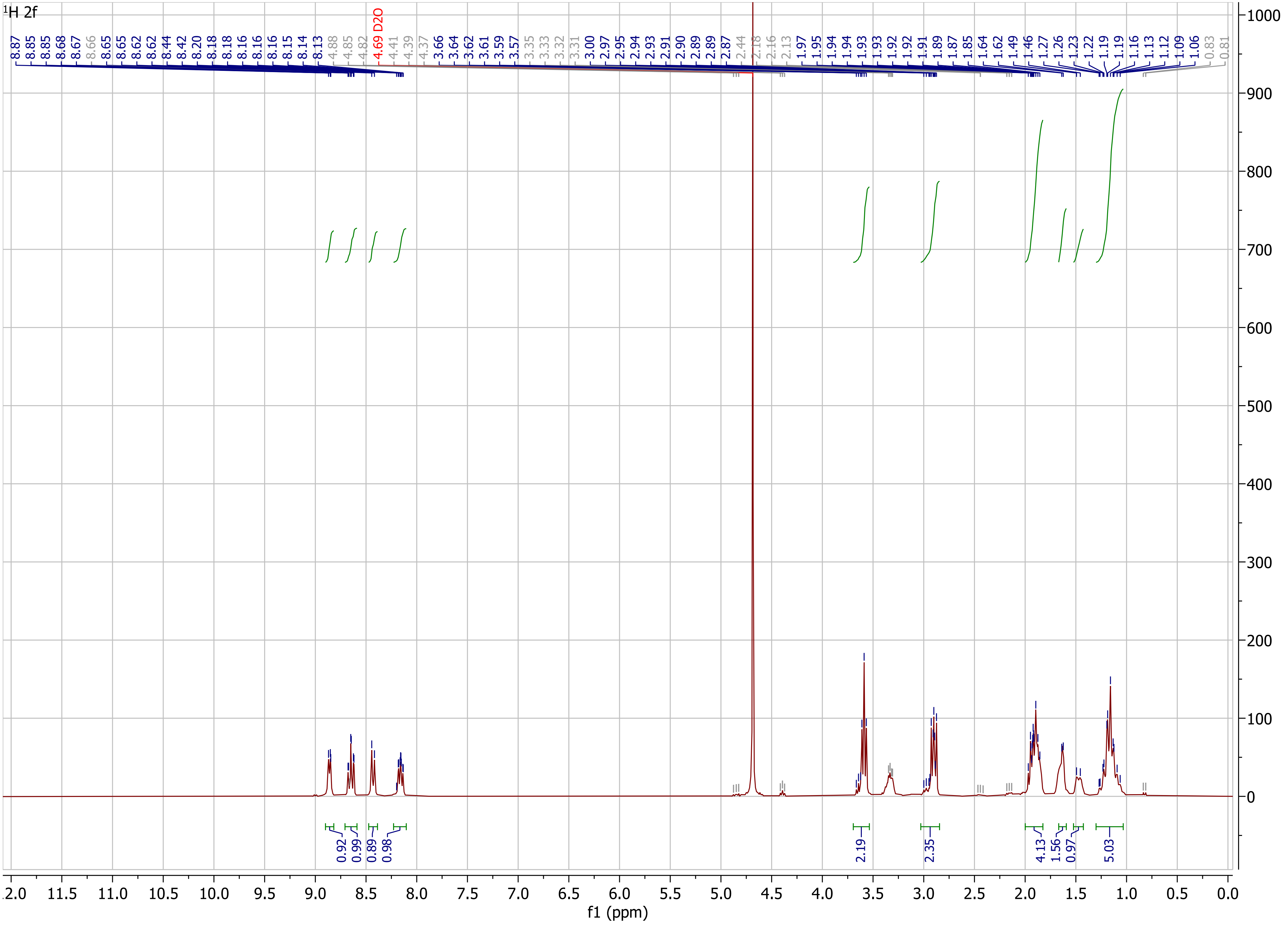

1H 2f
4.69 D2O
8.87
8.85
8.85
8.68
8.67
8.66
8.65
8.65
8.62
8.62
8.44
8.42
8.20
8.18
8.18
8.16
8.16
8.16
8.15
8.14
8.13
4.88
4.85
4.82
4.41
4.39
4.37
3.66
3.64
3.62
3.61
3.59
3.57
3.35
3.33
3.32
3.31
3.00
2.97
2.95
2.94
2.93
2.91
2.90
2.89
2.89
2.87
2.44
2.18
2.16
2.13
1.97
1.95
1.94
1.94
1.93
1.93
1.92
1.92
1.91
1.89
1.87
1.85
1.64
1.62
1.49
1.46
1.27
1.26
1.23
1.22
1.19
1.19
1.16
1.13
1.12
1.09
1.06
0.83
0.81
0.92
0.99
0.89
0.98
2.19
2.35
4.13
1.56
0.97
5.03
f1 (ppm)

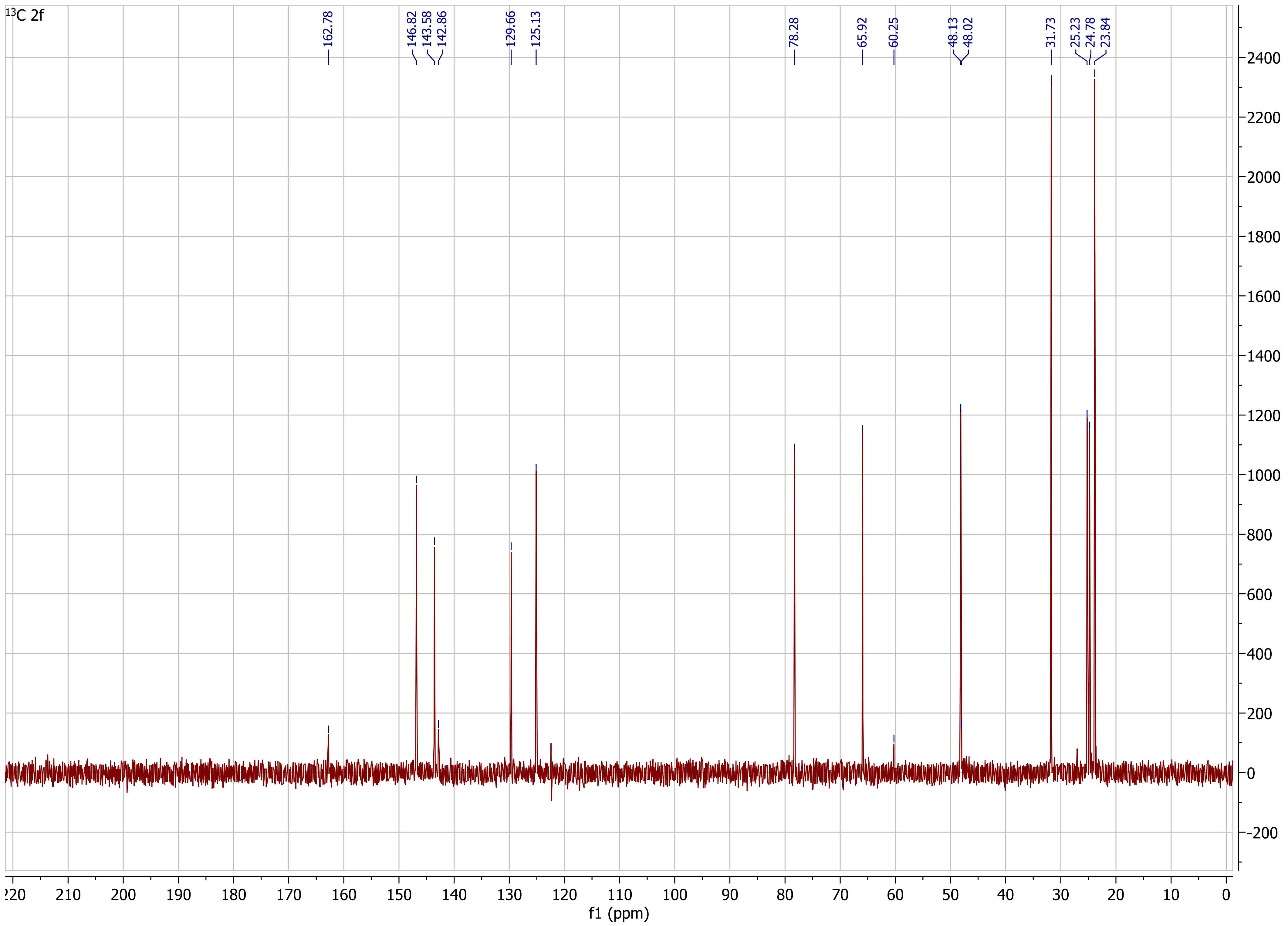

13C 2f
162.78
146.82
143.58
142.86
129.66
125.13
78.28
65.92
60.25
48.13
48.02
31.73
25.23
24.78
23.84
f1 (ppm)

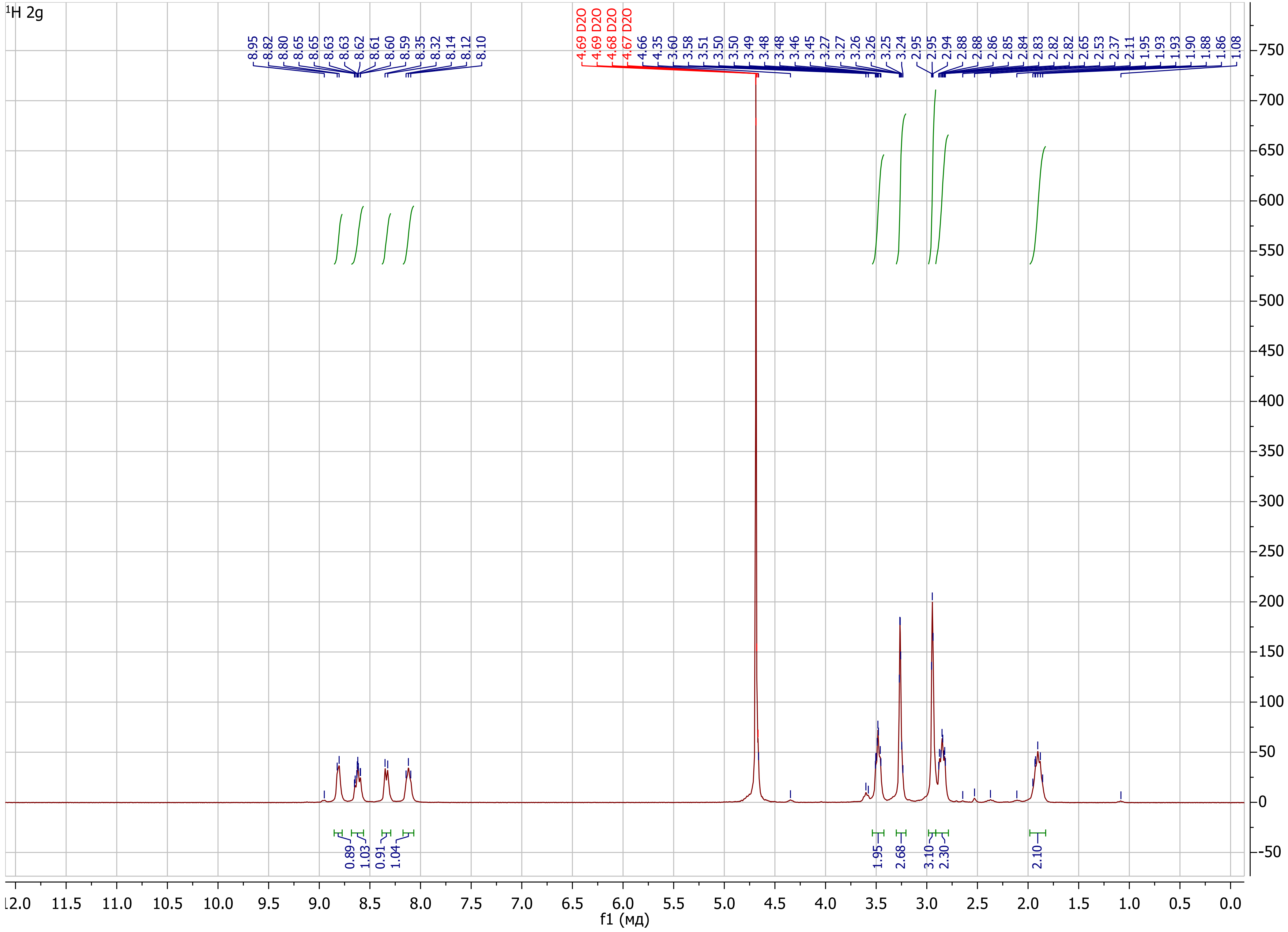

1H 2g
8.95 8.82 8.80 8.65 8.65 8.63 8.63 8.62 8.61 8.60 8.59 8.35 8.32 8.14 8.12 8.10
4.69 D2O 4.69 D2O 4.68 D2O 4.67 D2O
4.66 4.35 3.60 3.58 3.51 3.50 3.50 3.49 3.48 3.48 3.46 3.45 3.27 3.27 3.26 3.26 3.25 3.24 2.95 2.95 2.94 2.88 2.88 2.86 2.85 2.84 2.83 2.82 2.82 2.65 2.53 2.37 2.11 1.95 1.93 1.93 1.90 1.88 1.86 1.08
0.89 1.03 0.91 1.04 1.95 2.68 3.10 2.30 2.10
f1 (мд)

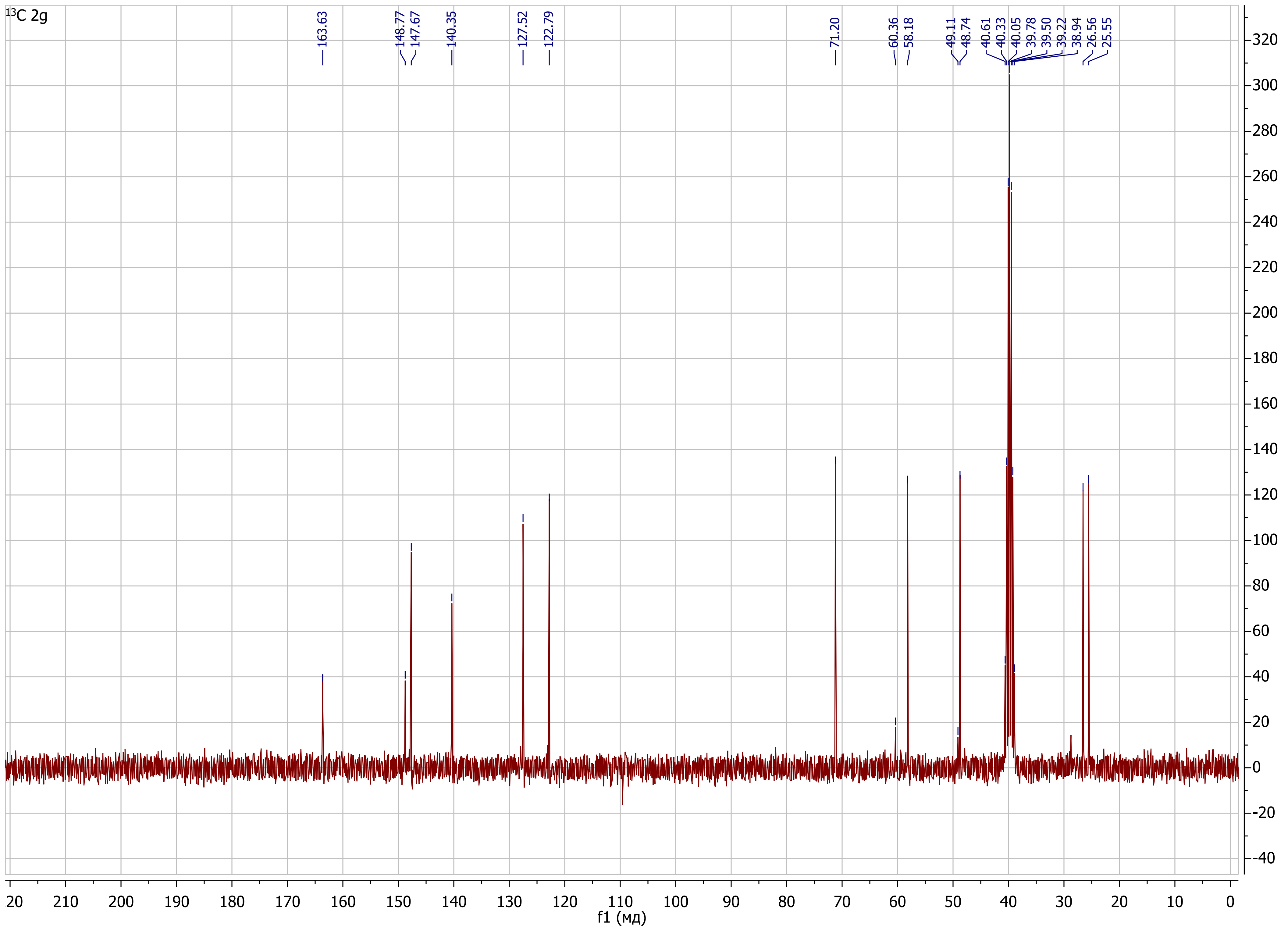

13C 2g
163.63
148.77
147.67
140.35
127.52
122.79
71.20
60.36
58.18
49.11
48.74
40.61
40.33
40.05
39.78
39.50
39.22
38.94
26.56
25.55
f1 (мд)

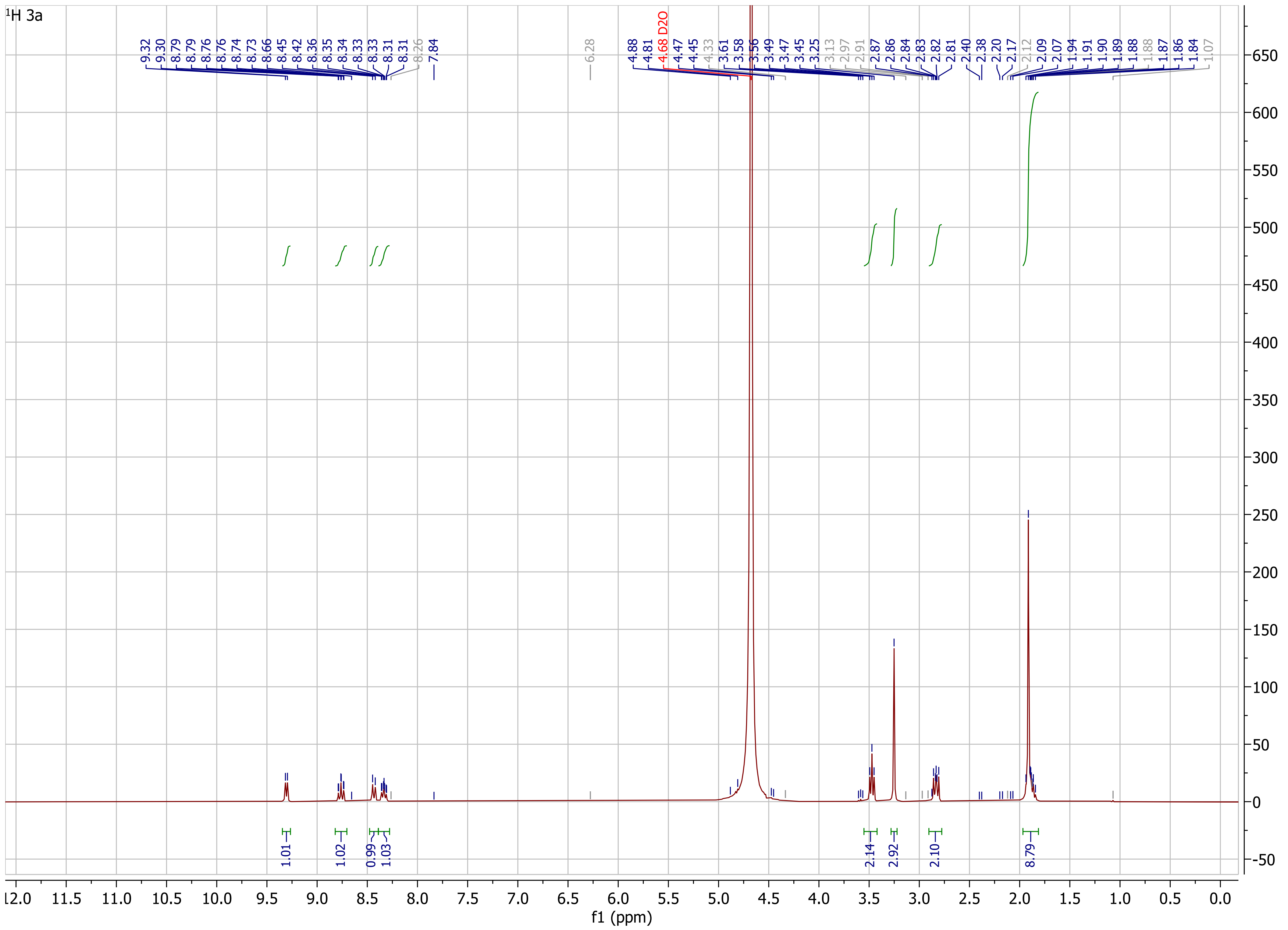

¹H 3a
9.32 9.30 8.79 8.79 8.76 8.76 8.74 8.73 8.66 8.45 8.42 8.36 8.35 8.34 8.33 8.33 8.31 8.31 8.26 7.84
6.28
4.88 4.81 4.68 D2O 4.47 4.45 4.33 3.61 3.58 3.56 3.49 3.47 3.45 3.25 3.13 2.97 2.91 2.87 2.86 2.84 2.83 2.82 2.81 2.40 2.38 2.20 2.17 2.12 2.09 2.07 1.94 1.91 1.90 1.89 1.88 1.88 1.87 1.86 1.84 1.07
1.01 1.02 0.99 1.03 2.14 2.92 2.10 8.79
f1 (ppm)

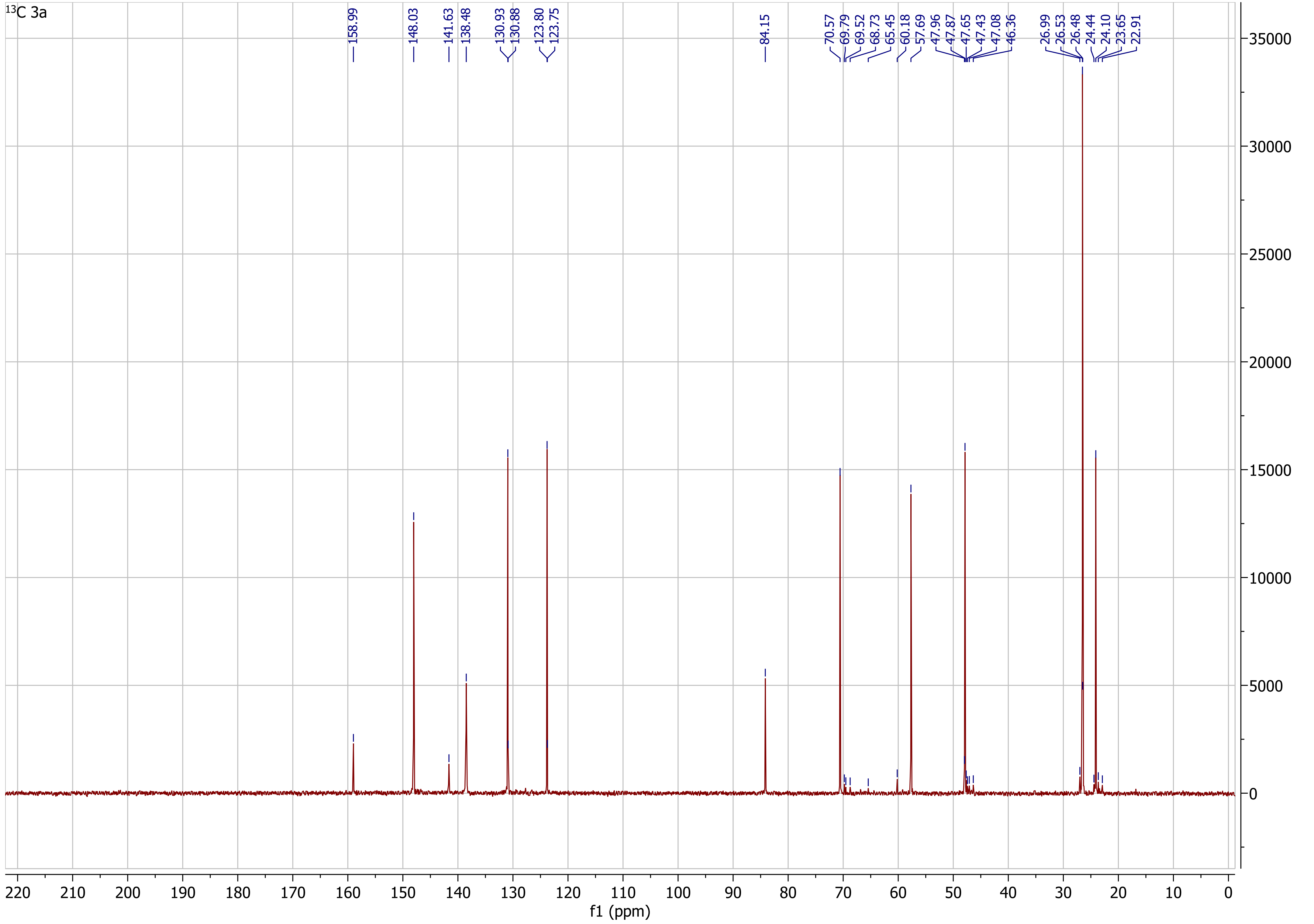

13C 3a
158.99
148.03
141.63
138.48
130.93
130.88
123.80
123.75
84.15
70.57
69.79
69.52
68.73
65.45
60.18
57.69
47.96
47.87
47.65
47.43
47.08
46.36
26.99
26.53
26.48
24.44
24.10
23.65
22.91
35000
30000
25000
20000
15000
10000
5000
0
220 210 200 190 180 170 160 150 140 130 120 110 100 90 80 70 60 50 40 30 20 10 0
f1 (ppm)

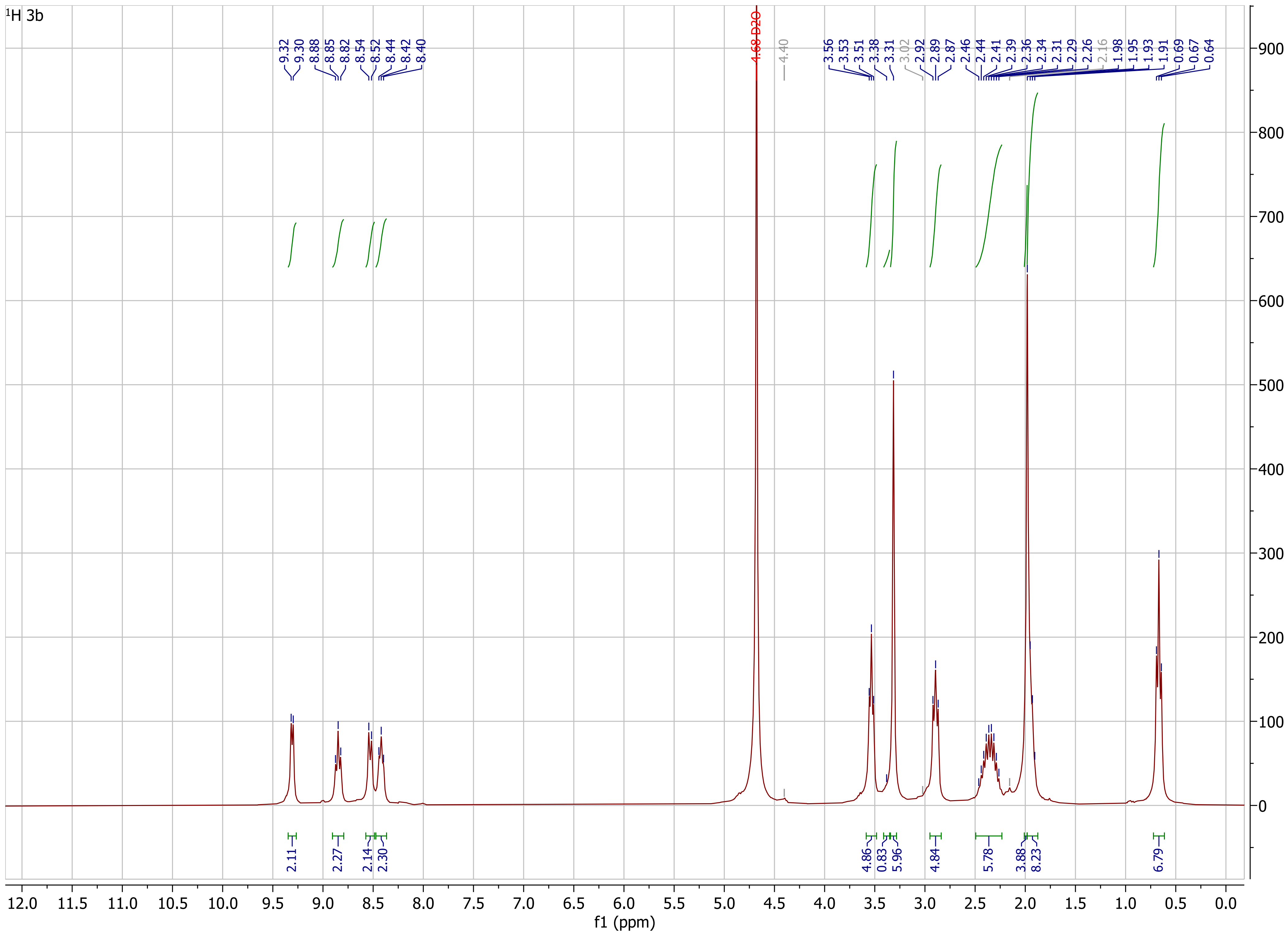

¹H 3b
9.32
9.30
8.88
8.85
8.82
8.54
8.52
8.44
8.42
8.40
4.68 D2O
4.40
3.56
3.53
3.51
3.38
3.31
3.02
2.92
2.89
2.87
2.46
2.44
2.41
2.39
2.36
2.34
2.31
2.29
2.26
2.16
1.98
1.95
1.93
1.91
0.69
0.67
0.64
2.11
2.27
2.14
2.30
4.86
0.83
5.96
4.84
5.78
3.88
8.23
6.79
f1 (ppm)

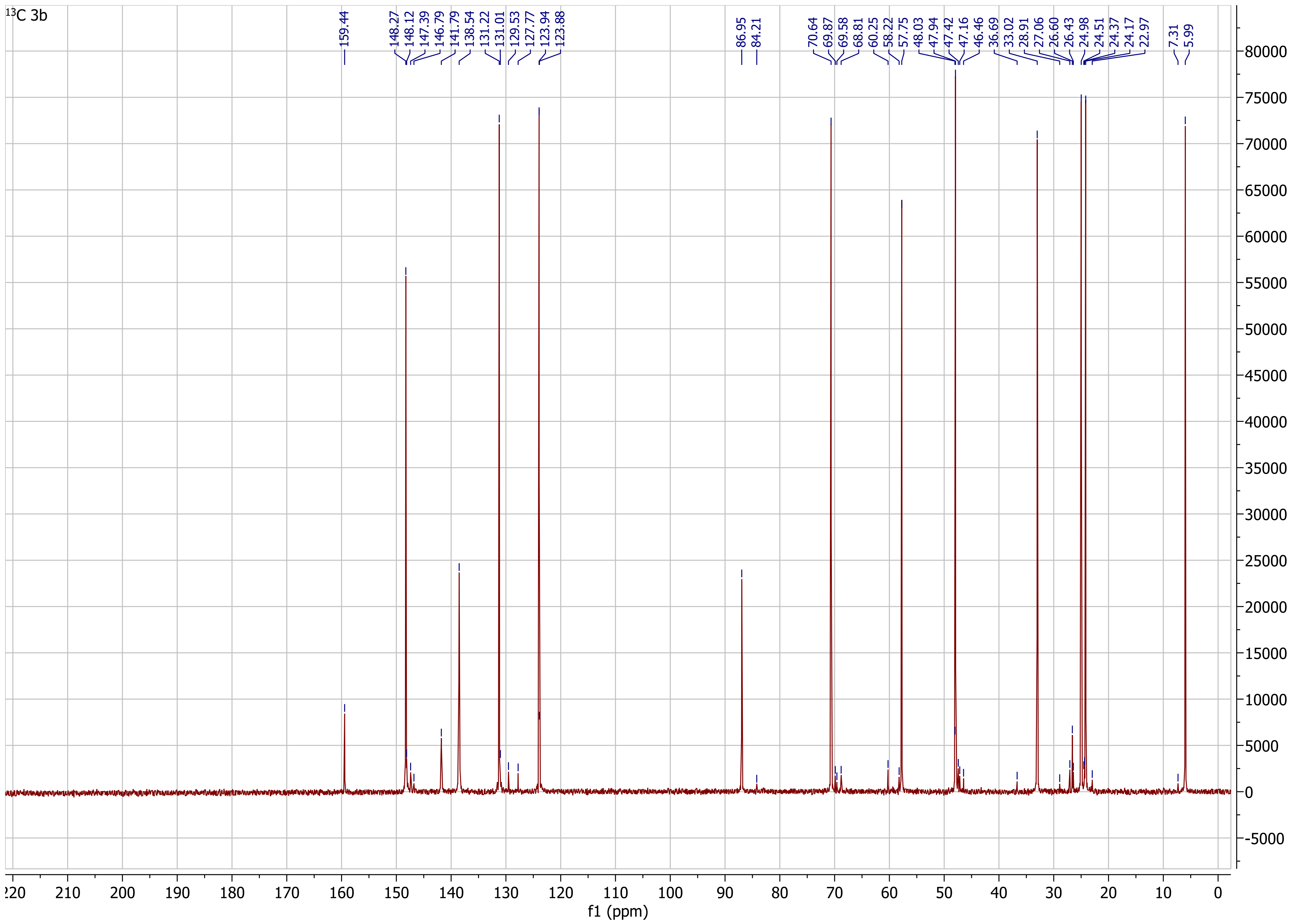

13C 3b
159.44
148.27
148.12
147.39
146.79
141.79
138.54
131.22
131.01
129.53
127.77
123.94
123.88
86.95
84.21
70.64
69.87
69.58
68.81
60.25
58.22
57.75
48.03
47.94
47.42
47.16
46.46
36.69
33.02
28.91
27.06
26.60
26.43
24.98
24.51
24.37
24.17
22.97
7.31
5.99
f1 (ppm)

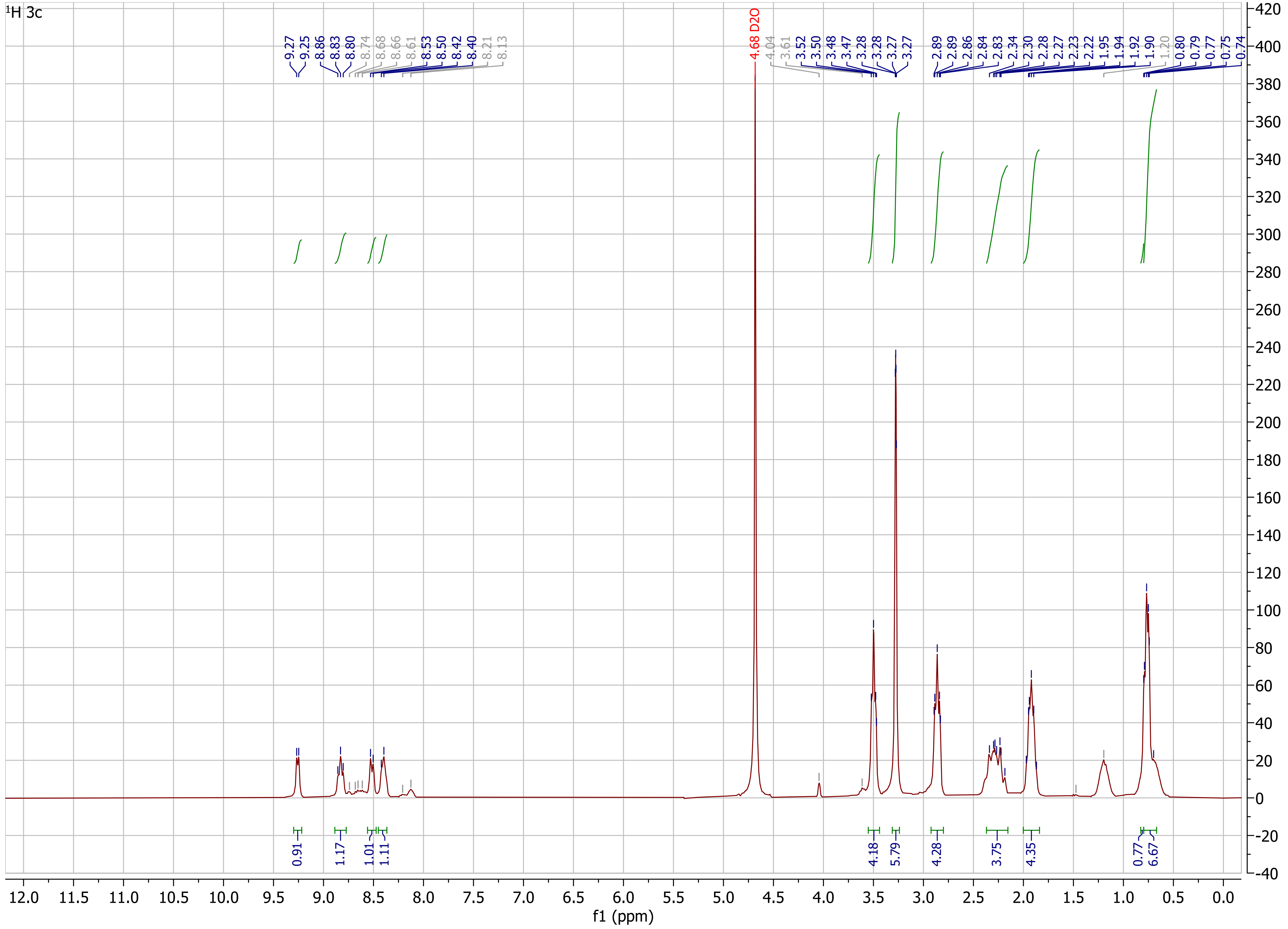
1H 3c
9.27
9.25
8.86
8.83
8.80
8.74
8.68
8.66
8.61
8.53
8.50
8.42
8.40
8.21
8.13
4.68 D2O
4.04
3.61
3.52
3.50
3.48
3.47
3.28
3.28
3.27
3.27
2.89
2.89
2.86
2.84
2.83
2.34
2.30
2.28
2.27
2.23
2.22
1.95
1.94
1.92
1.90
1.20
0.80
0.79
0.77
0.75
0.74
0.91
1.17
1.01
1.11
4.18
5.79
4.28
3.75
4.35
0.77
6.67
f1 (ppm)

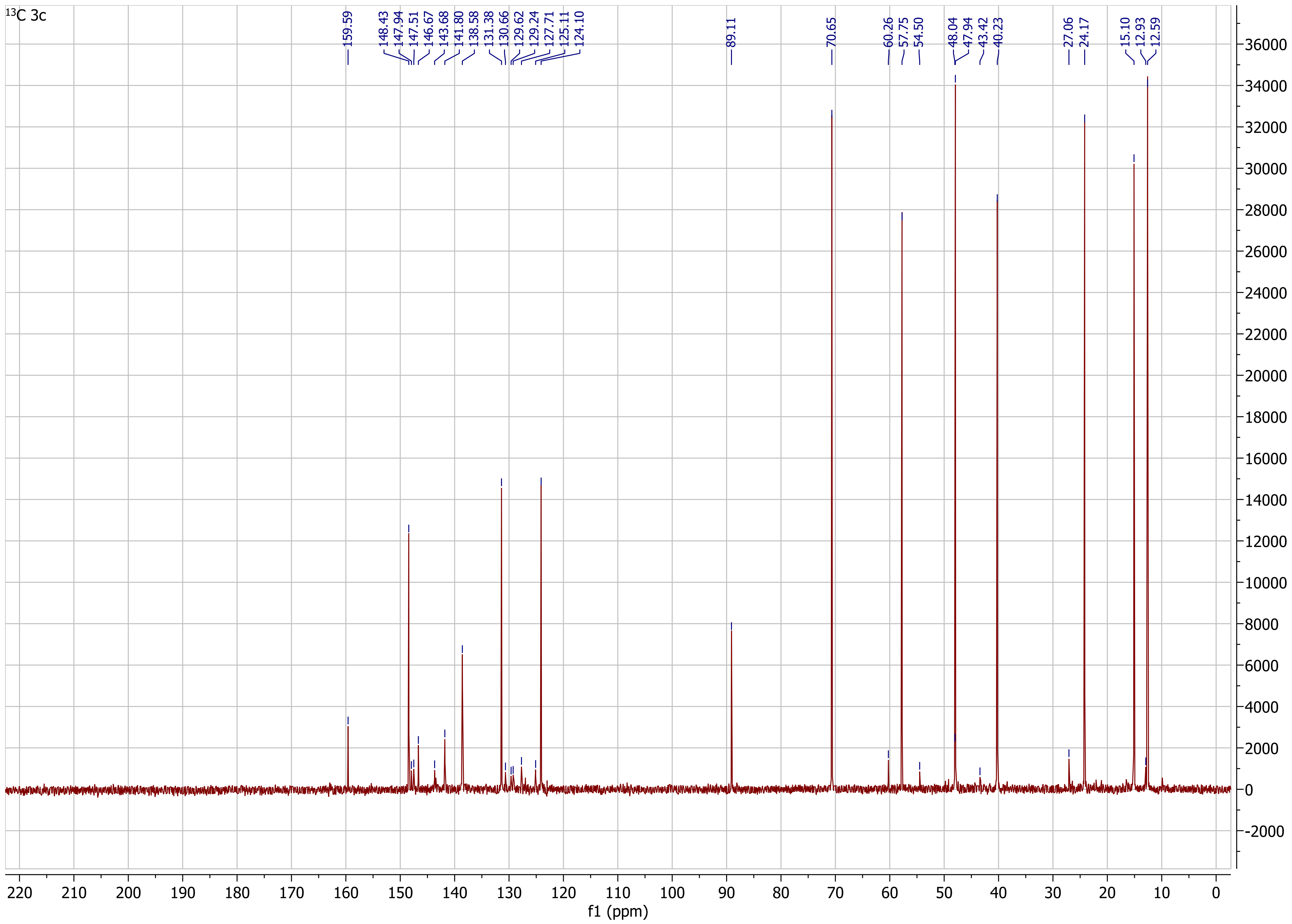

13C 3c
159.59
148.43
147.94
147.51
146.67
143.68
141.80
138.58
131.38
130.66
129.62
129.24
127.71
125.11
124.10
89.11
70.65
60.26
57.75
54.50
48.04
47.94
43.42
40.23
27.06
24.17
15.10
12.93
12.59
f1 (ppm)

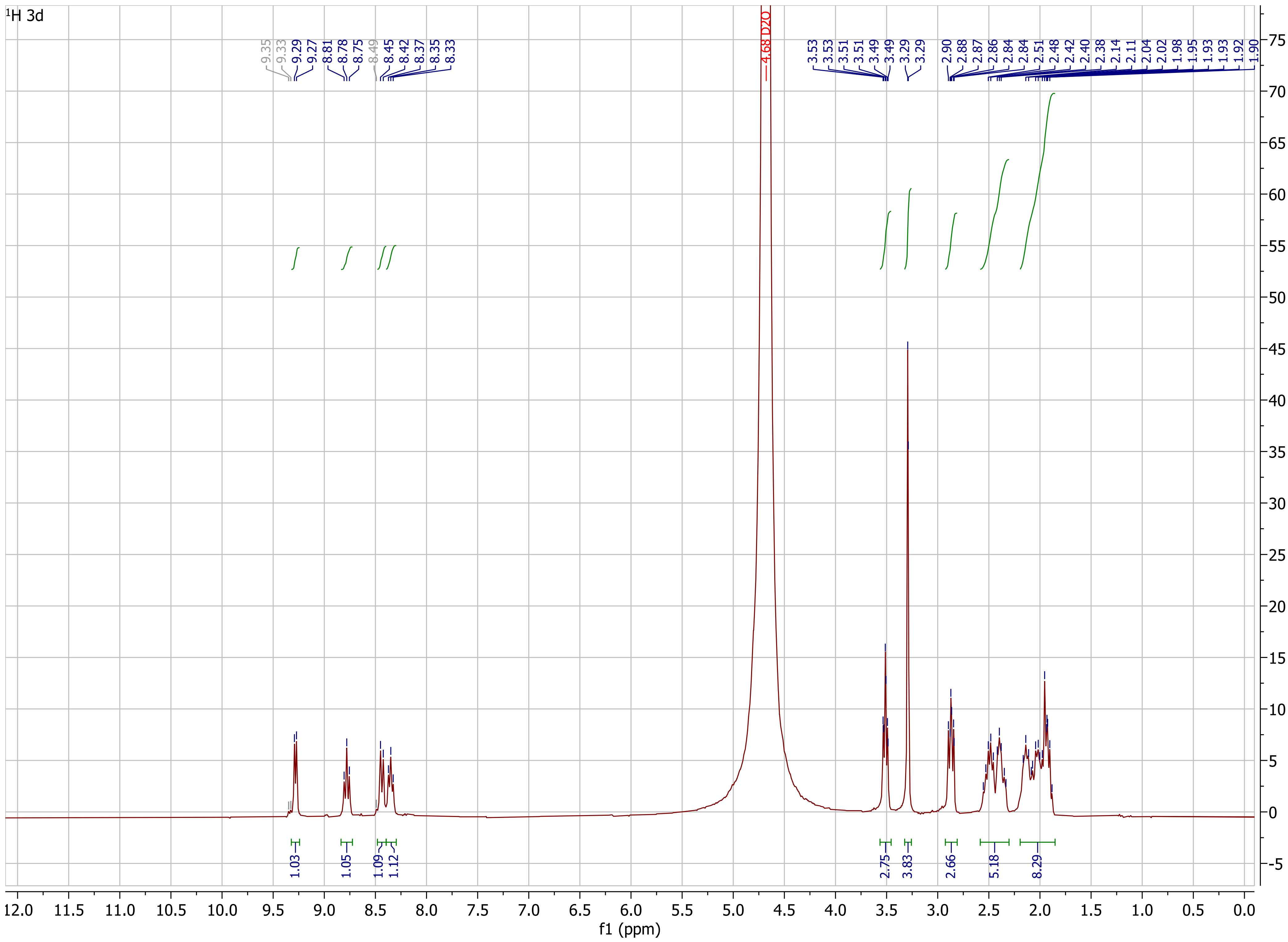

[1]H 3d
9.35
9.33
9.29
9.27
8.81
8.78
8.75
8.49
8.45
8.42
8.37
8.35
8.33
4.68 D2O
3.53
3.53
3.51
3.51
3.49
3.49
3.29
3.29
2.90
2.88
2.87
2.86
2.84
2.84
2.51
2.48
2.42
2.40
2.38
2.14
2.11
2.04
2.02
1.98
1.95
1.93
1.93
1.92
1.90
1.03
1.05
1.09
1.12
2.75
3.83
2.66
5.18
8.29
f1 (ppm)

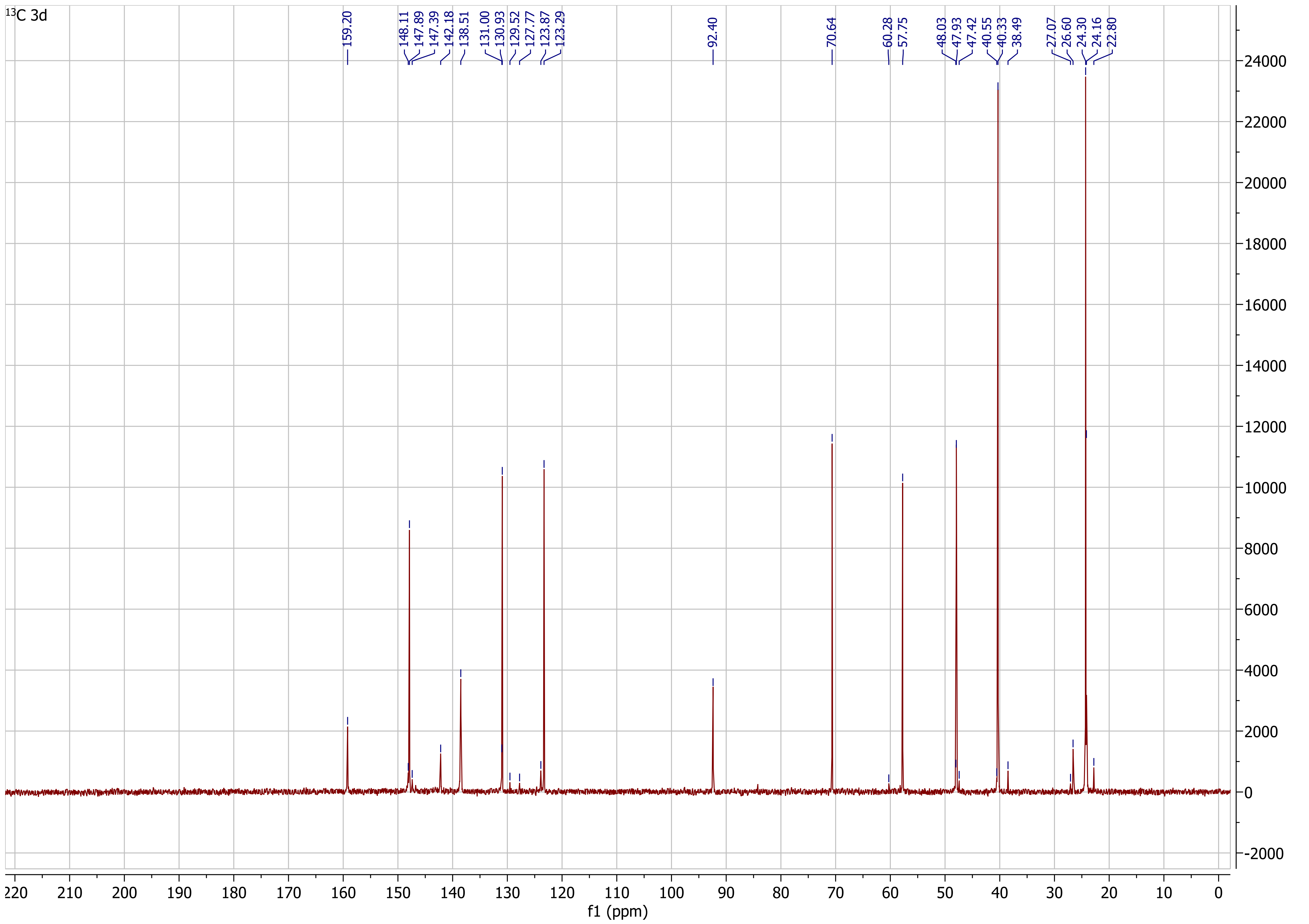

13C 3d
159.20
148.11
147.89
147.39
142.18
138.51
131.00
130.93
129.52
127.77
123.87
123.29
92.40
70.64
60.28
57.75
48.03
47.93
47.42
40.55
40.33
38.49
27.07
26.60
24.30
24.16
22.80
f1 (ppm)

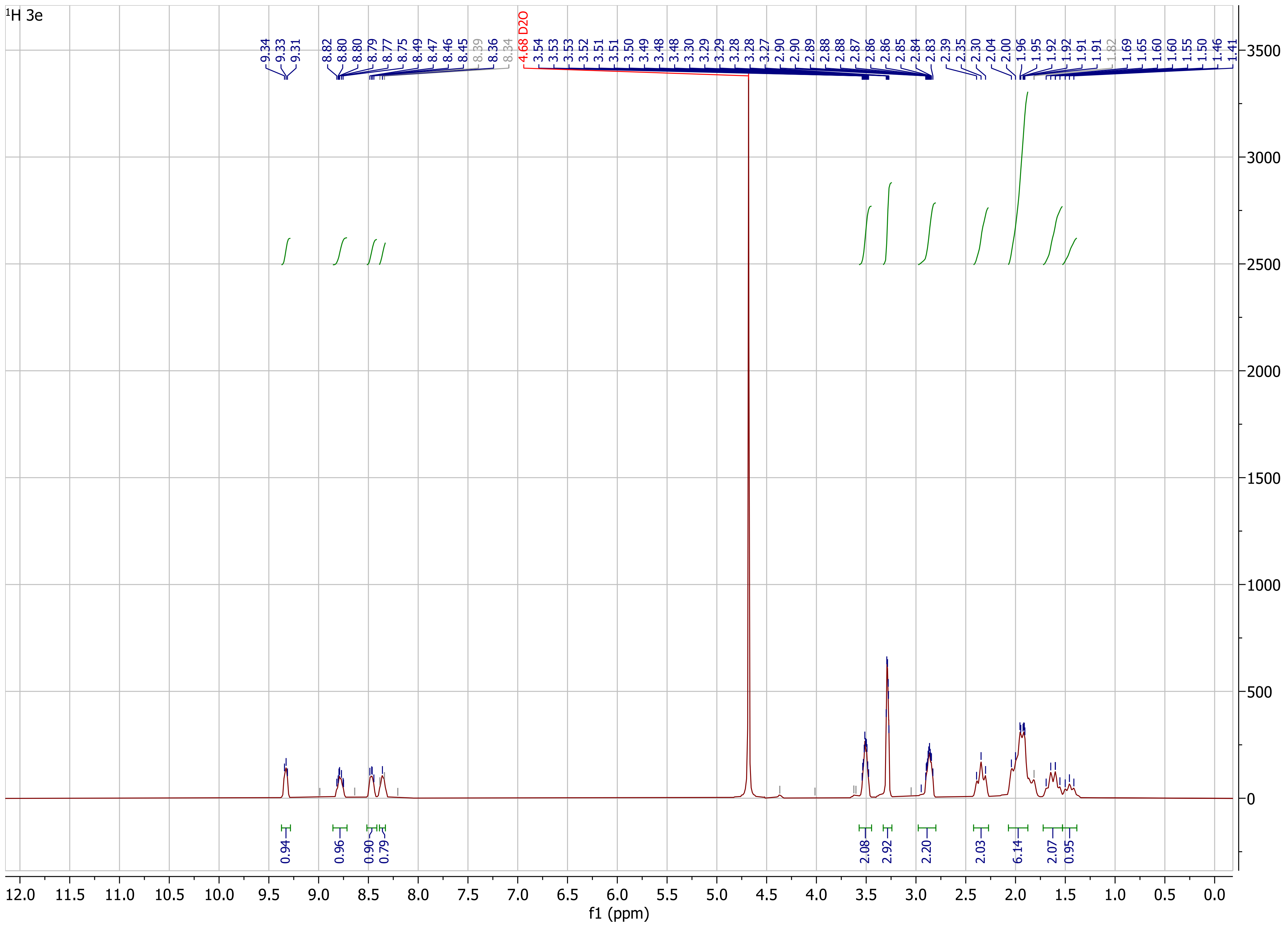

¹H 3e
4.68 D2O
f1 (ppm)

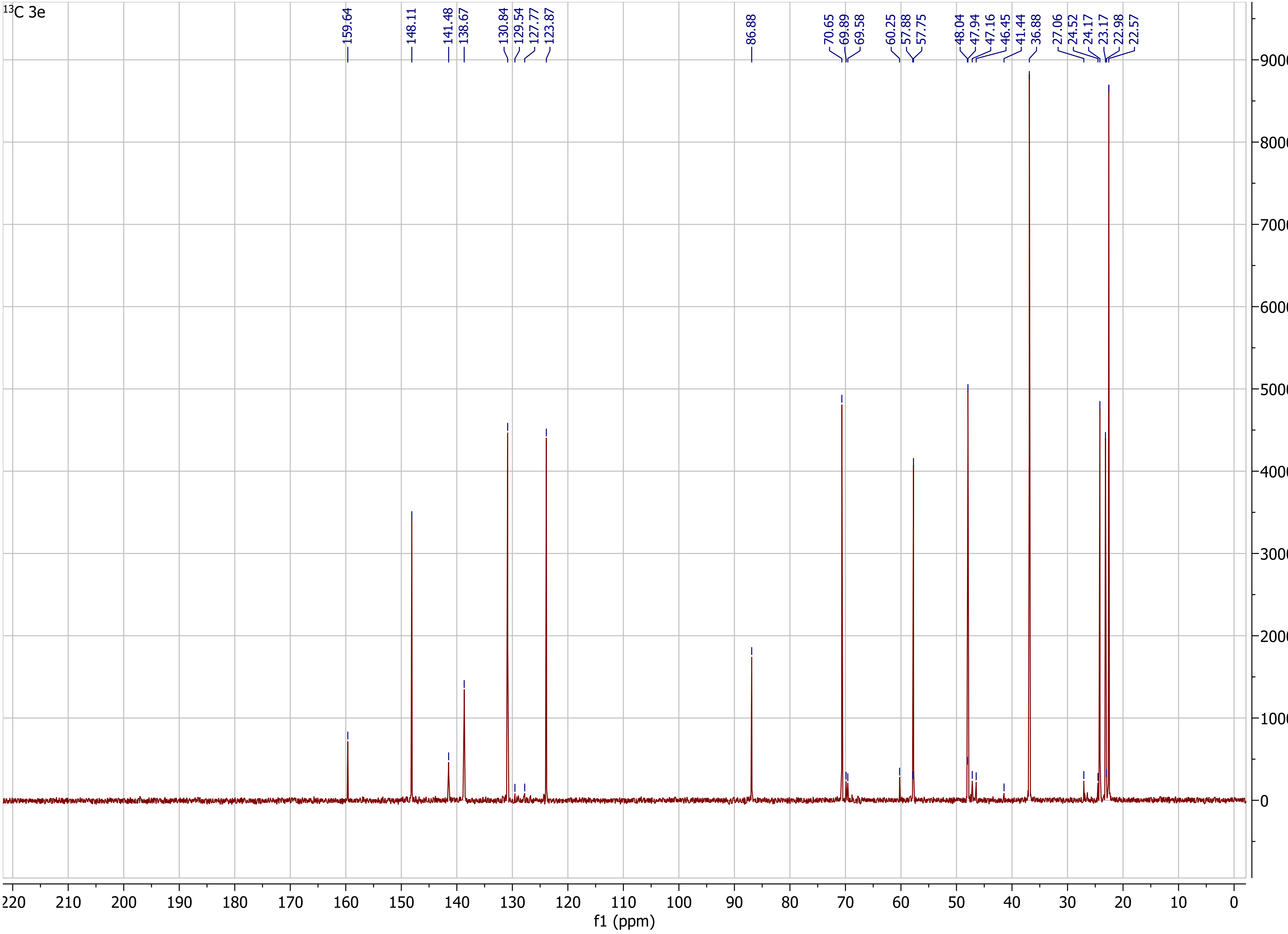

13C 3e
159.64
148.11
141.48
138.67
130.84
129.54
127.77
123.87
86.88
70.65
69.89
69.58
60.25
57.88
57.75
48.04
47.94
47.16
46.45
41.44
36.88
27.06
24.52
24.17
23.17
22.98
22.57
f1 (ppm)

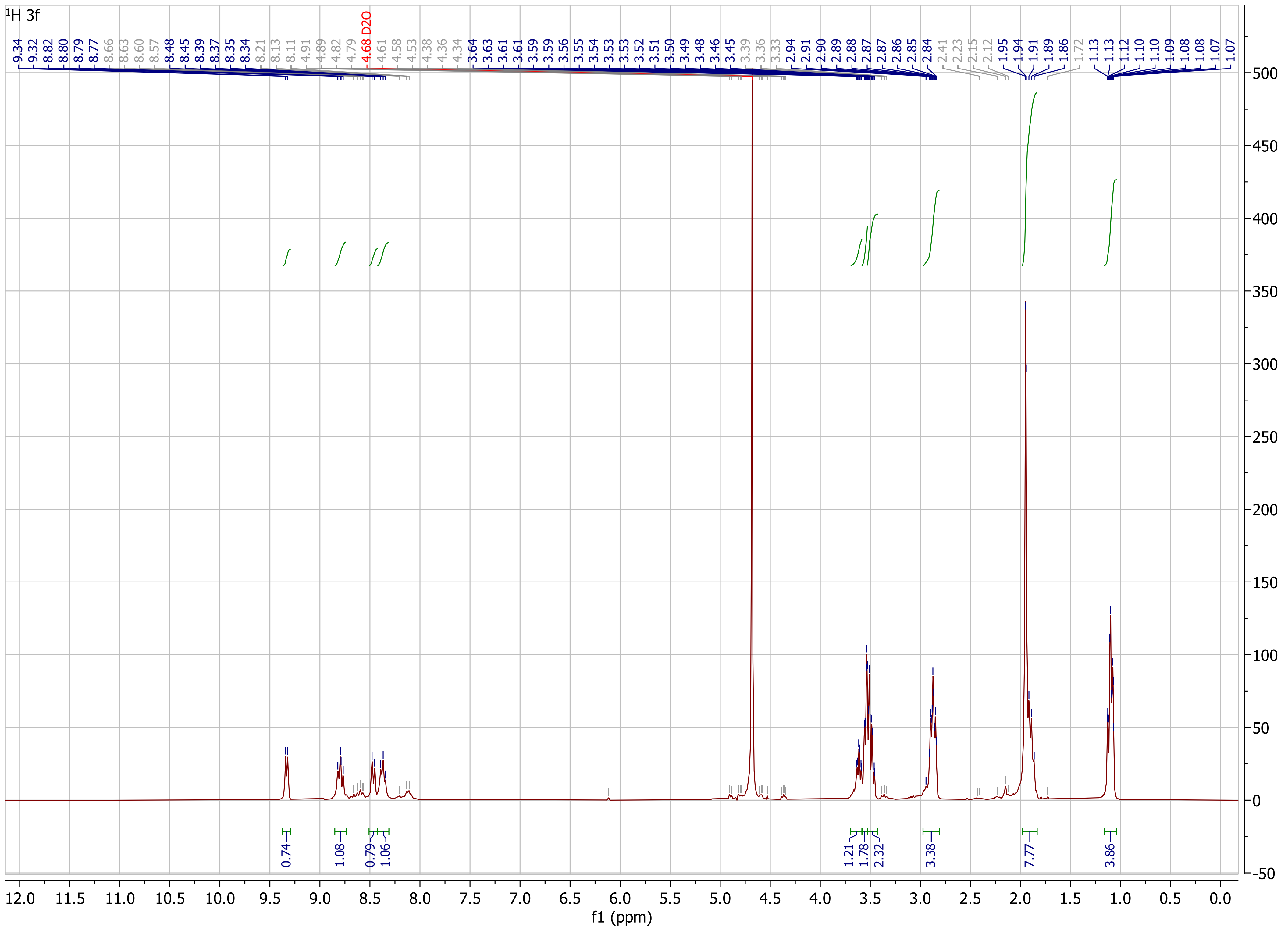

¹H 3f
9.34
9.32
8.82
8.80
8.79
8.77
8.66
8.63
8.60
8.57
8.48
8.45
8.39
8.37
8.35
8.34
8.21
8.13
8.11
4.91
4.89
4.82
4.79
4.68 D2O
4.61
4.58
4.53
4.38
4.36
4.34
3.64
3.63
3.61
3.61
3.59
3.59
3.56
3.55
3.54
3.53
3.53
3.52
3.51
3.50
3.49
3.48
3.46
3.45
3.39
3.36
3.33
2.94
2.91
2.90
2.89
2.88
2.87
2.87
2.86
2.85
2.84
2.41
2.23
2.15
2.12
1.95
1.94
1.91
1.89
1.86
1.72
1.13
1.13
1.12
1.10
1.10
1.09
1.08
1.08
1.07
1.07
0.74
1.08
0.79
1.06
1.21
1.78
2.32
3.38
7.77
3.86
f1 (ppm)

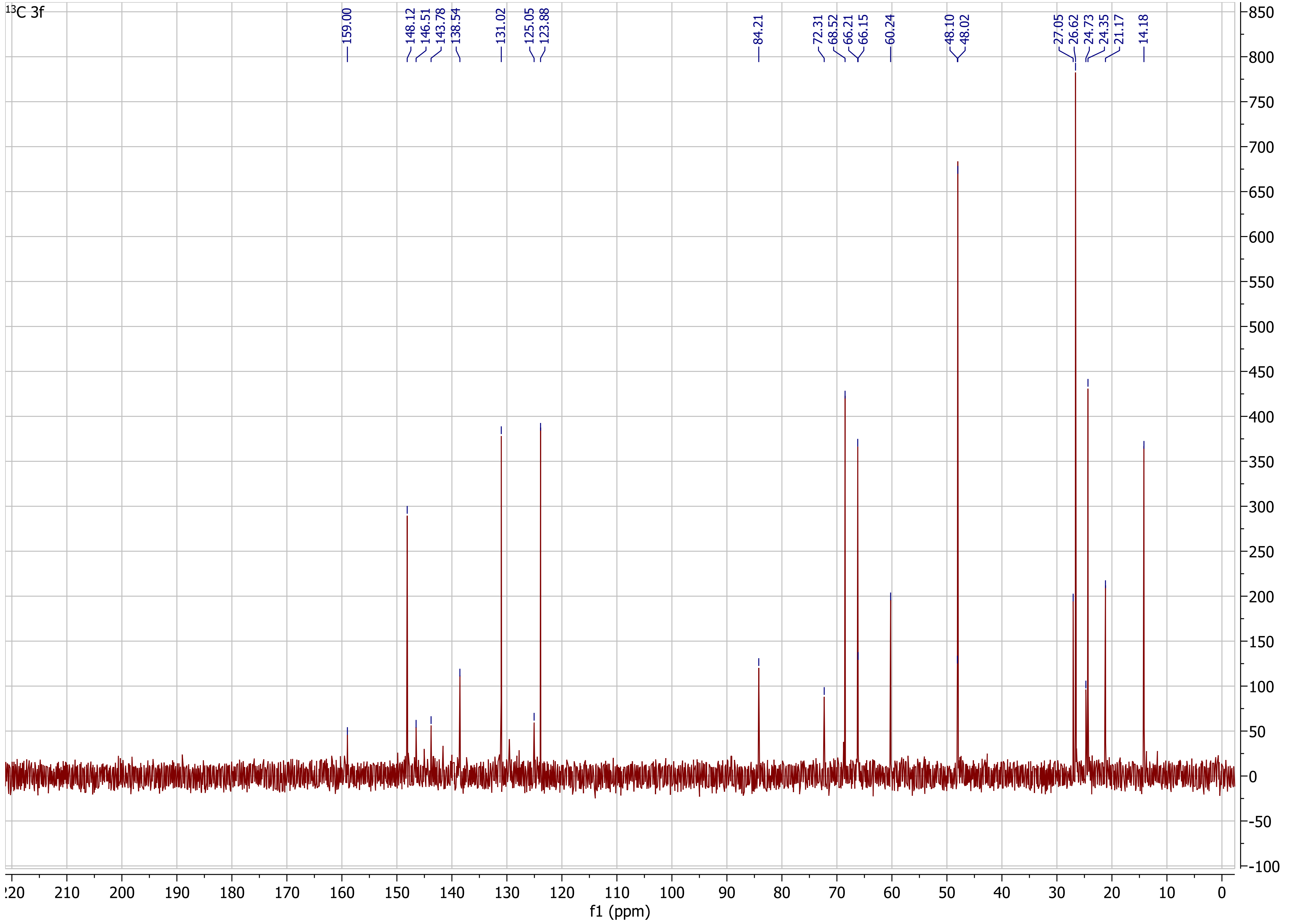

13C 3f
159.00
148.12
146.51
143.78
138.54
131.02
125.05
123.88
84.21
72.31
68.52
66.21
66.15
60.24
48.10
48.02
27.05
26.62
24.73
24.35
21.17
14.18
f1 (ppm)

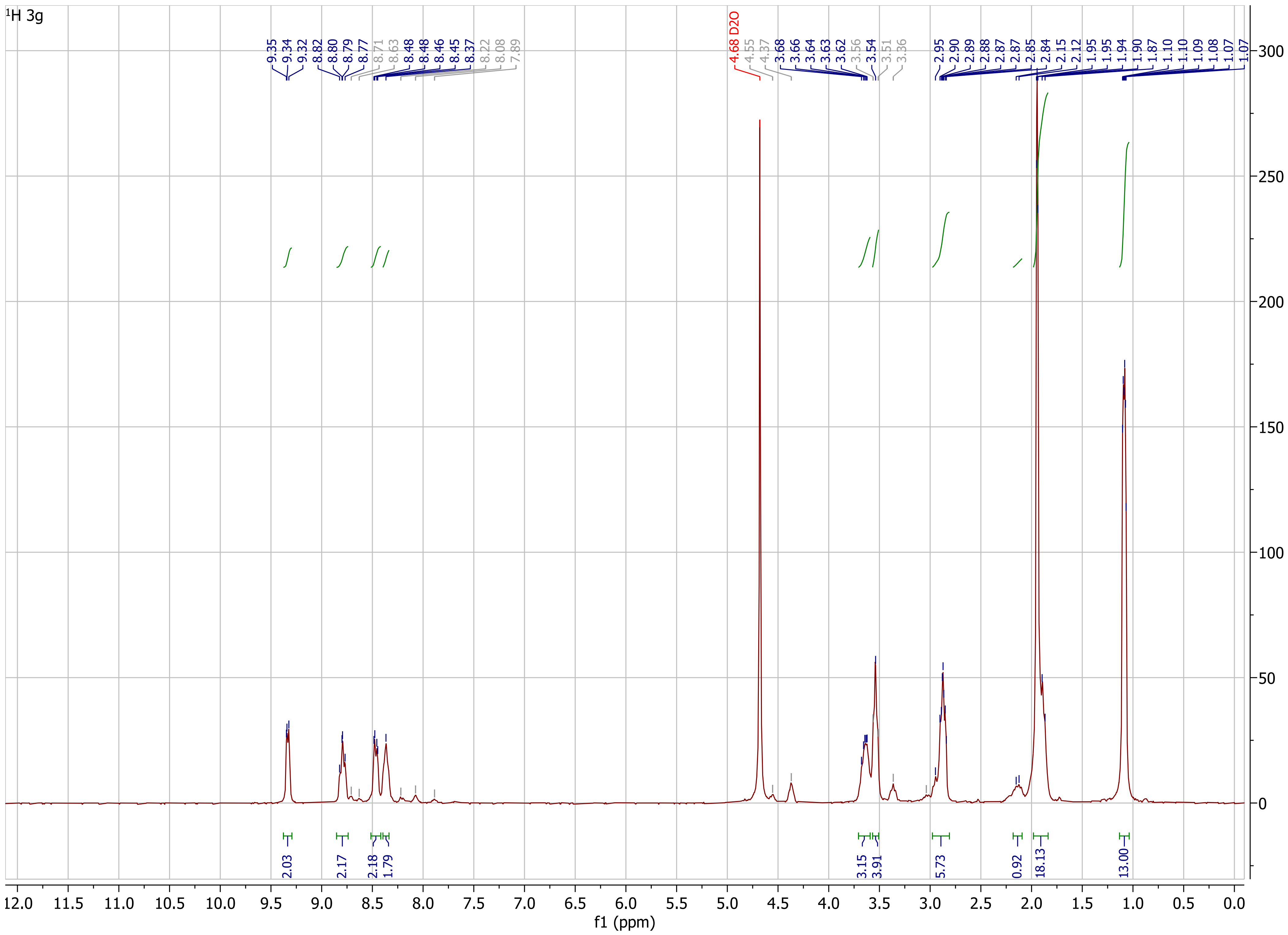

1H 3g
9.35 9.34 9.32 8.82 8.80 8.79 8.77 8.71 8.63 8.48 8.48 8.46 8.45 8.37 8.22 8.08 7.89
4.68 D2O
4.55 4.37 3.68 3.66 3.64 3.63 3.62 3.56 3.54 3.51 3.36 2.95 2.90 2.89 2.88 2.87 2.87 2.85 2.84 2.15 2.12 1.95 1.95 1.94 1.90 1.87 1.10 1.10 1.09 1.08 1.07 1.07
2.03 2.17 2.18 1.79 3.15 3.91 5.73 0.92 18.13 13.00
f1 (ppm)

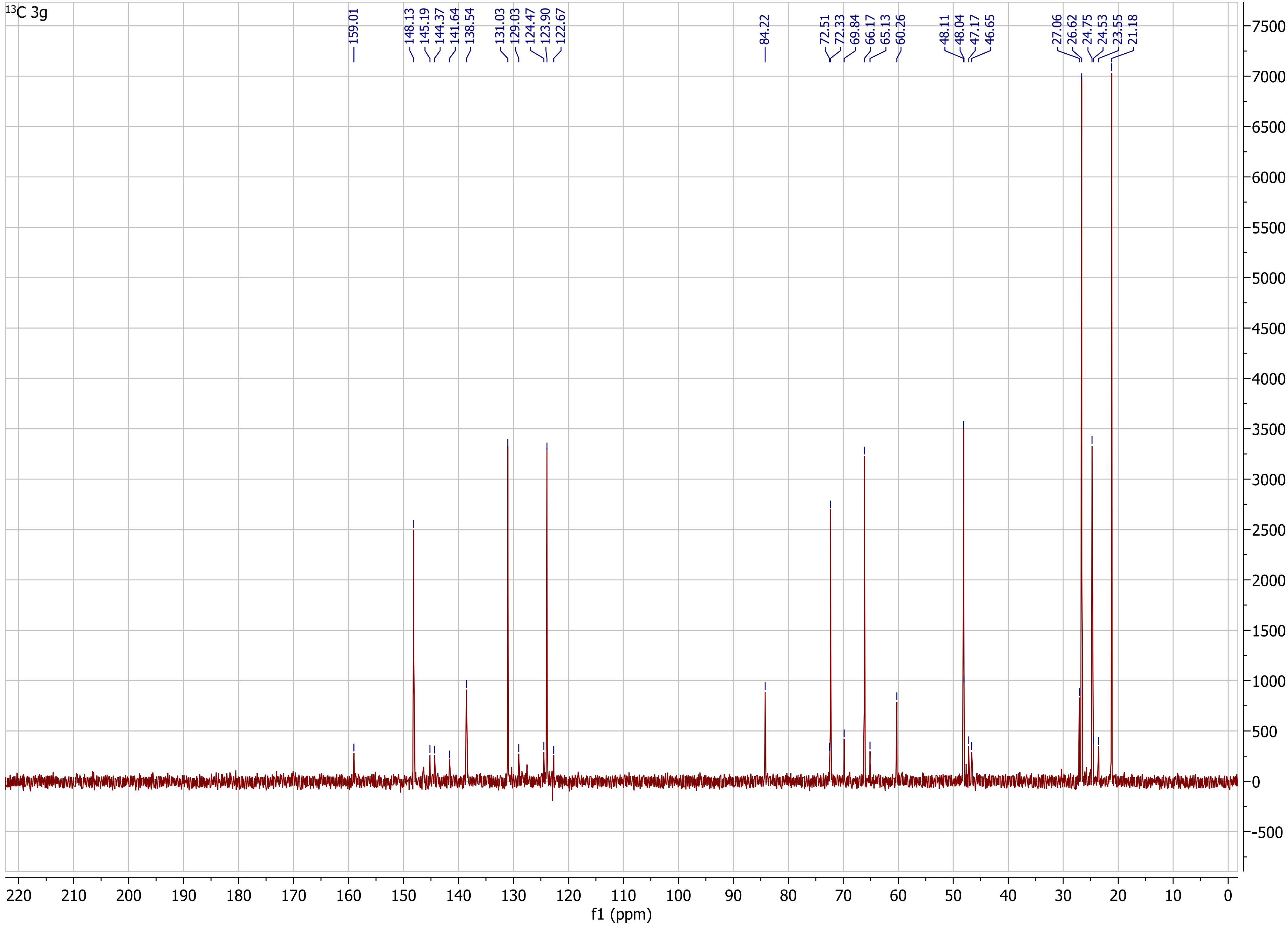

13C 3g
159.01
148.13
145.19
144.37
141.64
138.54
131.03
129.03
124.47
123.90
122.67
84.22
72.51
72.33
69.84
66.17
65.13
60.26
48.11
48.04
47.17
46.65
27.06
26.62
24.75
24.53
23.55
21.18
f1 (ppm)

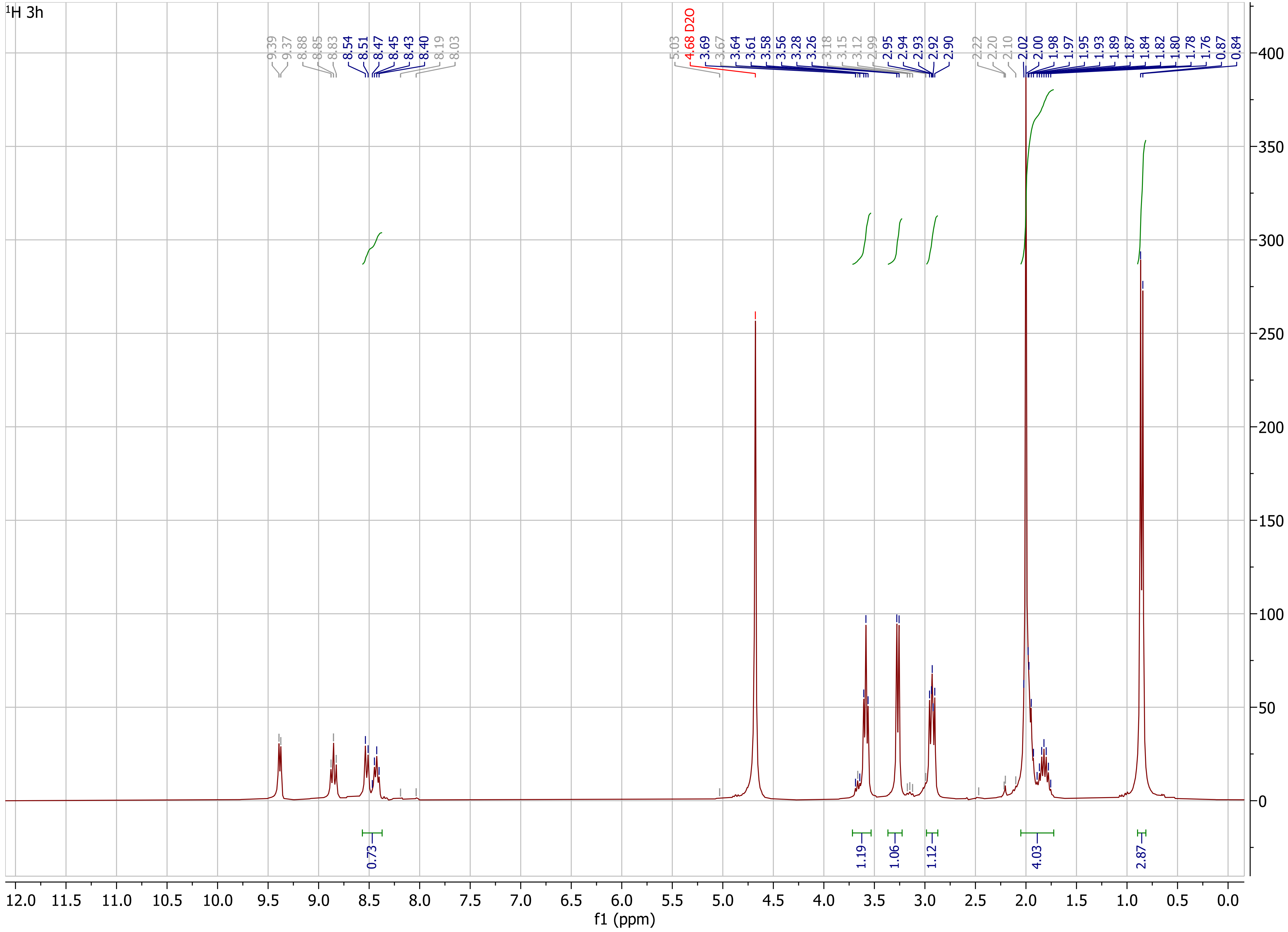

^{1}H 3h
9.39
9.37
8.88
8.85
8.83
8.54
8.51
8.47
8.45
8.43
8.40
8.19
8.03
5.03
4.68 D2O
3.69
3.67
3.64
3.61
3.58
3.56
3.28
3.26
3.18
3.15
3.12
2.99
2.95
2.94
2.93
2.92
2.90
2.22
2.20
2.10
2.02
2.00
1.98
1.97
1.95
1.93
1.89
1.87
1.84
1.82
1.80
1.78
1.76
0.87
0.84
0.73
1.19
1.06
1.12
4.03
2.87
f1 (ppm)

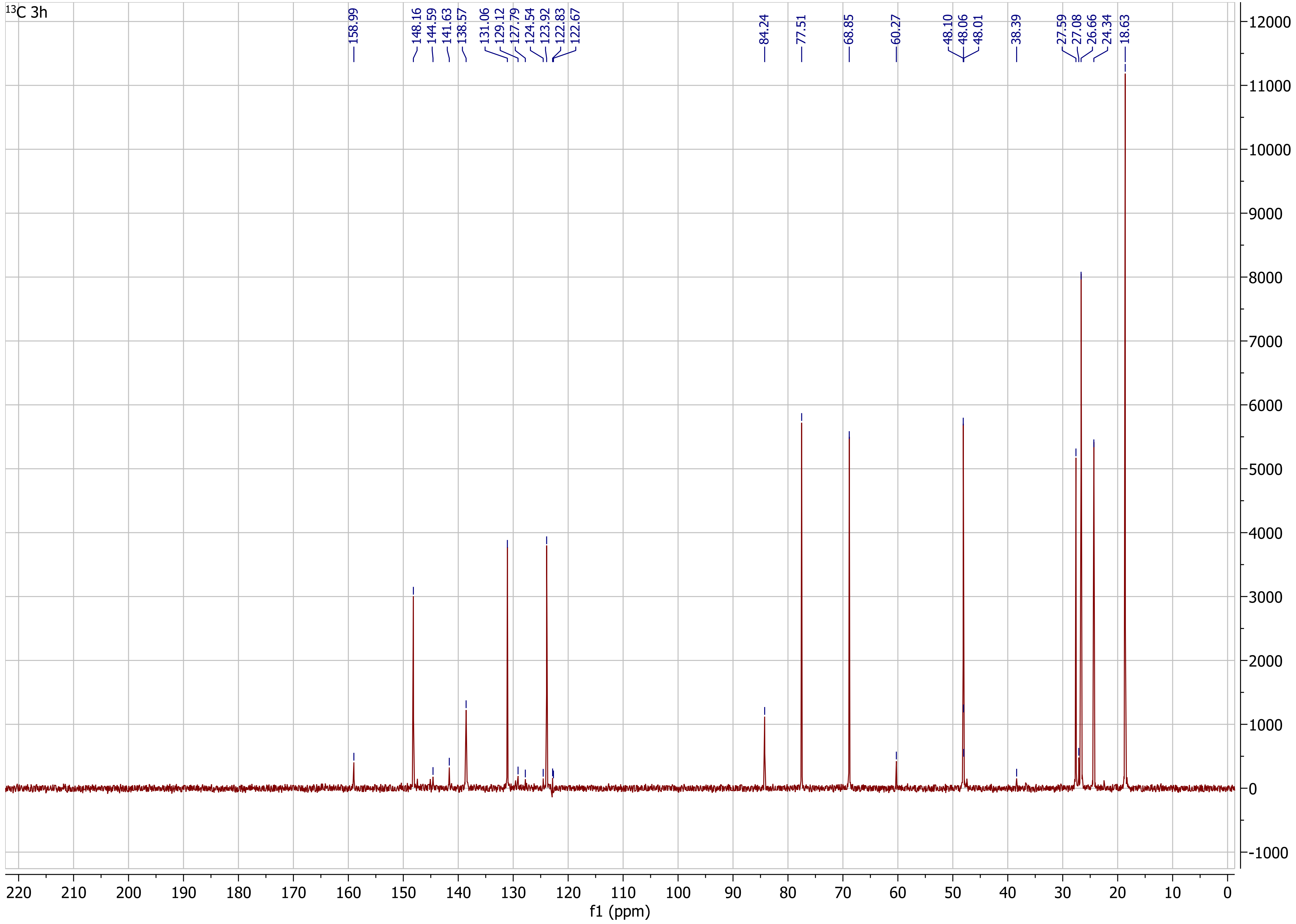

13C 3h
158.99
148.16
144.59
141.63
138.57
131.06
129.12
127.79
124.54
123.92
122.83
122.67
84.24
77.51
68.85
60.27
48.10
48.06
48.01
38.39
27.59
27.08
26.66
24.34
18.63
f1 (ppm)

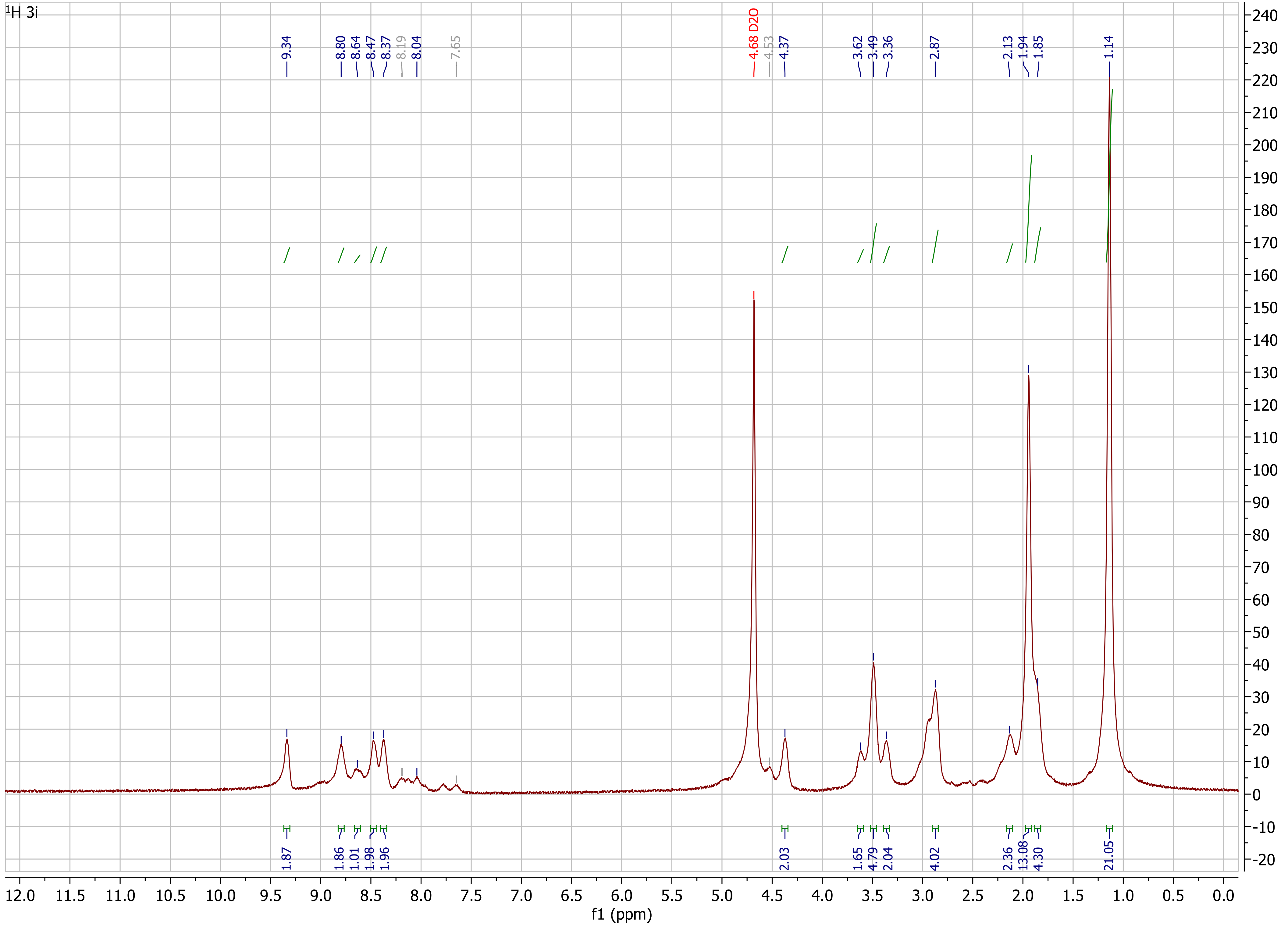

1H 3i
9.34
8.80
8.64
8.47
8.37
8.19
8.04
7.65
4.68 D2O
4.53
4.37
3.62
3.49
3.36
2.87
2.13
1.94
1.85
1.14
1.87
1.86
1.01
1.98
1.96
2.03
1.65
4.79
2.04
4.02
2.36
13.08
4.30
21.05
f1 (ppm)

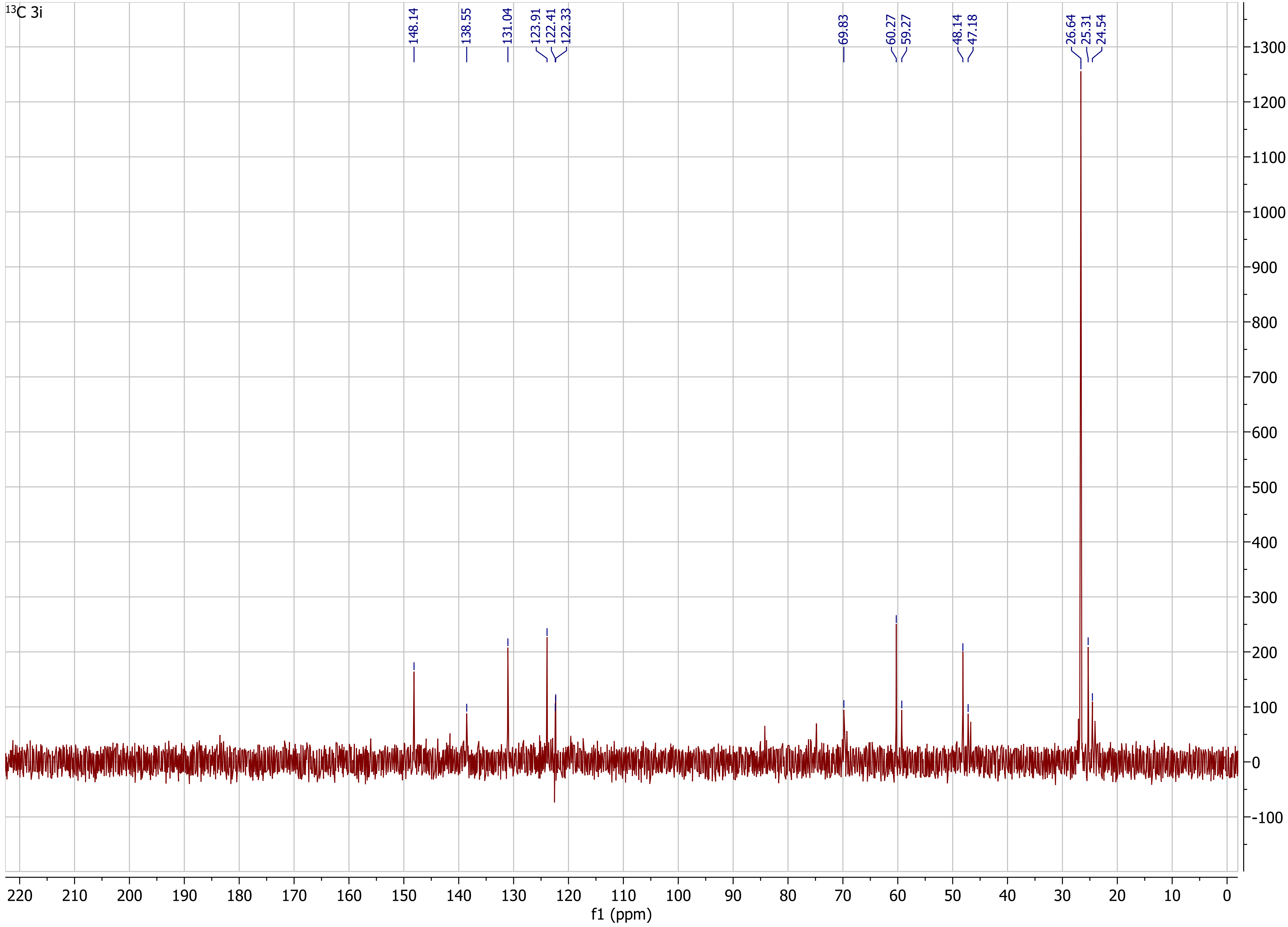

13C 3i
148.14
138.55
131.04
123.91
122.41
122.33
69.83
60.27
59.27
48.14
47.18
26.64
25.31
24.54
f1 (ppm)

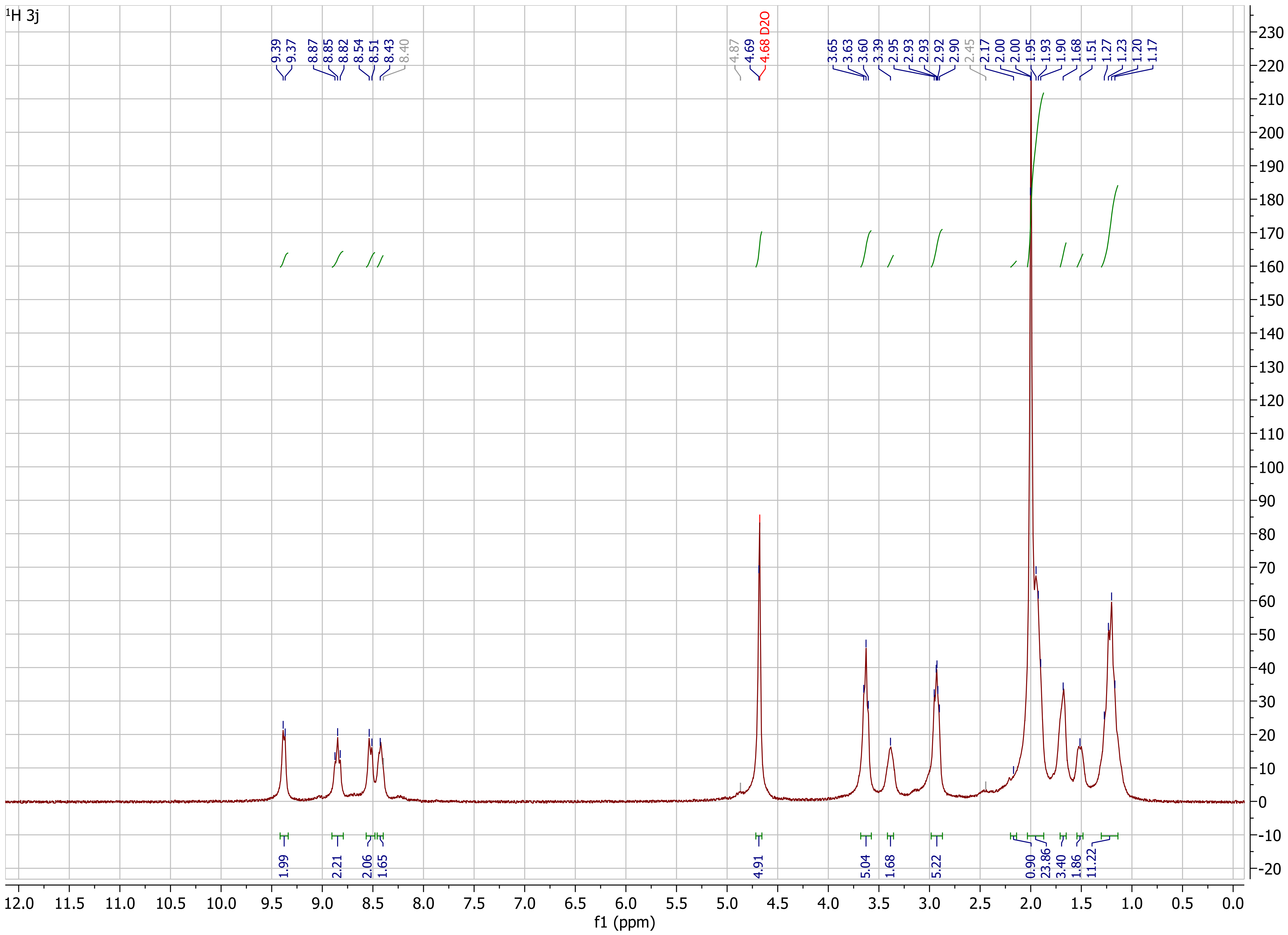

¹H 3j
9.39
9.37
8.87
8.85
8.82
8.54
8.51
8.43
8.40
4.87
4.69
4.68 D2O
3.65
3.63
3.60
3.39
2.95
2.93
2.93
2.92
2.90
2.45
2.17
2.00
2.00
1.95
1.93
1.90
1.68
1.51
1.27
1.23
1.20
1.17
1.99
2.21
2.06
1.65
4.91
5.04
1.68
5.22
0.90
23.86
3.40
1.86
11.22
f1 (ppm)

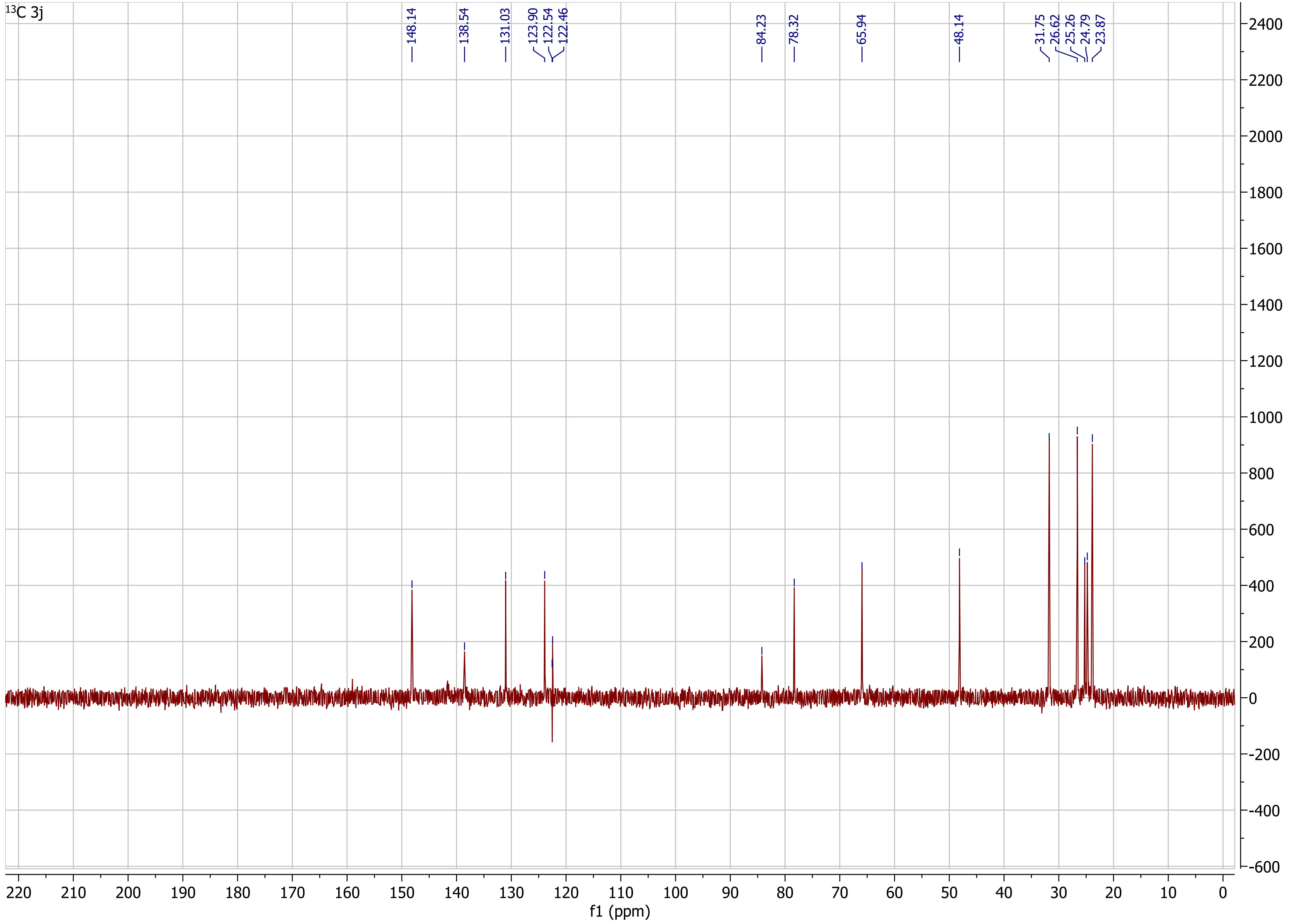

13C 3j
148.14
138.54
131.03
123.90
122.54
122.46
84.23
78.32
65.94
48.14
31.75
26.62
25.26
24.79
23.87
2400
2200
2000
1800
1600
1400
1200
1000
800
600
400
200
0
-200
-400
-600
220 210 200 190 180 170 160 150 140 130 120 110 100 90 80 70 60 50 40 30 20 10 0
f1 (ppm)

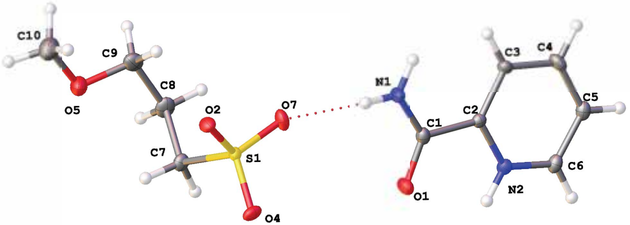

C10
C9
C8
O5
O2
O7
C7
S1
O4
N1
C1
C2
C3
C4
C5
C6
O1
N2

checkCIF/PLATON report

Structure factors have been supplied for datablock(s) 1622

THIS REPORT IS FOR GUIDANCE ONLY. IF USED AS PART OF A REVIEW PROCEDURE FOR PUBLICATION, IT SHOULD NOT REPLACE THE EXPERTISE OF AN EXPERIENCED CRYSTALLOGRAPHIC REFEREE.

No syntax errors found. CIF dictionary Interpreting this report

Datablock: 1622

Bond precision:	C-C = 0.0060 A		Wavelength=0.71073
Cell:	a=9.9079(5)	b=12.6799(6)	c=19.8194(10)
	alpha=90	beta=92.227(3)	gamma=90
Temperature:	100 K		

	Calculated	Reported
Volume	2488.1(2)	2488.1(2)
Space group	P 21	P 1 21 1
Hall group	P 2yb	P 2yb
Moiety formula	C4 H9 O4 S, C6 H7 N2 O	C4 H9 O4 S, C6 H7 N2 O
Sum formula	C10 H16 N2 O5 S	C10 H16 N2 O5 S
Mr	276.31	276.31
Dx,g cm-3	1.475	1.475
Z	8	8
Mu (mm-1)	0.276	0.276
F000	1168.0	1168.0
F000’	1169.60	
h,k,lmax	13,16,26	13,16,26
Nref	11848[6190]	29400
Tmin,Tmax	0.977,0.989	0.498,0.746
Tmin’	0.962	

Correction method= # Reported T Limits: Tmin=0.498 Tmax=0.746
AbsCorr = MULTI-SCAN

Data completeness= 4.75/2.48 Theta(max)= 27.875

R(reflections)= 0.0572(27603) wR2(reflections)= 0.1531(29400)

S = 1.055 Npar= 654

The following ALERTS were generated. Each ALERT has the format
test-name_ALERT_alert-type_alert-level.
Click on the hyperlinks for more details of the test.

Alert level B

```
PLAT097_ALERT_2_B Large Reported Max.  (Positive) Residual Density       1.72 eA-3
```

Alert level C

```
DIFMX02_ALERT_1_C  The maximum difference density is > 0.1*ZMAX*0.75
            The relevant atom site should be identified.
PLAT094_ALERT_2_C Ratio of Maximum / Minimum Residual Density ....       2.30 Report
PLAT340_ALERT_3_C Low Bond Precision on  C-C Bonds ..............    0.00604 Ang.
PLAT790_ALERT_4_C Centre of Gravity not Within Unit Cell: Resd.  #          1 Note
              C4 H9 O4 S
PLAT911_ALERT_3_C Missing FCF Refl Between Thmin & STh/L=    0.600         33 Report
```

Alert level G

```
PLAT007_ALERT_5_G Number of Unrefined Donor-H Atoms .............         12 Report
PLAT033_ALERT_4_G Flack x Value Deviates > 3.0 * sigma from Zero .      0.114 Note
PLAT072_ALERT_2_G SHELXL First  Parameter in WGHT  Unusually Large       0.11 Report
PLAT432_ALERT_2_G Short Inter X...Y Contact  O1       ..C6A      .       2.98 Ang.
                                                  x,y,z  =      1_555 Check
PLAT432_ALERT_2_G Short Inter X...Y Contact  O1A      ..C6       .       2.98 Ang.
                                                x,1+y,z  =      1_565 Check
PLAT432_ALERT_2_G Short Inter X...Y Contact  O1B      ..C6C      .       2.97 Ang.
                                                  x,y,z  =      1_555 Check
PLAT432_ALERT_2_G Short Inter X...Y Contact  O1C      ..C6B      .       2.98 Ang.
                                               x,-1+y,z  =      1_545 Check
PLAT432_ALERT_2_G Short Inter X...Y Contact  O4       ..C2       .       2.83 Ang.
                                          -x,1/2+y,1-z  =      2_556 Check
PLAT720_ALERT_4_G Number of Unusual/Non-Standard Labels .........         24 Note
PLAT870_ALERT_4_G ALERTS Related to Twinning Effects Suppressed ..          ! Info
PLAT910_ALERT_3_G Missing # of FCF Reflection(s) Below Theta(Min).          1 Note
PLAT912_ALERT_4_G Missing # of FCF Reflections Above STh/L=  0.600          2 Note
PLAT913_ALERT_3_G Missing # of Very Strong Reflections in FCF ....          1 Note
PLAT933_ALERT_2_G Number of HKL-OMIT Records in Embedded .res File         13 Note
PLAT941_ALERT_3_G Average HKL Measurement Multiplicity ..........        4.8 Low
PLAT992_ALERT_5_G Repd & Actual _reflns_number_gt Values Differ by          9 Check
```

0 **ALERT level A** = Most likely a serious problem - resolve or explain
1 **ALERT level B** = A potentially serious problem, consider carefully
5 **ALERT level C** = Check. Ensure it is not caused by an omission or oversight
16 **ALERT level G** = General information/check it is not something unexpected

1 ALERT type 1 CIF construction/syntax error, inconsistent or missing data
9 ALERT type 2 Indicator that the structure model may be wrong or deficient
5 ALERT type 3 Indicator that the structure quality may be low
5 ALERT type 4 Improvement, methodology, query or suggestion
2 ALERT type 5 Informative message, check

It is advisable to attempt to resolve as many as possible of the alerts in all categories. Often the minor alerts point to easily fixed oversights, errors and omissions in your CIF or refinement strategy, so attention to these fine details can be worthwhile. In order to resolve some of the more serious problems it may be necessary to carry out additional measurements or structure refinements. However, the purpose of your study may justify the reported deviations and the more serious of these should normally be commented upon in the discussion or experimental section of a paper or in the "special_details" fields of the CIF. checkCIF was carefully designed to identify outliers and unusual parameters, but every test has its limitations and alerts that are not important in a particular case may appear. Conversely, the absence of alerts does not guarantee there are no aspects of the results needing attention. It is up to the individual to critically assess their own results and, if necessary, seek expert advice.

Publication of your CIF in IUCr journals

A basic structural check has been run on your CIF. These basic checks will be run on all CIFs submitted for publication in IUCr journals (*Acta Crystallographica*, *Journal of Applied Crystallography*, *Journal of Synchrotron Radiation*); however, if you intend to submit to *Acta Crystallographica Section C* or *E* or *IUCrData*, you should make sure that full publication checks are run on the final version of your CIF prior to submission.

Publication of your CIF in other journals

Please refer to the *Notes for Authors* of the relevant journal for any special instructions relating to CIF submission.

PLATON version of 28/11/2022; check.def file version of 28/11/2022

Datablock 1622 - ellipsoid plot

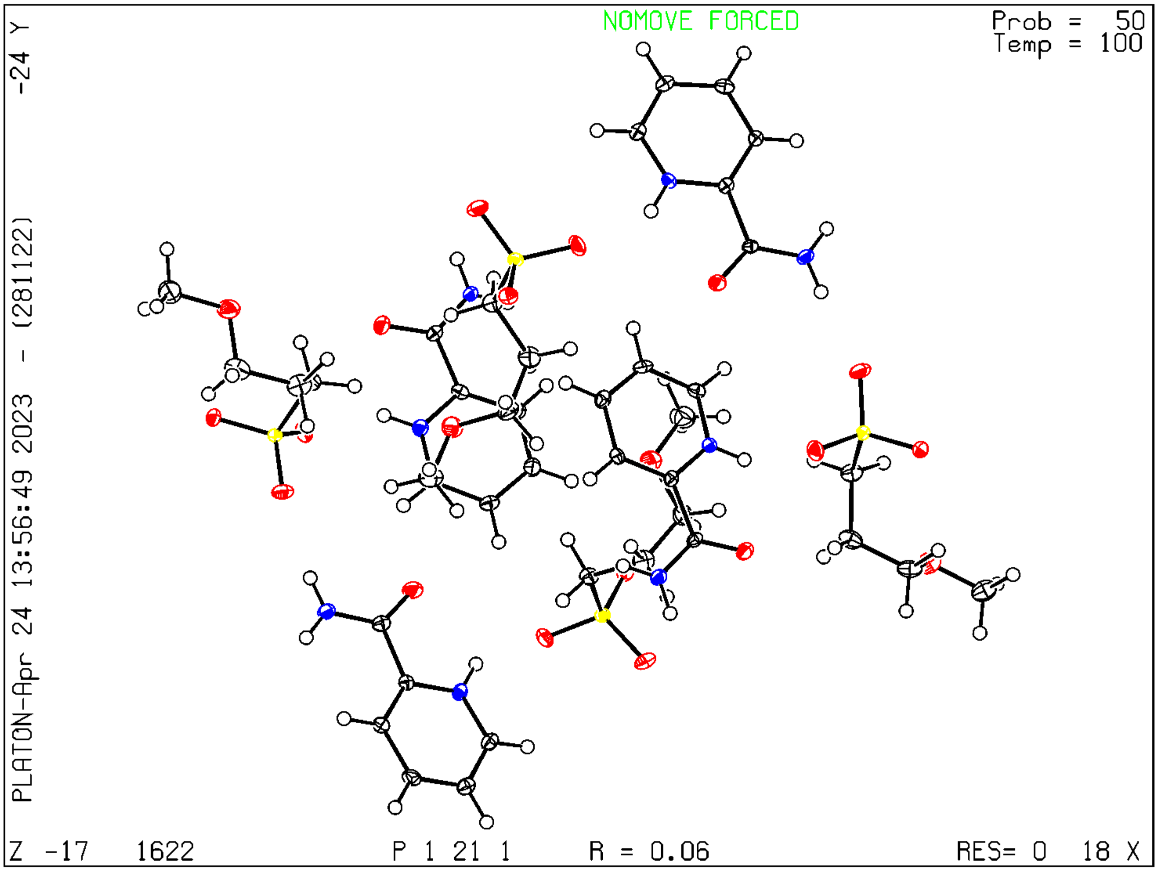

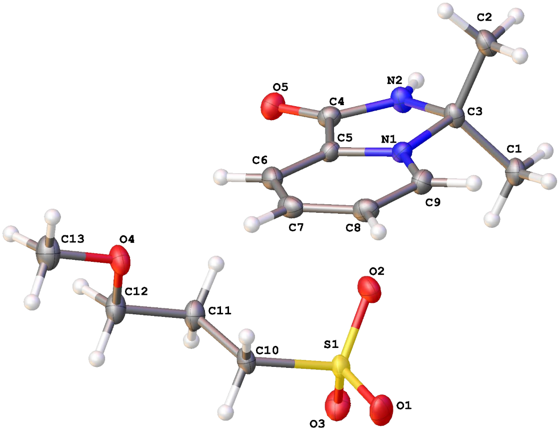

C2
O5
N2
C4
C3
C5
N1
C1
C6
C9
C7
C8
C13
O4
O2
C12
C11
C10
S1
O1
O3

checkCIF/PLATON report

Structure factors have been supplied for datablock(s) 2622

THIS REPORT IS FOR GUIDANCE ONLY. IF USED AS PART OF A REVIEW PROCEDURE FOR PUBLICATION, IT SHOULD NOT REPLACE THE EXPERTISE OF AN EXPERIENCED CRYSTALLOGRAPHIC REFEREE.

No syntax errors found. CIF dictionary Interpreting this report

Datablock: 2622

Bond precision:	C-C = 0.0052 A		Wavelength=0.71073
Cell:	a=12.422(2)	b=8.7237(15)	c=14.699(2)
	alpha=90	beta=109.106(6)	gamma=90
Temperature:	100 K		

	Calculated	Reported
Volume	1505.1(4)	1505.1(4)
Space group	P 21/n	P 1 21/n 1
Hall group	-P 2yn	-P 2yn
Moiety formula	C9 H11 N2 O, C4 H9 O4 S	2(C4 H9 O4 S), 2(C9 H11 N2 O)
Sum formula	C13 H20 N2 O5 S	C26 H40 N4 O10 S2
Mr	316.37	632.74
Dx,g cm-3	1.396	1.396
Z	4	2
Mu (mm-1)	0.238	0.238
F000	672.0	672.0
F000'	672.83	
h,k,lmax	16,11,19	16,11,19
Nref	3584	3544
Tmin,Tmax	0.986,0.993	0.595,0.746
Tmin'	0.976	

Correction method= # Reported T Limits: Tmin=0.595 Tmax=0.746
AbsCorr = MULTI-SCAN

Data completeness= 0.989 Theta(max)= 27.876

R(reflections)= 0.0728(3030) wR2(reflections)= 0.1902(3544)

S = 1.115 Npar= 193

The following ALERTS were generated. Each ALERT has the format
test-name_ALERT_alert-type_alert-level.
Click on the hyperlinks for more details of the test.

Alert level C

```
PLAT042_ALERT_1_C Calc. and Reported MoietyFormula Strings  Differ     Please Check
PLAT340_ALERT_3_C Low Bond Precision on  C-C Bonds ...............    0.00522 Ang.
PLAT906_ALERT_3_C Large K Value in the Analysis of Variance ......      6.736 Check
PLAT906_ALERT_3_C Large K Value in the Analysis of Variance ......      2.104 Check
PLAT911_ALERT_3_C Missing FCF Refl Between Thmin & STh/L=    0.600         34 Report
PLAT913_ALERT_3_C Missing # of Very Strong Reflections in FCF ....          5 Note
```

Alert level G

```
PLAT007_ALERT_5_G Number of Unrefined Donor-H Atoms ..............          1 Report
PLAT019_ALERT_1_G _diffrn_measured_fraction_theta_full/*_max < 1.0      0.998 Report
PLAT045_ALERT_1_G Calculated and Reported Z Differ by a Factor ...          2 Check
PLAT083_ALERT_2_G SHELXL Second Parameter in WGHT  Unusually Large       6.72 Why ?
PLAT432_ALERT_2_G Short Inter X...Y Contact  O2       ..C5       .       2.96 Ang.
                                                   x,y,z  =      1_555 Check
PLAT432_ALERT_2_G Short Inter X...Y Contact  O5       ..C5       .       3.01 Ang.
                                             2-x,1-y,1-z  =      3_766 Check
PLAT910_ALERT_3_G Missing # of FCF Reflection(s) Below Theta(Min).          1 Note
PLAT912_ALERT_4_G Missing # of FCF Reflections Above STh/L=  0.600          6 Note
PLAT933_ALERT_2_G Number of HKL-OMIT Records in Embedded .res File          4 Note
PLAT941_ALERT_3_G Average HKL Measurement Multiplicity ...........        3.8 Low
PLAT978_ALERT_2_G Number C-C Bonds with Positive Residual Density.          2 Info
```

0 **ALERT level A** = Most likely a serious problem - resolve or explain
0 **ALERT level B** = A potentially serious problem, consider carefully
6 **ALERT level C** = Check. Ensure it is not caused by an omission or oversight
11 **ALERT level G** = General information/check it is not something unexpected

3 ALERT type 1 CIF construction/syntax error, inconsistent or missing data
5 ALERT type 2 Indicator that the structure model may be wrong or deficient
7 ALERT type 3 Indicator that the structure quality may be low
1 ALERT type 4 Improvement, methodology, query or suggestion
1 ALERT type 5 Informative message, check

It is advisable to attempt to resolve as many as possible of the alerts in all categories. Often the minor alerts point to easily fixed oversights, errors and omissions in your CIF or refinement strategy, so attention to these fine details can be worthwhile. In order to resolve some of the more serious problems it may be necessary to carry out additional measurements or structure refinements. However, the purpose of your study may justify the reported deviations and the more serious of these should normally be commented upon in the discussion or experimental section of a paper or in the "special_details" fields of the CIF. checkCIF was carefully designed to identify outliers and unusual parameters, but every test has its limitations and alerts that are not important in a particular case may appear. Conversely, the absence of alerts does not guarantee there are no aspects of the results needing attention. It is up to the individual to critically assess their own results and, if necessary, seek expert advice.

Publication of your CIF in IUCr journals

A basic structural check has been run on your CIF. These basic checks will be run on all CIFs submitted for publication in IUCr journals (*Acta Crystallographica*, *Journal of Applied Crystallography*, *Journal of Synchrotron Radiation*); however, if you intend to submit to *Acta Crystallographica Section C* or *E* or *IUCrData*, you should make sure that full publication checks are run on the final version of your CIF prior to submission.

Publication of your CIF in other journals

Please refer to the *Notes for Authors* of the relevant journal for any special instructions relating to CIF submission.

PLATON version of 28/11/2022; check.def file version of 28/11/2022

Datablock 2622 - ellipsoid plot

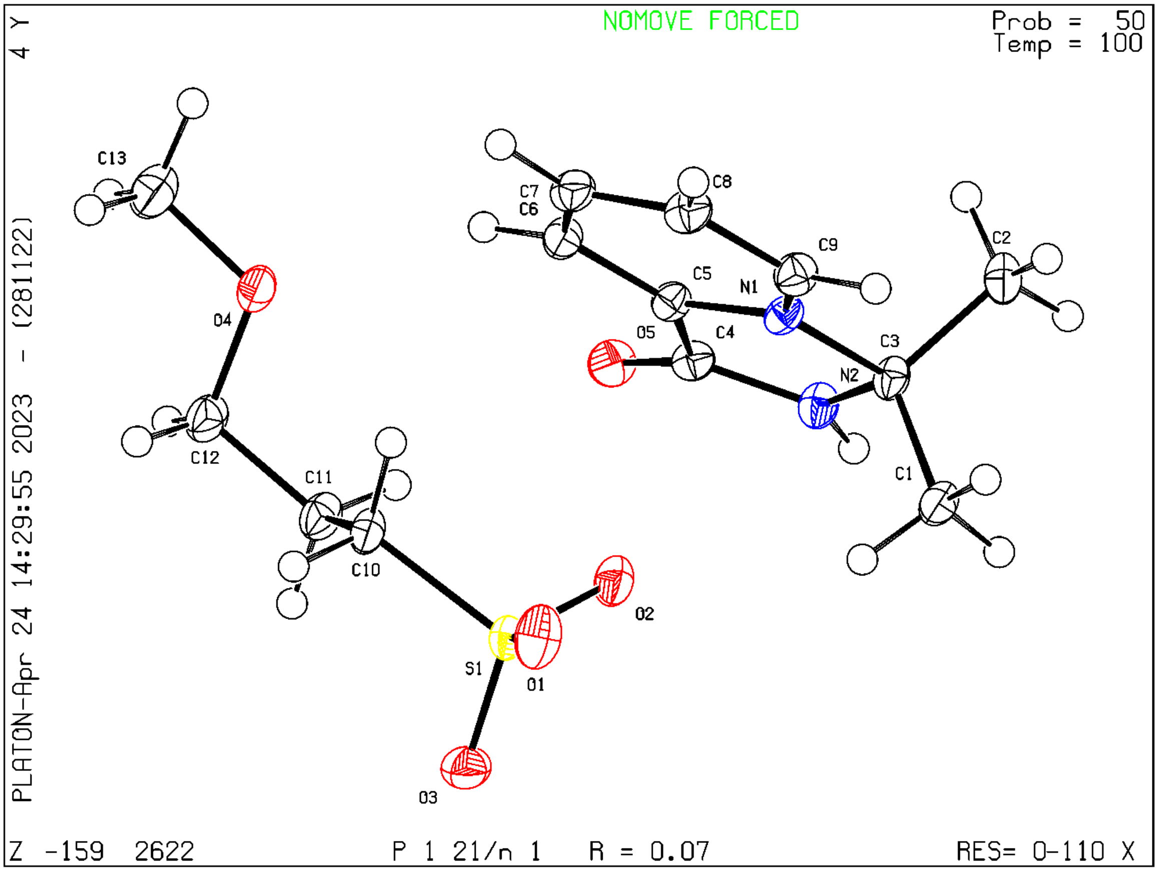

Supplement: Supplementary file 1 [file molecules-29-00206-s001.zip › molecules-2726002-supplementary.pdf]
